# Supplementary material for: DynamicAtlas: a morphodynamic atlas for Drosophila development
Source: Nat Methods. 2025 Dec 24;23(1):260–70. doi: 10.1038/s41592-025-02897-8 (PMC12791008; doi:10.1038/s41592-025-02897-8)
Supplement: Supplementary file 1 — Supplementary Notes 1 and 2, Tables 1 and 2, Notes 3–13, References, Python interface tutorial and MATLAB interface tutorial. [file 41592_2025_2897_MOESM1_ESM.pdf]

nature methods

Supplementary information https://doi.org/10.1038/s41592-025-02897-8

DynamicAtlas: a morphodynamic atlas for *Drosophila* development

In the format provided by the authors and unedited

Supplementary Information: Table of Contents

Supplementary Notes 1-2	2
Supplementary Tables 1-2	8
Supplementary Notes 3-13	11
Supplementary References	19
Python Interface Tutorial	21
MATLAB Interface Tutorial	38

Supplementary Note 1: Demo MATLAB code for core DynamicAtlas features

As discussed in greater depth in the online GitHub Documentation, the Matlab-based DynamicAtlas codebase enables the user to query datasets according to their genotype, fluorescent protein or antibody stain, and timestamp within the genotype's morphological timeline. An example pipeline that demonstrates core features for atlas lookup, in the form of a MATLAB script ("demo_dynamicAtlas_functionality.m" on the DynamicAtlas Github: `https://github.com/npmitchell/dynamicAtlas`), is reproduced below. For updated documentation, please refer to (*1*). A tutorial on using this demo script for a demo dataset is attached further below, on Page 48.

```
%% Demo DynamicAtlas Functionality
%
% Demo script for the core functions of the Matlab-based atlas
   code:
% creating an altas structure, creating a master timeline of
   live data,
% and timestamping fixed data onto the master timeline.
%
% Walkthrough is intended for use on the demo dataset with file
    name
% 'DEMO_DATASET.tar.lz4', on the Zenodo repository at the
   following URL:
%
% 'https://doi.org/10.5281/zenodo.14792464'
%
% See corresponding tutorial document included with the
   publication.
%
% NPMitchell 2020,
% Edited by Vishank Jain-Sharma 2025

%% I. Clear our environment

%clear workspace
clear

%clear command window
clc

%close open figures
```

```
close all


%% II. Add paths to atlas data and code to MATLAB search path

%Path to the folder containing the data

%atlasPath = '/PATH/TO/ATLAS/DATA/FOLDER';
%e.g.
atlasPath = '/Users/Vishank/Documents/DynamicAtlas_Demo';

%Path to the folder containing the code

%codePath = '/PATH/TO/ATLAS/CODE/FOLDER';
%e.g.
codePath = '/Users/Vishank/Documents/Dynamic_Atlas_Testing/
   dynamicAtlas';

%adds atlas path and subfolders to MATLAB search path
addpath(genpath(codePath));

%adds code path and subfolders to MATLAB search path
addpath(genpath(codePath));
pkgDir = fullfile(codePath, '+dynamicAtlas');
cd(pkgDir);
addpath(genpath('./'));
cd(atlasPath)

%% III. Build the dynamicAtlas object
%
% Build dynamic atlas with all genotypes in the atlasPath using
   :
% da = dynamicAtlas.dynamicAtlas(atlasPath);
%
% Or, choose which genotypes to include in atlas as below.
% By default, all are included

%options specifying how to construct the atlas
Options = struct();

%method of timeline construction
```

```
Options.timeLineMethod = 'realspace';

%constructing the atlas with wildtype genotypes (WT)
da = dynamicAtlas.dynamicAtlas(atlasPath, {'WT'}, Options);

%% IV. List the properties of the dynamic atlas

properties(da)

%% V. List the methods of the dynamic atlas

methods(da)

%% VI. Grab all the metadata from a label, and store in a 'queriedSample'

%data from the wildtype (WT) genotype containing the Runt label
qs = da.findGenotypeLabel('WT','Runt');

%% VII. Load in the genotype's data into the queriedSample object

%displays metadata of the queriedSample
qs.meta

%loads in the corresponding data
qs.getData()

%displays the data that was loaded
qs.data

%% VIII. Compute particle-image velocimetry (PIV) on the above data

%options specifying how to compute the PIV
Options = struct();

%overwrite existing PIV computations
Options.overwrite = false;

%computes PIV on the queriedSample data
```

```
qs.ensurePIV(Options);

%% IX. Grab all the data associated with a single embryo

%ID of the specific embryo dataset we are querying
embryoID = '202001141730';

%queriedSample with just this embryo's metadata
qs2 = da.findEmbryo(embryoID) ;

%gets the data of this embryo
qs2.getData();

%% X. Use dynamic datasets to build a master timeline
%
% Aligning dynamic run nanobody data against each other

%options specifying how to compute the timeline
Options = struct();

%saving the plots generated whle computing the timeline
Options.save_images = 1;

%makes the master timeline from the live WT datasets with the
   Runt label
da.makeMasterTimeline('WT','Runt', Options)


%% XI. Timestamp fixed data against the master timeline
%
% Timestamping Runt fixed samples against the master timeline
% Options can be passed through a struct if desired.

%path to the folder of the embryo chosen as the master timeline
    designee
%(done earlier in the timeline creation block X, the folder
   will have
% 'master_timeline_designee.txt' within it)
masterDesigneeDir = '/Users/Vishank/Documents/DynamicAtlas_Demo
   /WT/Runt/202001141730';

```

```
%width(s) of Gaussian(s) to use while smoothing the images
sigmas = [20];
%steps used by the gradient during the computation
steps = [1];
%specifying to compute gradients only on the fixed datasets
%(only fixed are timestamped, so only necessary to compute these here)
fixedOnly = 1;

%makes gradient images of the fixed data to use in the alignment
makeGradientImages(da,'Runt',sigmas,steps,fixedOnly);

%options specifying how to timestamp the fixed samples
Options = struct();
%passes in the directory of the master timeline designee
Options.masterDesigneeDir = masterDesigneeDir;
%indicating that stripe information should be loaded in from the .mat
%variable stored in the folder
Options.loadStripeMat = 1;

%timestamps fixed samples with the Runt label to the master timeline
da.timeStamp('WT', 'Runt', Options)

disp('Demo done.')
```

Supplementary Note 2: Stocks represented in the atlas

Drosophila stocks contained in the atlas are listed in the attached Supplementary Table 1 (Pages 8-9), with stock name and origin included. The table also enumerates which stocks were used to generate main text figure panels and supplementary videos. Several stocks were obtained from the Bloomington Drosophila Stock Center, which is abbreviated in the table by BDSC, and stock numbers for these lines are included. The atlas currently contains recordings of 500 unique fixed and live embryos. Since many embryos were imaged with multiple channels, this amounts to 808 different single-channel movies and images.

Stock Name	Origin	Used In
UAS-Baz::GFP	*Ref (2)*	
Klar SqhGFP Tl[rm9]	Gift from Wieschaus Lab	
Klar SqhGFP Spz[4]	Gift from Wieschaus Lab	Fig.4E
Oregon R	BDSC (#5)	
sqh[1] FRT101/FM7; P{w+ sqh-sqhAE::GFP}attP1	Gift from Adam Martin, *Ref (3)*	Figs.1A,2D,D",5A,B,C,D,E
Halo [DF2L] snail [IIG05] / CyO, Sqh-GFP	Gift from Adam Martin, *Ref (4)*	
Halo [DF2L] twist [ey53] / CyO, Sqh-GFP	Gift from Adam Martin, *Ref (4)*	Fig.4D
P{sGMCA-MoeGFP} on III	*Ref (5)*	Fig.3E'
endo-Ecad::GFP	BDSC (#60584)	Fig.3C,D,E'
w; ubi-DE-cad::GFP	*Ref (6)*	
w; ubi-DE-Cad::GFP shg[R69]; Sqh::mCherry[M1]	Gift from Adam Martin, *Ref (4)*	Fig.3E'
yw; sqh-sqh::mCherry[B1]	Gift from Adam Martin, *Ref (4)*	Fig.3E'
w ;; sqh-sqh::mCherry[A11]	Gift from Adam Martin, *Ref (4)*	Fig.3E'
Toll-8::SYFP2	Gift from Jennifer Zallen, *Ref (7)*	
Even-Skipped::SYFP2	*Ref (8)*	
P{ubi-GFP::rock}/TM3	Gift from Yohanns Bellaiche, *Ref (9)*	
H2A::RFP	Gift from Wieschaus Lab	Fig.3B,G',H,I
H2Av::mCherry	Streichan Lab, *Ref (10)*	Supplementary Video 1
sqh-utr::mCherry/ CyO	Gift from Thomas Lecuit, *Ref (11)*	Fig.3E'
yw sqh[1] FRT101/FM7;<br>P{w+ sqh-TS::GFP}attP40	Gift from Adam Martin, *Ref (3)*	Fig.3E'
sqh[AX3]; P[w+ sqh-gfp]42	*Ref (12)*	Fig.3E'
sqh–GFP::ROCK(K116A)	Gift from Jennifer Zallen, *Ref (13)*	
y1 w*; P{UAS-Lifeact::GFP}VIE-260B	BDSC (#35544)	
Runt::LlamaTag-GFP	Gift from Hernan Garcia, *Ref (14)*	Figs.1A,2A,A',B',B",3G,H
Tub67c-CAAX::mCherry<sqh3'<br>UTR(attp2)/Tm3,sb	Gift from Wieschaus Lab	Fig.3E',<br>Supplementary Video 2,<br>Supplementary Video 3
w; 48Y-GAL4; klar	BDSC (#4935), klar from Wieschaus Lab	Fig.6C,D
w[*]; P{w[+mC]=UAS-mCherry.CAAX.S}2	BDSC (#59021)	Fig.6B,B',C,D,G,H
w[*]; UASp-CIBN::pmGFP;<br>UASp-mCherry::CRY2-OCRL	Gift from Stephano de Renzis	

w[*]; UASp-CIBN::pmGFP; UASp-RhoGEF2-CRY2::mCherry	Gift from Stephano de Renzis	
y,P{w[+mC]=GAL4-Antp.P1.A}1,y[1]w[*]; wg[Sp-1]/CyO;;klar	BDSC (#26817), klar from Wieschaus Lab	
w;; Mef2-GAL4 klar	Gift from Lucy O'Brien, klar from Wieschaus Lab	
w; UAS-LifeAct::Ruby	BDSC (#35545)	Fig.6C,D
Hand-GFP; 4x HandGAL4; klar	Gift from Zhe Han, klar from Wieschaus Lab	Fig.6C,D
Bicoid[E1], Nanos[BN] / TM3, sb	Gift from Wieschaus Lab	Fig.4C
Bicoid[E1], Nanos[BN], Tsl[4] / TM3, sb	Gift from Wieschaus Lab	
Concertina[RC10] cn bw / CyO; T48 p[w+ sqhGFP]	Gift from Wieschaus Lab	
w; ΔJ29, Even-Skipped[r13] / CyO	Gift from Wieschaus Lab	Fig.4B
UAS–Fat2-RNAi	Gift from Sally Horne-Badovinac, *Ref (15)*	
w; Traffic Jam-GAL4; Gap43::mCherry, sqh::GFP	Gift from Adam Martin, *Ref (15)*	
UASp–Toll-2–HA	Gift from Jennifer Zallen, *Ref (7)*	
UASp–Toll8–HA	Gift from Jennifer Zallen, *Ref (7)*	
y w; P{UAS-runt.T}15	Gift from Peter Gergen, *Ref (16)*	
UAS-Even-skipped / TM6 P[rosy+{l(3)}]	Gift from Andrea Brand, *Ref (17)*	
Dpp[4] Snail[IIG05] / CyO	Gift from Wieschaus Lab	
Dpp[H46] wg[Sp-1] cn[1] bw[1]/CyO, P{dpp-P23}RP1	BDSC (#2061)	
y[1] w[*]; Pmatalpha4-GAL-VP1667; Pmatalpha4-GAL-VP1615	BDSC (#80361)	
w[*]; P{w[+mC]=His2Av-mRFP1}II.2	BDSC (#23651)	Fig.6C,D

Supplementary Table 1: *Drosophila* fly lines contained in the atlas.

Antibody Target:	Host:	Origin:	Dilution:	Imaged In:	Used In
Even-Skipped	Rabbit	Gift from Mark Biggin, Rabbit #10900	1:1000	WT,Tl[rm9],Spz[4], Even-Skipped OE	Figs.1B,E,2C,3G,3H
Runt	Guinea Pig	Gift from Wieschaus Lab	1:250	WT, Tl[rm9], Spz[4]	Figs.1B,E,2C,C”,3G,3H
Paired (Pax 3/7 DP312)	Mouse	Gift from Nipam Patel — *Ref (18)*	1:50-1:100	WT, Tl[rm9], Spz[4]	Figs.1B,E,3G,3H
Sloppy Paired 1	Rabbit	Gift from Mark Biggin, Rabbit #20257	1:1000	WT, Tl[rm9], Spz[4]	Figs.1B,E,3G,3H
Hairy	Rat	Gift from Wieschaus Lab, Rat #674	1:100	WT, Tl[rm9], Spz[4]	Figs.1B,E,3G,3H
Fushi Tarazu	Rabbit	Gift from Mark Biggin, Rabbit #11175	1:1000	WT, Tl[rm9], Spz[4]	Figs.1B,E,3G,3H
Toll-6 (residues 62 to 81, RPLTAGAGGDP-SLYDAPDDC)	Rat	Gift from Liqun Luo — *Ref (19)*	1:500	WT, Tl[rm9], Spz[4]	
Tartan	Rabbit	Gift from Wieschaus Lab — *Ref (20)*	1:1000	WT, Tl[rm9], Spz[4]	
Bazooka	Rabbit	Gift from Mo Weng — *Ref (21)*	1:1000	WT	
Neurotactin	Mouse	DSHB; Catalog: BP106	1:10	WT, Tl[rm9], Spz[4]	
GFP	Rabbit	Invitrogen; Catalog: A11122	1:1000	WT	
E-cadherin	Rat	DSHB; Catalog: DCAD2	1:50	WT,Tl[rm9],Spz[4], Dpp[H46]	

Supplementary Table 2: Primary antibodies.

Supplementary Note 3: Possible artifacts in cartographic lightsheet data

There are certain types of mostly harmless imaging artifacts which are peculiar to cartographic lightsheet imaging. For readers interested in the dynamic atlas datasets who are not familiar with looking at the output of lightsheet microscopy, we give a brief summary of these artifacts and how to interpret them.

- Fluorescent beads used for view registration included in image. Can be easily masked when carrying out quantifications.
- Unwrapping artifact. Our pipeline's second surface fitting step is based can lead to a lead to a small discontinuity at the line where the cylindrical projection is unwrapped. Can be avoided by rotating the image appropriately before the planar fitting step.
- Caustics. When the fitted surface has large curvature, caustics can arise in pullbacks from onion layers at a normal distance from the surface larger than the inverse curvature radius. Caustics typically look like small scale "blow-up" or "focusing" distortions.
- Suboptimal alignment. Since a lightsheet microscope should be manually re-aligned for each recording, suboptimal alignment can lead to decreased image quality (less crisp images).
- Dark lines due to bleaching during alignment. Fixed samples, in which fluorescence does not recover after bleaching, may show dark lines where the lightsheet was focused during the alignment phase.
- Large scale brightness gradients. Since the final image is a composite of multiple views, mistakes in microscope setup can lead to large-scale brightness artifacts. For example, if one of the two observation objectives is not working properly, one side of the embryo can appear brighter than the other. If this is detected, the datasets should not be used to measure large-scale intensity patterns.
- Onion layer selection. This is not an artifact per se, or only possible with lightsheet imaging. Depending on which "onion layer" along e.g. the apico-basal axis is selected, recordings of the same fluorescent marker and genotype can appear different.
- Fluctuations due to out-of-plane movement. If the number of surface-normal z-layers ("onion layers") included is small, out of plane movement can lead to rapid fluctuations in live movies.

Supplementary Note 4: Cell Tracking

We used our tracking to test the validity of our PIV results. We tracked cells in one of the atlas' dynamic datasets of a CAAX-mCherry embryo (Supplementary Video 2), in which cell membranes are visible (tracking overlay shown in Supplementary Video 3). To do so, we segmented the cells using `Ilastik` (*22*), using the random forest algorithm for a pixel classification on the cell membranes and against cell interiors. Using this segmentation, closest distance point-matching identified presumptive cell tracks. We then manually corrected these tracks in regions near the posterior pole. Approximately 1600 of the initial 6000 cells were tracked across the entire duration of the experiment, and only these cells were used to determine the scattered velocity field across the embryo surface. A linear interpolation of this scattered field then generated a field on the same lattice as the PIV to allow for comparison.

As shown in Extended Data Fig.3, the difference between velocity fields extracted from cell tracks and from PIV measured with 1 minute time resolution on the same embryo yielded values of velocity residual near ~ 0.1 for most of germband extension.

Supplementary Note 5: Instantaneous Flow

We compute the instantaneous flow of cells on the surface of the embryo, and use these flows to generate the correlation matrices visualized as heatmaps in Fig.3. We do so in an Eulerian reference frame. Instantaneous flow along the surface is defined as follows: denoting the position of a cell over time by $\vec{\mathbf{r}}_c(t)$, its instantaneous velocity $\vec{\mathbf{v}}_c(t)$ is defined as the time derivative of its position:

$$\vec{\mathbf{v}}_c(t) = \frac{d\vec{\mathbf{r}}_c(t)}{dt}$$

The cell's instantaneous flow along the surface is then given by the tangent component of the overall instantaneous velocity; denote this $\vec{\mathbf{v}}_c^{tan}(t)$. Let the function denoting unit normals to the embryo surface be $\hat{\mathbf{n}}(\vec{\mathbf{r}})$ (determined by the geometry of the surface). Let the normal vector to the surface at $\vec{\mathbf{r}}_c$ be $\hat{\mathbf{n}}_c = \hat{\mathbf{n}}(\vec{\mathbf{r}}_c)$. Then, $\vec{\mathbf{v}}_c^{tan}(t)$ is given by:

$$\vec{\mathbf{v}}_c^{tan} = \vec{\mathbf{v}}_c - (\vec{\mathbf{v}}_c \cdot \hat{\mathbf{n}}_c)\hat{\mathbf{n}}_c$$

We refer to the collection of cell velocities $\vec{\mathbf{v}}^{tan}(t)$ — the set of velocities $\vec{\mathbf{v}}_c^{tan}(t)$ for all cells c on the surface of the embryo — as the 'flow field'. We refer to the directions of velocities in $\vec{\mathbf{v}}^{tan}(t)$ as the 'flow pattern'.

Supplementary Note 6: Time-alignment for early embryo datasets

More information to elaborate on the details of time alignment discussed in the Methods section is given below.

Aligning live datasets using tissue deformation

To measure deformation, we need to decide which timepoint in each movie we consider as the un-deformed reference state and use as the starting point t_0^ω of integration. To make a choice of initial time that is the same across different embryos, we use the fact that the velocity field changes very rapidly at the onset of axis elongation. This timepoint of rapid change can be identified from the velocity field alone.

During the onset of ventral furrow formation, the tissue vorticity first shows clockwise (counter-clockwise) rotation in the left (right) posterior regions of the embryo surface, then switches sign to counter-clockwise (clockwise) rotation in the left (right) posterior regions as the germband begins to extend. This local change in sign in vorticity is used to fix t_0^ω, which we use for time alignment. (Note that to be consistent with other publications (*20*), we have defined the 'onset of GBE' (used to label time 0 in Figs.3 and 5) as the time when the derivative of the root-mean-squared velocity is maximal. This time is close to t_0^ω, but the two are distinct quantities.)

We implement the following protocol:

1. Running PIV (*23*) on each dynamic dataset measures tissue flows. This is implemented via `qs.ensurePIV()`, for a `queriedSample` class instance `qs` that indexes the dynamic datasets to align.

2. We then identify the timepoint in which the vorticity changes sign via `qs.ensureT0V()`.

3. The tissue velocities are integrated forward and backward in time from t_0^ω to generate tissue displacement fields.

4. Using the tissue displacement fields, we can compare pairs of embryos in the ensemble for time alignment. As detailed below, we compute a similarity matrix and use a fast marching algorithm to define a correspondence curve linking the live datasets.

Because the tissue flows are quasi-stationary during GBE, a simple correlation of displacements does not sufficiently constrain the similarity heatmap $c(t^i, \tau)$ between embryo i and the ensemble average timecourse τ. Therefore, we multiply this correlation by a factor which is one if displacements of tissue patches are equal, on average. This composite measure, which we here refer to as 'similarity' is expressed as

$$C(\delta\vec{x}^i, \delta\vec{x}^j) \equiv c(\delta\vec{x}^i, \delta\vec{x}^j)\sin(2a), \tag{1}$$

where a is the slope of best fit between the two displacement fields $\delta\vec{x}^i$ and $\delta\vec{x}^j$. The trigonometric term in the similarity measure ensures that we register a similarity of $C \approx 1$ only when the displacements are not only proportional (with high correlation c), but also equal in magnitude.

All-to-all alignment and consensus algorithm

We used the following algorithm to generate all-to-all time alignment:

- Use the fast marching algorithm to obtain a correspondence curve for each pair of recordings in the ensemble E.
- Define a graph where nodes are frames from the movies in the ensemble. Two nodes are linked if (a) they are subsequent frames of the same movie or (b) they are frames from different movies matched by the correspondence curve (if a frame is matched "in the middle" of two frames in a different movie, two weighted edges are created). To each edge, we associate a "spring constant": the value of the spring constant is either a function of the frame-to-frame similarity if the edge links nodes of two different movies, or a fixed constant if the edge links nodes from the same movie.
- The graph therefore defines a spring network. Embed the graph in one dimension (i.e. assign a 1D position to every node) so as to minimize the elastic energy of the spring network.
- The resulting positions are the timestamps of all frames in the consensus timeline.

This wire network is illustrated in Extended Data Fig.1B. Currently, all twistPlus::Sqh movies were aligned using the all-to-all algorithm, see Extended Data Fig.1C-D, whereas the datasets aligned using Runt stripes were aligned to a reference recording.

Supplemenary Note 7: Spatiotemporal alignment of midgut morphogenesis

Given discretized embryo surfaces (i.e. meshes) $s_i(t)$ and $s_j(t')$ for two embryos i, j and times t, t', we use iterative closest point registration to find the rigid body transformation R of $s_j(t')$ that most closely aligns $s_j(t')$ to surface $s_i(t)$. Indexing the points sampling the surface $s_i(t)$ from 1 to P and indexing points sampling surface $s_j(t')$ from 1 to Q, we then compute the measure of mismatch of the registered surface $\tilde{s}_j(t') = R(s_j(t'))$ against the surface $s_i(t)$ as

$$\Delta\left[s_i(t), \tilde{s}_j(t')\right] \equiv \sum_{p=1}^{P} ||\tilde{s}_j(t') - s_i(t)||^2/P, \tag{2}$$

and the mismatch of the surface $s_i(t)$ against the registered surface $R(s_j(t'))$ as

$$\Delta\left[\tilde{s}_j(t'), s_i(t)\right] \equiv \sum_{q=1}^{Q} ||s_i(t) - \tilde{s}_j(t')||^2/Q, \tag{3}$$

where the norm $||\tilde{s}_j - s_i||$ is the Euclidean distance between a given point p in s_i and the nearest point to p that lies in s_j. This measure of mismatch, δ, therefore represents the average squared

distance from one shape to another. We use both the distance from $\tilde{s}_j$ to s_i and the distance s_i to $\tilde{s}_j$ since these are not generally equal, and therefore we take the geometric mean to define a composite mean distance between shapes

$$d_{ij}^2 \equiv \sqrt{\Delta\left[s_i(t), \tilde{s}_j(t')\right]\, \Delta\left[\tilde{s}_j(t'), s_i(t)\right]}. \tag{4}$$

Fast marching through the landscape of residuals in (t, t') space defines a correspondence between timelines.

Within our ensemble of extracted and spatiotemporally-aligned midgut surfaces, we measure the mean distance between pairwise gut surfaces. In detail, for each aligned surface pair $s_i(\tau)$ and $s_j(\tau)$ at morphological time τ, we have the mean squared distance between the shapes computed before, d_{ij}^2 (see Eqn. 4). The mean distance between midgut shapes is taken as $d_{ij} \equiv \sqrt{d_{ij}^2}$. This distance increases over the timecourse of midgut constrictions, reflecting the increasing uniqueness of each embryo's midgut shape (Extended Data Fig.9).

Supplementary Note 8: Tissue cartography

Briefly, the ImSAnE workflow contains three steps: (1) surface detection, which segments the 3D dataset into an "inside" and an "outside", (2) surface fitting, which fits a smooth surface through the detected surface, and (3) cartography, which projects the 3D intensity data onto the fitted surface. For most datasets capturing *Drosophila* body axis elongation, we used the `morphsnakesDetector` – which was added to ImSAnE as part of the work presented in (*24*) – to extract the surface. We then used the `spherelikeFitter` to map layers at variable distance from the surface normal ('onionLayers') to the plane and performed an additional step using the `planarFitter` to extract the apical surface of the early embryo. Finally, we rotated the pullback images along their (periodic) dorsoventral axis such that the ventral midline is placed at the edges of the image.

These cartographic steps enabled spatial alignment across datasets. Variability in the geometry of these embryos could serve as a source of error in the alignment. We therefore examined a subset of WT embryos from the atlas — those used to generate Figure 3E' (enumerated in Supplementary Table 1 above) — and measured the embryo length as a way to gauge differences in embryo geometry. As shown in Extended Data Figure 1, embryo lengths varied little, with standard deviation only $\sim$4% of the mean, indicating that errors introduced during our map alignment are small.

For midgut surfaces we used `TubULAR` (*24*) as an extension to `ImSAnE` to extract a surface that penetrates $\sim$2.5 μm within the apical surface of the endoderm, along the surface that intersects endodermal nuclei. To extract tissue velocity fields and covariant measures of deformation, we first performed particle image velocimetry (*23*) in `TubULAR`'s (s, ϕ) pullback parameterization, pushed the resulting velocity vectors into the 3D lab frame, and used the discrete exterior calculus package (`DEC`) included with `TubULAR` for further processing.

Supplementary Note 9: Tissue cartography variation

As shown in Extended Data Fig.1E-F, the embryo length varied very little (with only a 4% standard deviation). This suggests that errors from spatially aligning all embryos to a standard reference surface for GBE analysis carries comparably small error.

Supplementary Note 10: Correlations of Velocity Fields

We describe the similarity measures that we use to correlate two velocity fields below.

We denote by x_1 and x_2 the parametrized coordinates of the surface with total area A and induced metric tensor field $\overleftrightarrow{g}(x_1, x_2)$. The metric tensor is used to mathematically compensate for distortions in the mapping of the embryo surface to two dimensions. Additionally, let

$$\langle \vec{v}, \vec{w} \rangle = \sum_{i=1}^{2} \sum_{j=1}^{2} g_{ij} v_i w_j \tag{5}$$

denote the inner product of two vector fields $\vec{v}_{e_1}$ and $\vec{v}_{e_2}$ on this surface, weighting by g_{ij} to account for map distortions.

Average angle cosine

We point-wise compute the angles between the two fields, and spatially average them to get a single number. We compute the cosines of the angles so that the result is normalized between -1 (anti parallel) and 1 (parallel):

$$\rho^{\vec{v}}(\vec{v}_{e_1}, \vec{v}_{e_2}) = \frac{1}{A} \int \int \frac{\langle \vec{v}_{e_1}(x_1, x_2), \vec{v}_{e_2}(x_1, x_2) \rangle}{\sqrt{\langle \vec{v}_{e_1}(x_1, x_2), \vec{v}_{e_1}(x_1, x_2) \rangle} \sqrt{\langle \vec{v}_{e_2}(x_1, x_2), \vec{v}_{e_2}(x_1, x_2) \rangle}} \, dx_1 \, dx_2 \tag{6}$$

Vorticity Correlation

We compute the curl of the two vector fields (the vorticity), and then compute the correlation coefficient for the two resultant scalar fields. In the language of differential geometry, the curl operator creates an anti-symmetric differential form which can be computed using the same coordinate-based formula for flat and curved surfaces. The curl is computed as

$$\omega_{e_i}(x_1, x_2) = \nabla \times \vec{v}_{e_1}(x_1, x_2) \tag{7}$$

The spatial average of the vorticity field is $\overline{\omega_{e_i}} = \frac{1}{A} \int \int w_v(x_1, x_2) \, dx_1 \, dx_2$. In principle, this spatial average vanishes (integrating a derivative over a closed surface), but we do not include the poles into the spatial average due to poor image quality and high map distortion there.

We then define the correlation between two vorticity fields as

$$\rho^{\omega}(\omega_{e_1}, \omega_{e_2}) = \frac{\int\int(\omega_{e_1}(x_1,x_2) - \overline{\omega_{e_1}})(\omega_{e_2}(x_1,x_2) - \overline{\omega_{e_2}})\,dx_1\,dx_2}{\sqrt{\int\int(\omega_{e_1}(x_1,x_2) - \overline{\omega_{e_1}})^2\,dx_1\,dx_2}\sqrt{\int\int(\omega_{e_2}(x_1,x_2) - \overline{\omega_{e_2}})^2\,dx_1\,dx_2}}, \quad (8)$$

Here again, $\rho^{\omega} \in [-1, 1]$, with -1 signifying the vorticity fields are perfectly anti-correlated, and 1 signifying the vorticity fields are perfectly correlated.

Normalized Residual

To measure a residual (dissimilarity) between two flow fields, we use an expression defined in (*25*). Using the definition in 5, define the quantity v_{RMS} for a flow-field $\vec{v}$ by:

$$v_{RMS} = \sqrt{\frac{1}{A}\int\int\langle\vec{v}(x_1,x_2), \vec{v}(x_1,x_2)\rangle\,dx_1\,dx_2} \quad (9)$$

Then, define:

$$\vec{\delta}(x_1,x_2) = \frac{1}{2}\left(\frac{\vec{v}_{e_1}(x_1,x_2)}{v_{RMS}} - \frac{\vec{v}_{e_2}(x_1,x_2)}{w_{RMS}}\right) \quad (10)$$

and

$$r(\vec{v}_{e_1}, \vec{v}_{e_2}) = \frac{1}{A}\int\int\langle\vec{\delta}(x_1,x_2), \vec{\delta}(x_1,x_2)\rangle\,dx_1\,dx_2 \quad (11)$$

With this construction, $r \in [0, 2]$, with 0 signifying that $\vec{v}_{e_1}$ is strictly proportional to $\vec{v}_{e_2}$, and 2 signifying strict anti-proportionality. Note that to compare this to the usual $[-1, 1]$ range from full anti-correlation to full correlation, one may simply subtract this measure from 1.

Supplementary Note 11: Computing ensemble-averaged velocity correlations

Once fixed alignment is performed on an ensemble E (see Rigid Time Alignment section in Methods), a common aligned time coordinate t is created (with some total time T in common among all embryos in E), and one has a distinct spatial velocity field $\vec{v}_e(\vec{\mathbf{r}}, t)$ for each timestamp t and embryo e. One can now average either over time or over the ensemble members. The first yields the time-averaged flow-field $\langle\omega\rangle_T$ for a single embryo across total time T, and the second is the ensemble-averaged flow-field $\langle\omega(t)\rangle_E$ at a given (aligned) time t across the whole ensemble E. The set of time averaged fields can then be compared to the ensemble-averaged flows over time, a procedure which was performed systematically in Figure 3E' to illustrate the similarity between these two sets of flow field averages.

Supplementary Note 12: Alignment of Mutant Ensembles

Beyond wild-type embryos, our time alignment methods can also be used to align ensembles of mutant embryos, though with additional caveats which we clarify below. We do not align data in chronological time, but rather align data in morphological time. Our methods depend on the assumption that a deterministic feature of development, which reproducibly changes with time across samples, can be used to timestamp embryos (for example — but not limited to — gene expression patterns (Fig.2A-C”), tissue deformations (Fig.2D-D”), and organ geometries (Fig.6C). If a mutant exhibits such a feature, time aligning embryos of this genotype to each other is feasible.

However, we caution that a continuous time alignment between embryos of different genotypes is generally *not* well-defined. When embryos do not proceed through the same morphological stages, time-stamps as defined by physical features are no longer universal.

In special cases, these comparisons can still be performed productively. In particular, one may perform a continuous morphological time alignment on embryos of two genotypes during a period of time before the embryos diverge in their morphological trajectories.

If embryos diverge immediately, continuous time alignment is not feasible, but discrete time comparisons may still be possible. For example, if a series of identical morphological events occurs in two embryos (such as in Fig.4A), one may perform a rigid time alignment to the first corresponding event in both embryos, and compare properties of their subsequent development. This analysis may prove especially useful when comparing ensemble-averaged properties of ensembles with distinct genotypes (such as in Fig.4B-E).

Supplementary Note 13: Computing average RMS velocity curves

To calculate the spatially averaged tissue flow velocity (i.e. RMS velocity, see Fig.3I and Fig.5B), we weighted the grid points at which the PIV fields are evaluated by their area, which can be computed from the induced metric. This ensures that all areas contribute equally and eliminates potential map distortion. Using equation 9:

$$v_{RMS}^{weighted} = \sqrt{\frac{1}{A}\sum_{x_1}\sum_{x_2}\langle\vec{v}(x_1,x_2),\vec{v}(x_1,x_2)\rangle\cdot\sqrt{\det\left(\overset{\leftrightarrow}{g}(x_1,x_2)\right)}} \tag{12}$$

Supplementary References

1. Mitchell, N., Jain-Sharma, V. & Streichan, S. dynamicAtlas. Github `https://github.com/npmitchell/dynamicAtlas` (2022).

2. Krahn, M. P., Klopfenstein, D. R., Fischer, N. & Wodarz, A. Membrane targeting of Bazooka/PAR-3 is mediated by direct binding to phosphoinositide lipids. *Current Biology* **20**, 636–642 (2010).

3. Vasquez, C. G., Tworoger, M. & Martin, A. C. Dynamic myosin phosphorylation regulates contractile pulses and tissue integrity during epithelial morphogenesis. *Journal of Cell Biology* **206**, 435–450 (2014).

4. Martin, A. C., Kaschube, M. & Wieschaus, E. F. Pulsed contractions of an actin–myosin network drive apical constriction. *Nature* **457**, 495–499 (2009).

5. Kiehart, D. P., Galbraith, C. G., Edwards, K. A., Rickoll, W. L. & Montague, R. A. Multiple forces contribute to cell sheet morphogenesis for dorsal closure in *Drosophila*. *The Journal of Cell Biology* **149**, 471–490 (2000).

6. Oda, H. & Tsukita, S. Real-time imaging of cell-cell adherens junctions reveals that *Drosophila* mesoderm invagination begins with two phases of apical constriction of cells. *Journal of Cell Science* **114**, 493–501 (2001).

7. Paré, A. C. *et al.* A positional Toll receptor code directs convergent extension in *Drosophila*. *Nature* **515**, 523–527 (2014).

8. Ludwig, M. Z., Manu, Kittler, R., White, K. P. & Kreitman, M. Consequences of eukaryotic enhancer architecture for gene expression dynamics, development, and fitness. *PLoS Genetics* **7**, e1002364 (2011).

9. Bardet, P.-L. *et al.* PTEN controls junction lengthening and stability during cell rearrangement in epithelial tissue. *Developmental Cell* **25**, 534–546 (2013).

10. Krzic, U., Gunther, S., Saunders, T. E., Streichan, S. J. & Hufnagel, L. Multiview light-sheet microscope for rapid *in toto* imaging. *Nature Methods* **9**, 730–733 (2012).

11. Rauzi, M., Lenne, P.-F. & Lecuit, T. Planar polarized actomyosin contractile flows control epithelial junction remodelling. *Nature* **468**, 1110–1114 (2010).

12. Royou, A., Sullivan, W. & Karess, R. Cortical recruitment of nonmuscle myosin II in early syncytial *Drosophila* embryos: its role in nuclear axial expansion and its regulation by Cdc2 activity. *Journal of Cell Biology* **158**, 127–137 (2002).

13. de Matos Simões, S. *et al.* Rho-Kinase directs Bazooka/Par-3 planar polarity during *Drosophila* axis elongation. *Developmental Cell* **19**, 377–388 (2010).

14. Bothma, J. P., Norstad, M. R., Alamos, S. & Garcia, H. G. LlamaTags: A versatile tool to image transcription factor dynamics in live embryos. *Cell* **173**, 1810–1822 (2018).

15. Chanet, S. *et al.* Actomyosin meshwork mechanosensing enables tissue shape to orient cell force. *Nature Communications* **8**, 15014 (2017).

16. Tracey Jr, W. D., Pepling, M. E., Horb, M. E., Thomsen, G. H. & Gergen, J. P. A *Xenopus* homologue of *aml-1* reveals unexpected patterning mechanisms leading to the formation of embryonic blood. *Development* **125**, 1371–1380 (1998).

17. Brand, A. H. & Perrimon, N. Targeted gene expression as a means of altering cell fates and generating dominant phenotypes. *Development* **118**, 401–415 (1993).

18. Davis, G. K., D'Alessio, J. A. & Patel, N. H. Pax3/7 genes reveal conservation and divergence in the arthropod segmentation hierarchy. *Developmental Biology* **285**, 169–184 (2005).

19. Ward, A., Hong, W., Favaloro, V. & Luo, L. Toll receptors instruct axon and dendrite targeting and participate in synaptic partner matching in a *Drosophila* olfactory circuit. *Neuron* **85**, 1013–1028 (2015).

20. Lefebvre, M. F., Claussen, N. H., Mitchell, N. P., Gustafson, H. J. & Streichan, S. J. Geometric control of myosin II orientation during axis elongation. *eLife* **12**, e78787 (2023).

21. Gu, L. *et al.* A novel protein Moat prevents ectopic epithelial folding by limiting Bazooka/Par3-dependent adherens junctions. *Molecular Biology of the Cell* **35**, ar110 (2024).

22. Berg, S. *et al.* ilastik: interactive machine learning for (bio)image analysis. *Nature Methods* **16**, 1226–1232 (2019).

23. Thielicke, W. & Stamhuis, E. PIVlab – Towards User-friendly, Affordable and Accurate Digital Particle Image Velocimetry in MATLAB. *Journal of Open Research Software* **2**, e30 (2014).

24. Mitchell, N. P. & Cislo, D. J. TubULAR: tracking in toto deformations of dynamic tissues via constrained maps. *Nature Methods* **20**, 1980–1988 (2023).

25. Streichan, S. J., Lefebvre, M. F., Noll, N., Wieschaus, E. F. & Shraiman, B. I. Global morphogenetic flow is accurately predicted by the spatial distribution of myosin motors. *eLife* **7**, e27454 (2018).

DynamicAtlas tutorial for Python-based interface

[1]:
```python
import numpy as np
import pandas as pd
import matplotlib.pyplot as plt
from pathlib import Path
from scipy import io
```

[2]:
```python
pd.set_option("display.notebook_repr_html", True)
```

1 Tutorial for querying the Drosophila Morphodynamic Atlas using python

The Drosophila morphodynamic atlas us a collection of 479 in-toto lightsheet-microscopy recordings of the Drosophila embryo at the blastoderm stage (stages 6-9). The atlas comprises 18 different genotypes.

The atlas data, including a description of the files and this tutorial, can be found on Dryad, while the methodology and use of the atlas is the subject of the publication Morphodynamic Atlas for Drosophila Development (Mitchell et al, 2022).
Please see the `README.md` file on Dryad for details on the dataset.

This tutorial explains how to query the atlas for specific datasets and load the results into Python for inspection and analysis. You need the following python modules: `pandas`, `numpy`, `matplotlib`, and `scipy`. You further need the atlas database `Morphodynamic_Atlas.csv`, as well as the data you would like to load. The complete atlas is several 100GB large, so you can download just parts of it, e.g. for specific genotypes. All files can be found on the Dryad repository.

The code for querying the atlas uses the `pandas` (for database processing) and `pathlib` (for finding files on your hard drive) libraries. Both libraries are well-documented and popular and Google should be able to answer your usage questions.

We start by loading the database as a `pandas.DataFrame`. You need to place `Morphodynamic_Atlas.csv` in the same folder as this jupyter notebook. `pandas` is a powerful library for relational ("spreadsheet-like") data in python and will allow us to select, group, and analyze the datasets in the atlas.

[3]:
```python
atlas_database = pd.read_csv('Morphodynamic_Atlas.csv')
atlas_database
```

[3]:

```
                                              Embryo_ID  \
0    ID of embryo (time of recording), in YYYYMMDDH…
1                                          202011021800
2                                          202011031830
3                                          202011071345
4                                          202011301820
..                                                  …
786                                        201908221210
787                                        201908221228
788                                        201908221236
789                                        201908221242
790                                        201908221248

                                               Genotype  \
0    Genotype. For organization purposes, recording…
1                  67-15_1to3copies_UAS-Even-Skipped
2                  67-15_1to3copies_UAS-Even-Skipped
3                  67-15_1to3copies_UAS-Even-Skipped
4             67-singlecopy_15-singlecopy_UAS-18W-HA
..                                                  …
786                                           toll[RM9]
787                                           toll[RM9]
788                                           toll[RM9]
789                                           toll[RM9]
790                                           toll[RM9]

                                            Fluorophore  \
0    Fluorescently tagged protein. For live recordi…
1                                               Sqh-GFP
2                                               Sqh-GFP
3                                               Sqh-GFP
4                                               Sqh-GFP
..                                                  …
786                                              Toll_8
787                                              Toll_8
788                                              Toll_8
789                                              Toll_8
790                                              Toll_8

                       Is_Live  \
0    Live or fixed recording
1                       True
2                       True
3                       True
4                       True
..                       …
786                    False
```

```
787                   False
788                   False
789                   False
790                   False

                                              Has_PIV  \
0    Whether PIV (tissue flow fields) are available…
1                                               False
2                                               False
3                                               False
4                                               False
..                                                …
786                                             False
787                                             False
788                                             False
789                                             False
790                                             False

                                           Has_PIVLab  \
0    Whether high-resolution PIV data (created by P…
1                                               False
2                                               False
3                                               False
4                                               False
..                                                …
786                                             False
787                                             False
788                                             False
789                                             False
790                                             False

                                           Timing_PIV  \
0    Timing based on PIV. Indicates frame of movie …
1                                                 NaN
2                                                 NaN
3                                                 NaN
4                                                 NaN
..                                                …
786                                               NaN
787                                               NaN
788                                               NaN
789                                               NaN
790                                               NaN

                                          Timing_Runt  \
0    Timing based on Runt stripe 7 deformation. Ind…
1                                                 NaN
```

```
2                                                    NaN
3                                                    NaN
4                                                    NaN
..                                                   …
786                                                  NaN
787                                                  NaN
788                                                  NaN
789                                                  NaN
790                                                  NaN

                                           Timing_Warped  \
0    Timing based on Runt 7 stripe deformation, aft…
1                                                    NaN
2                                                    NaN
3                                                    NaN
4                                                    NaN
..                                                   …
786                                                  NaN
787                                                  NaN
788                                                  NaN
789                                                  NaN
790                                                  NaN

                                           Timing_PMG_VF  \
0    Timing based ventral or cephalic furrow for al…
1                                                    NaN
2                                                    NaN
3                                                    NaN
4                                                    NaN
..                                                   …
786                                                  NaN
787                                                  NaN
788                                                  NaN
789                                                  NaN
790                                                  NaN

                                                Filename  \
0    name of .tif file with image data. Note: some …
1      202011021800_cylinder2_max_rot_scaled_view1.tif
2      202011031830_cylinder2_max_rot_scaled_view1.tif
3      202011071345_cylinder2_max_rot_scaled_view1.tif
4                          202011301820_max_l14-23.tif
..                                                   …
786          MAX_Cyl1_2_1_c000001_rot_scaled_view1.tif
787          MAX_Cyl1_2_1_c000001_rot_scaled_view1.tif
788          MAX_Cyl1_2_1_c000001_rot_scaled_view1.tif
789          MAX_Cyl1_2_1_c000001_rot_scaled_view1.tif
```

```
790         MAX_Cyl1_2_1_c000001_rot_scaled_view1.tif

        Time_Resolution_Seconds                                  Notes
0    Time resolution in seconds  Any additional notes about the dataset
1                           NaN      fluorofore chromosome: CyoSqhGFP\n
2                           NaN      fluorofore chromosome: CyoSqhGFP\n
3                           NaN      fluorofore chromosome: CyoSqhGFP\n
4                           NaN      fluorofore chromosome: CyoSqhGFP\n
..                          …                                       …
786                         NaN                                     NaN
787                         NaN                                     NaN
788                         NaN                                     NaN
789                         NaN                                     NaN
790                         NaN                                     NaN

[791 rows x 13 columns]
```

As you can see, each row corresponds to one recording. The first row explains the meaning of each column:

[118]:
```
atlas_database.loc[0, "Embryo_ID"] # look at row 0, column "Embryo_ID" to see␣
↪what it this row means
```

[118]:
```
'ID of embryo (time of recording), in YYYYMMDDHHMM format'
```

1.1 Multichannel recordings

Note that for fixed datasets, a multi-channel image (e.g. for Fushi-Tarazu *and* Runt) will be listed as two recordings, with the same `Embryo_ID`.

[119]:
```
# let's remove the first row to get the data only

atlas_database_data = atlas_database.iloc[1:].reset_index(drop=True)
```

2 Examples

We now show some examples of querying the atlas and loading some of the resulting data.

[120]:
```
# let's list all available genotypes

atlas_database_data["Genotype"].unique()
```

[120]:
```
array(['67-15_1to3copies_UAS-Even-Skipped',
       '67-singlecopy_15-singlecopy_UAS-18W-HA',
       '67-singlecopy_15-singlecopy_UAS-Even-Skipped',
       '67-singlecopy_15-singlecopy_UAS-Runt',
       '67-singlecopy_15-singlecopy_UAS-Tollo-HA',
```

```
         '67-singlecopy_UAS-Even-Skipped', '67-singlecopy_UAS-Runt',
         'Halo_Hetero_even-skipped[r13]_Hetero',
         'Halo_Hetero_snail[IIG05]_Hetero',
         'Halo_Hetero_twist[ey53]_Hetero', 'Halo_snail[IIG05]',
         'Halo_twist[ey53]', 'TrafficJam-Gal4_UAS-Fat2RNAi',
         'UAS-Even-Skipped', 'WT', 'WT_17_Degrees', 'bicoid[E1]_nanos[BN]',
         'bicoid[E1]_nanos[BN]_tsl[4]', 'concertina-t48',
         'even-skipped[r13]', 'spaetzle[A]', 'sqh[Ax3]', 'toll[RM9]'],
        dtype=object)
```

[121]:
```python
# ... or tagged proteins
atlas_database_data["Fluorophore"].unique()
```

[121]:
```
array(['Sqh-GFP', 'Bazooka-GFP', 'CAAX-mCherry', 'Runt', 'Bazooka',
       'ECad-GFP', 'Even_Skipped', 'Even_Skipped-YFP', 'Fushi_Tarazu',
       'H2a-mCherry_Klarsicht', 'Hairy', 'Moesin-GFP', 'Neurotactin',
       'Paired', 'Shotgun-GFP', 'Sloppy_Paired', 'Sqh-mCherry', 'Tartan',
       'Toll_6', 'Toll_8', 'endogenous_ECad-GFP', 'histone-RFP',
       'ubiquitous-Rock-GFP', 'utr-mCherry', 'Histone-2B-RFP', 'ECad',
       'CAAX-mCherry_Sqh-GFP'], dtype=object)
```

[122]:
```python
# let's find all fixed WT datasets which have been stained for Runt

filtered_data = atlas_database_data[(atlas_database_data['Genotype'] == 'WT')
                                    & (atlas_database_data['Is_Live'] ==␣
 ↪'False')
                                    & (atlas_database_data['Fluorophore'] ==␣
 ↪'Runt')]
```

[123]:
```python
filtered_data
```

[123]:
```
        Embryo_ID Genotype Fluorophore Is_Live Has_PIV Has_PIVLab Timing_PIV  \
215  201904121131       WT        Runt   False   False      False        NaN
216  201904121153       WT        Runt   False   False      False        NaN
217  201904121159       WT        Runt   False   False      False        NaN
218  201904121410       WT        Runt   False   False      False        NaN
219  201904121419       WT        Runt   False   False      False        NaN
..            …      …          …      …     …       …        …
341  202010231850       WT        Runt   False   False      False        NaN
342  202010231910       WT        Runt   False   False      False        NaN
343  202101252010       WT        Runt   False   False      False        NaN
344  202101252030       WT        Runt   False   False      False        NaN
345  202101252035       WT        Runt   False   False      False        NaN

    Timing_Runt Timing_Warped Timing_PMG_VF  \
215        20.6           NaN           NaN
216       34.81           NaN           NaN
```

```
217      28.061           NaN           NaN
218      35.486           NaN           NaN
219      39.054           NaN           NaN
..          …           …           …
341         NaN           NaN           NaN
342         NaN           NaN           NaN
343         NaN           NaN           NaN
344         NaN           NaN           NaN
345         NaN           NaN           NaN

                                      Filename Time_Resolution_Seconds  \
215  MAX_Cyl1_2_000000_c1_rot_scaled_view1.tif                      NaN
216  MAX_Cyl1_2_000000_c1_rot_scaled_view1.tif                      NaN
217  MAX_Cyl1_2_000000_c1_rot_scaled_view1.tif                      NaN
218  MAX_Cyl1_2_000000_c1_rot_scaled_view1.tif                      NaN
219  MAX_Cyl1_2_000000_c1_rot_scaled_view1.tif                      NaN
..                                          …                      …
341  MAX_Cyl1_2_000000_c1_rot_scaled_view1.tif                      NaN
342  MAX_Cyl1_2_000000_c1_rot_scaled_view1.tif                      NaN
343  MAX_Cyl1_2_000000_c1_rot_scaled_view1.tif                      NaN
344  MAX_Cyl1_2_000000_c1_rot_scaled_view1.tif                      NaN
345  MAX_Cyl1_2_000000_c1_rot_scaled_view1.tif                      NaN

                                            Notes
215  note: for analysis of runt, consider using the…
216  note: for analysis of runt, consider using the…
217  note: for analysis of runt, consider using the…
218  note: for analysis of runt, consider using the…
219  note: for analysis of runt, consider using the…
..                                              …
341  note: for analysis of runt, consider using the…
342  note: for analysis of runt, consider using the…
343  note: for analysis of runt, consider using the…
344  note: for analysis of runt, consider using the…
345  note: for analysis of runt, consider using the…

[125 rows x 13 columns]
```

2.1 A note on timing data

Depending on the data available for the recording, different timing information is available, based on the shape of Runt stripes (for fixed WT data), the tissue flow field (for live data), or the morphogenetic events of ventral furrow and cephalic furrow formation (for time alignment across different genotypes).

Note that the timing information is a single time stamp, corresponding to the timing of the first frame of the movie. For the fast-marching based "warped" time alignment (which assigns a different morphological time stamp to each frame), please use the full MATLAB based morpho-dynamic atlas

code base, available here: https://github.com/npmitchell/dynamicAtlas.

[124]:
```
# assemble a pseudo-time line from fixed images of the pair-rule gene␣
↪Fushi-Taratzu

fushi_data = atlas_database_data[(atlas_database_data['Genotype'] == 'WT')
                                 & (atlas_database_data['Is_Live'] == 'False')
                                 & (atlas_database_data['Fluorophore'] ==␣
↪'Fushi_Tarazu')]

# subselect columns ID, timing, and file name, then sort by time (making sure␣
↪the computer knows the timestamps are numbers)

fushi_data = fushi_data[['Embryo_ID', 'Timing_Runt', 'Genotype', 'Fluorophore',␣
↪'Filename']]
fushi_data['Timing_Runt'] = pd.to_numeric(fushi_data['Timing_Runt'],␣
↪errors='coerce')
fushi_data = fushi_data.sort_values(by='Timing_Runt', ascending=True)

fushi_data
```

[124]:
```
        Embryo_ID  Timing_Runt Genotype   Fluorophore  \
120  201905091624       1.0000       WT  Fushi_Tarazu
117  201905091543       2.5269       WT  Fushi_Tarazu
115  201905091402       6.4909       WT  Fushi_Tarazu
111  201905071651       6.6360       WT  Fushi_Tarazu
122  201905091640      16.1040       WT  Fushi_Tarazu
118  201905091557      17.1710       WT  Fushi_Tarazu
127  201905101422      20.8520       WT  Fushi_Tarazu
129  201905101440      22.1000       WT  Fushi_Tarazu
125  201905091719      22.8860       WT  Fushi_Tarazu
119  201905091604      24.9940       WT  Fushi_Tarazu
124  201905091714      27.9600       WT  Fushi_Tarazu
112  201905071659      31.8950       WT  Fushi_Tarazu
116  201905091409      32.0700       WT  Fushi_Tarazu
121  201905091633      33.3190       WT  Fushi_Tarazu
113  201905071718      34.6970       WT  Fushi_Tarazu
123  201905091648      34.9790       WT  Fushi_Tarazu
128  201905101432      36.3300       WT  Fushi_Tarazu
126  201905091724      38.8090       WT  Fushi_Tarazu
114  201905091349      47.3730       WT  Fushi_Tarazu

                                    Filename
120  MAX_Cyl1_2_000000_c1_rot_scaled_view1.tif
117  MAX_Cyl1_2_000000_c1_rot_scaled_view1.tif
115  MAX_Cyl1_2_000000_c1_rot_scaled_view1.tif
111  MAX_Cyl1_2_000000_c1_rot_scaled_view1.tif
```

```
122  MAX_Cyl1_2_000000_c1_rot_scaled_view1.tif
118  MAX_Cyl1_2_000000_c1_rot_scaled_view1.tif
127  MAX_Cyl1_2_000000_c1_rot_scaled_view1.tif
129  MAX_Cyl1_2_000000_c1_rot_scaled_view1.tif
125  MAX_Cyl1_2_000000_c1_rot_scaled_view1.tif
119  MAX_Cyl1_2_000000_c1_rot_scaled_view1.tif
124  MAX_Cyl1_2_000000_c1_rot_scaled_view1.tif
112  MAX_Cyl1_2_000000_c1_rot_scaled_view1.tif
116  MAX_Cyl1_2_000000_c1_rot_scaled_view1.tif
121  MAX_Cyl1_2_000000_c1_rot_scaled_view1.tif
113  MAX_Cyl1_2_000000_c1_rot_scaled_view1.tif
123  MAX_Cyl1_2_000000_c1_rot_scaled_view1.tif
128  MAX_Cyl1_2_000000_c1_rot_scaled_view1.tif
126  MAX_Cyl1_2_000000_c1_rot_scaled_view1.tif
114  MAX_Cyl1_2_000000_c1_rot_scaled_view1.tif
```

3 Loading image data

Let's load some of these `.tif` files. To do this, you first need to download the data from the Dryad repository. There are stored there as compressed archive files (like `.zip`'s), one for each genotype - except for WT, which is broken up further into multiple archive files because of it's large size.

Let's download the `WT_Fushi_Tarazu.tar.lz4` archive. On a unix-like system you can unpack it in the terminal via

`~$ lz4 -d folder_name.tar.lz4 -c | tar xvf -`

After unpacking the `WT_Fushi_Tarazu` folder, please restructure the directory structure as follows: 1. Create a `WT` directory 2. Move `WT_Fushi_Tarazu` in the `WT` directory 3. Rename `WT_Fushi_Tarazu` to `Fushi_Tarazu` This "undoes" the breaking-up of the WT genotype folder into multiple archives and will make it easier to load the files. You can of cause automate this step if you want to.

Next, we will set the folder ("path") where all the data is located. `Path.cwd()` will set it to the same folder that this jupyter notebook is located. You can also specify another folder, e.g. `Path(r"C:\Users\user\morphodynamic_atlas_data")`.

[65]:
```
base_path = Path.cwd()
```

[66]:
```
# let's print all the sub-directories in the base path to make sure we are in
↪the right place.
[f for f in base_path.iterdir() if f.is_dir()]
```

[66]:
```
[PosixPath('/mnt/data/flydrive_clone/clean copy of
Atlas_Data/Atlas_Data/67-15_1to3copies_UAS-Even-Skipped'),
 PosixPath('/mnt/data/flydrive_clone/clean copy of
Atlas_Data/Atlas_Data/WT_17_Degrees'),
 PosixPath('/mnt/data/flydrive_clone/clean copy of
```

```
Atlas_Data/Atlas_Data/67-singlecopy_15-singlecopy_UAS-18W-HA'),
 PosixPath('/mnt/data/flydrive_clone/clean copy of
Atlas_Data/Atlas_Data/67-singlecopy_15-singlecopy_UAS-Even-Skipped'),
 PosixPath('/mnt/data/flydrive_clone/clean copy of
Atlas_Data/Atlas_Data/67-singlecopy_15-singlecopy_UAS-Runt'),
 PosixPath('/mnt/data/flydrive_clone/clean copy of
Atlas_Data/Atlas_Data/Halo_Hetero_even-skipped[r13]_Hetero'),
 PosixPath('/mnt/data/flydrive_clone/clean copy of
Atlas_Data/Atlas_Data/Halo_Hetero_snail[IIG05]_Hetero'),
 PosixPath('/mnt/data/flydrive_clone/clean copy of
Atlas_Data/Atlas_Data/67-singlecopy_UAS-Even-Skipped'),
 PosixPath('/mnt/data/flydrive_clone/clean copy of
Atlas_Data/Atlas_Data/Halo_Hetero_twist[ey53]_Hetero'),
 PosixPath('/mnt/data/flydrive_clone/clean copy of
Atlas_Data/Atlas_Data/WT_27_Degrees'),
 PosixPath('/mnt/data/flydrive_clone/clean copy of
Atlas_Data/Atlas_Data/bicoid[E1]_nanos[BN]'),
 PosixPath('/mnt/data/flydrive_clone/clean copy of
Atlas_Data/Atlas_Data/toll[RM9]'),
 PosixPath('/mnt/data/flydrive_clone/clean copy of
Atlas_Data/Atlas_Data/Halo_snail[IIG05]'),
 PosixPath('/mnt/data/flydrive_clone/clean copy of
Atlas_Data/Atlas_Data/67-singlecopy_UAS-Runt'),
 PosixPath('/mnt/data/flydrive_clone/clean copy of
Atlas_Data/Atlas_Data/sqh[Ax3]'),
 PosixPath('/mnt/data/flydrive_clone/clean copy of Atlas_Data/Atlas_Data/UAS-
Even-Skipped'),
 PosixPath('/mnt/data/flydrive_clone/clean copy of
Atlas_Data/Atlas_Data/TrafficJam-Gal4_UAS-Fat2RNAi'),
 PosixPath('/mnt/data/flydrive_clone/clean copy of
Atlas_Data/Atlas_Data/Halo_twist[ey53]'),
 PosixPath('/mnt/data/flydrive_clone/clean copy of
Atlas_Data/Atlas_Data/.ipynb_checkpoints'),
 PosixPath('/mnt/data/flydrive_clone/clean copy of
Atlas_Data/Atlas_Data/[INTERNAL] PIVlab_results'),
 PosixPath('/mnt/data/flydrive_clone/clean copy of
Atlas_Data/Atlas_Data/spaetzle[A]'),
 PosixPath('/mnt/data/flydrive_clone/clean copy of
Atlas_Data/Atlas_Data/67-singlecopy_15-singlecopy_UAS-Tollo-HA'),
 PosixPath('/mnt/data/flydrive_clone/clean copy of Atlas_Data/Atlas_Data/even-
skipped[r13]'),
 PosixPath('/mnt/data/flydrive_clone/clean copy of Atlas_Data/Atlas_Data/WT'),
 PosixPath('/mnt/data/flydrive_clone/clean copy of
Atlas_Data/Atlas_Data/[INTERNAL] timing'),
 PosixPath('/mnt/data/flydrive_clone/clean copy of
Atlas_Data/Atlas_Data/bicoid[E1]_nanos[BN]_tsl[4]'),
 PosixPath('/mnt/data/flydrive_clone/clean copy of
```

```
Atlas_Data/Atlas_Data/concertina-t48')]
```

[104]:
```
# now, let's load the Fushi-Tarazu data. to do so, we iterate over the rows in␣
↪our filtered dataset
# we the load the data and add it to our table

fushi_data['Image'] = object # initialize the new row

for index, row in fushi_data.iterrows():
    file_path = base_path.joinpath(row["Genotype"], row["Fluorophore"],␣
↪row["Embryo_ID"], row["Filename"])
    image = plt.imread(file_path)
    fushi_data.at[index, "Image"] = image
```

[113]:
```
# let's look at the image data for a specific embryo

selected_image = fushi_data[fushi_data["Embryo_ID"] == '201905091624']["Image"].
↪values[0]

plt.imshow(selected_image)
```

[113]: <matplotlib.image.AxesImage at 0x7feca3339ed0>

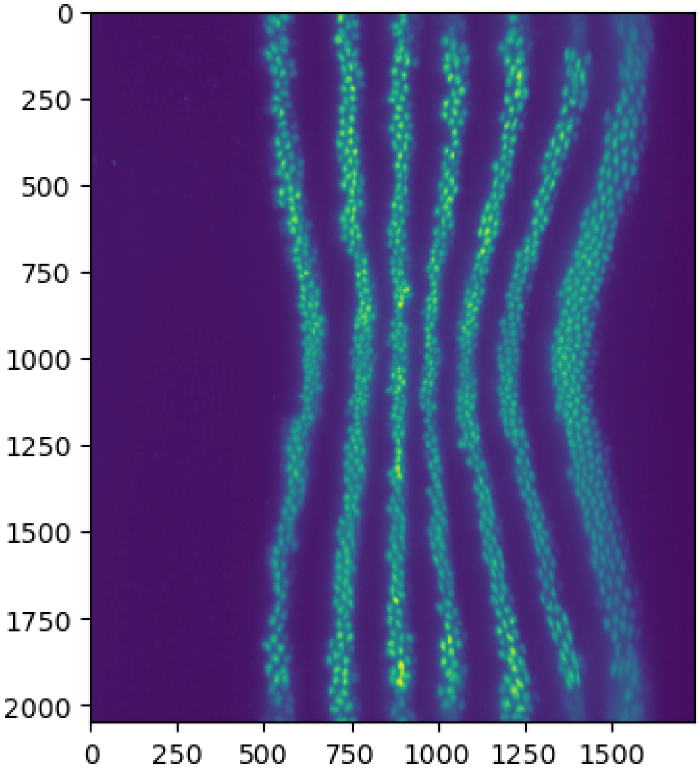

4 Processing

You can now process the image(s) in whatever way you want: smooth them, compute gradients, correlate images across samples or genotypes, ...

We will leave this up to you.

5 Loading PIV data

For a subset of live recordings, we provide PIV data (tissue flow fields - please see the manuscript for a detailed explanation). There are two sets of PIV data whose presence is indicated by the `Has_PIV` and `Has_PIVLab` columns. They were computed with slightly different methods and saved in slightly different formats. `Has_PIV`-data is saved in a `PIV/` subfolder, with one file per timepoint and was computed using a custom script optimized for speed. `Has_PIVLab`-data was computed with the MATLAB add-on PIV Lab, optimized for precision, and is saved as a single file per dataset. In both cases, the files are located in the same folder as all other data on the recording.

Since the PIV was created by MATLAB, they are in the `.mat` format. We can however read this format in Python without issue. Let's look at one example.

[125]:
```
# list all recordings where PIVlab data is available
atlas_database_data[(atlas_database_data['Has_PIVLab'] == 'True')]
```

[125]:
```
        Embryo_ID                                 Genotype  Fluorophore  \
4    202011271230  67-singlecopy_15-singlecopy_UAS-Even-Skipped      Sqh-GFP
5    202011271400  67-singlecopy_15-singlecopy_UAS-Even-Skipped      Sqh-GFP
6    202011271520  67-singlecopy_15-singlecopy_UAS-Even-Skipped      Sqh-GFP
25   202007011145                Halo_Hetero_twist[ey53]_Hetero      Sqh-GFP
26   202007081130                Halo_Hetero_twist[ey53]_Hetero      Sqh-GFP
27   202007091200                Halo_Hetero_twist[ey53]_Hetero      Sqh-GFP
47   202101201045                             Halo_snail[IIG05]      Sqh-GFP
54   202101221425                             Halo_snail[IIG05]      Sqh-GFP
60   202101281300                             Halo_snail[IIG05]      Sqh-GFP
61   202007171100                              Halo_twist[ey53]      Sqh-GFP
62   202007171400                              Halo_twist[ey53]      Sqh-GFP
64   202007301030                              Halo_twist[ey53]      Sqh-GFP
374  202001101100                                            WT  Sqh-mCherry
376  202001120940                                            WT  Sqh-mCherry
379  202001252040                                            WT  Sqh-mCherry
464  202201311320                                 WT_17_Degrees  Sqh-mCherry
465  202201311600                                 WT_17_Degrees  Sqh-mCherry
466  202202011630                                 WT_17_Degrees  Sqh-mCherry
467  202201311320                                 WT_17_Degrees  Sqh-mCherry
468  202201311600                                 WT_17_Degrees  Sqh-mCherry
469  202202011630                                 WT_17_Degrees  Sqh-mCherry
530  202001151953                             even-skipped[r13]      Sqh-GFP
```

532	202001171624	even-skipped[r13]	Sqh-GFP
533	202001181115	even-skipped[r13]	Sqh-GFP
554	201712191340	spaetzle[A]	Sqh-GFP
556	201712201630	spaetzle[A]	Sqh-GFP
557	201712201930	spaetzle[A]	Sqh-GFP
755	202101111920	toll[RM9]	Sqh-GFP
756	202101121810	toll[RM9]	Sqh-GFP
757	202101191940	toll[RM9]	Sqh-GFP

	Is_Live	Has_PIV	Has_PIVLab	Timing_PIV	Timing_Runt	Timing_Warped	\
4	True	False	True	NaN	NaN	NaN	
5	True	False	True	NaN	NaN	NaN	
6	True	False	True	NaN	NaN	NaN	
25	True	False	True	22	NaN	NaN	
26	True	False	True	17	NaN	NaN	
27	True	False	True	23	NaN	NaN	
47	True	False	True	NaN	NaN	NaN	
54	True	False	True	NaN	NaN	NaN	
60	True	False	True	NaN	NaN	NaN	
61	True	False	True	NaN	NaN	NaN	
62	True	False	True	NaN	NaN	NaN	
64	True	False	True	NaN	NaN	NaN	
374	True	True	True	18	NaN	NaN	
376	True	True	True	8	NaN	NaN	
379	True	False	True	14	NaN	NaN	
464	True	False	True	36	NaN	NaN	
465	True	False	True	NaN	NaN	NaN	
466	True	False	True	18	NaN	NaN	
467	True	False	True	36	NaN	NaN	
468	True	False	True	NaN	NaN	NaN	
469	True	False	True	18	NaN	NaN	
530	True	False	True	NaN	NaN	NaN	
532	True	False	True	NaN	NaN	NaN	
533	True	False	True	NaN	NaN	NaN	
554	True	False	True	NaN	NaN	NaN	
556	True	False	True	NaN	NaN	NaN	
557	True	False	True	NaN	NaN	NaN	
755	True	False	True	NaN	NaN	NaN	
756	True	False	True	NaN	NaN	NaN	
757	True	False	True	NaN	NaN	NaN	

	Timing_PMG_VF	Filename	\
4	7	cylinder1_max.tif	
5	15	cylinder1_max.tif	
6	1	cylinder1_max.tif	
25	0	MAX_Cyl1_2_000000_c1_rot_scaled_view1.tif	
26	5	orig_MAX_Cyl1_2_000000_c1_rot_scaled_view1.tif	

27	-1	MAX_Cyl1_2_000000_c1_rot_scaled_view1.tif
47	8	202101201045_SnailMinus_l8-13.tif
54	8	202101221425_SnailMinus_l5-10.tif
60	4	202101281300_SnailMinus_l7-12.tif
61	-1	202007171100_twistMInus_l6-11.tif
62	-10	202007171400_twistMinus_l6-11.tif
64	-2	202007301030_twistMinus_l6-11.tif
374	4	MAX_Cyl1_2_000000_c1_rot_scaled_view1.tif
376	12	MAX_Cyl1_2_000000_c1_rot_scaled_view1.tif
379	8	MAX_Cyl1_2_000000_c1_rot_scaled_view1.tif
464	-8	MAX_Cyl1_2_000000_c0_rot_scaled_view1.tif
465	0	cylinder1_max_6to14.tif
466	4	MAX_Cyl1_2_000000_c0_rot_scaled_view1.tif
467	-8	MAX_Cyl1_2_000000_c0_rot_scaled_view1.tif
468	0	cylinder1_max_6to14.tif
469	4	MAX_Cyl1_2_000000_c0_rot_scaled_view1.tif
530	11	NaN
532	-1	NaN
533	11	NaN
554	-9	cylinder1_max.tif
556	-11	cylinder1_max.tif
557	-3	cylinder1_max.tif
755	-6	cylinder1_c1_max.tif
756	-8	cylinder1_max.tif
757	-8	cylinder1_c1_max.tif

	Time_Resolution_Seconds	Notes
4	NaN	fluorofore chromosome: CyoSqhGFP\n
5	NaN	fluorofore chromosome: CyoSqhGFP\n
6	NaN	fluorofore chromosome: CyoSqhGFP\n
25	NaN	fluorofore chromosome: CyoSqhGFP\n
26	NaN	fluorofore chromosome: CyoSqhGFP\n
27	NaN	fluorofore chromosome: CyoSqhGFP\n
47	NaN	fluorofore chromosome: CyoSqhGFP\n\nImaged by …
54	NaN	fluorofore chromosome: CyoSqhGFP\n\nImaged by …
60	NaN	fluorofore chromosome: CyoSqhGFP\n\nImaged by …
61	NaN	fluorofore chromosome: CyoSqhGFP\n
62	NaN	fluorofore chromosome: CyoSqhGFP\n
64	NaN	fluorofore chromosome: CyoSqhGFP\n
374	NaN	NaN
376	NaN	NaN
379	NaN	NaN
464	NaN	NaN
465	NaN	NaN
466	NaN	NaN
467	NaN	NaN
468	NaN	NaN

```
469                      NaN                                        NaN
530                      NaN           fluorofore chromosome: cyoSqhGFP\n
532                      NaN           fluorofore chromosome: cyoSqhGFP\n
533                      NaN           fluorofore chromosome: cyoSqhGFP\n
554                      NaN                                        NaN
556                      NaN                                        NaN
557                      NaN                                        NaN
755                      NaN                                        NaN
756                      NaN                                        NaN
757                      NaN                                        NaN
```

[154]:
```python
# let's pick one of the WT recordings

selected_embryo = atlas_database_data.iloc[376]
selected_embryo
```

[154]:
```
Embryo_ID                                          202001120940
Genotype                                                     WT
Fluorophore                                        Sqh-mCherry
Is_Live                                                    True
Has_PIV                                                    True
Has_PIVLab                                                 True
Timing_PIV                                                    8
Timing_Runt                                                 NaN
Timing_Warped                                               NaN
Timing_PMG_VF                                                12
Filename                 MAX_Cyl1_2_000000_c1_rot_scaled_view1.tif
Time_Resolution_Seconds                                     NaN
Notes                                                       NaN
Name: 376, dtype: object
```

[155]:
```python
# let's list all the files we have for this recording
selected_embryo_path = base_path.joinpath(selected_embryo["Genotype"],␣
 ↪selected_embryo["Fluorophore"], selected_embryo["Embryo_ID"])

sorted(selected_embryo_path.iterdir())
```

[155]:
```
[PosixPath('/mnt/data/flydrive_clone/clean copy of Atlas_Data/Atlas_Data/WT/Sqh-
mCherry/202001120940/202102181445_time.txt'),
 PosixPath('/mnt/data/flydrive_clone/clean copy of Atlas_Data/Atlas_Data/WT/Sqh-
mCherry/202001120940/MAX_Cyl1_2_000000_c1_rot_scaled_view1.tif'),
 PosixPath('/mnt/data/flydrive_clone/clean copy of Atlas_Data/Atlas_Data/WT/Sqh-
mCherry/202001120940/MEAN_Cyl1_2_000000_c1_rot_scaled_view1.tif'),
 PosixPath('/mnt/data/flydrive_clone/clean copy of Atlas_Data/Atlas_Data/WT/Sqh-
mCherry/202001120940/PIV'),
 PosixPath('/mnt/data/flydrive_clone/clean copy of Atlas_Data/Atlas_Data/WT/Sqh-
mCherry/202001120940/PIV_filtered'),
```

```
 PosixPath('/mnt/data/flydrive_clone/clean copy of Atlas_Data/Atlas_Data/WT/Sqh-
mCherry/202001120940/WT_202001120940_PIVlab.mat'),
 PosixPath('/mnt/data/flydrive_clone/clean copy of Atlas_Data/Atlas_Data/WT/Sqh-
mCherry/202001120940/cephallicFurrowOnset.txt'),
 PosixPath('/mnt/data/flydrive_clone/clean copy of Atlas_Data/Atlas_Data/WT/Sqh-
mCherry/202001120940/t0V.txt'),
 PosixPath('/mnt/data/flydrive_clone/clean copy of Atlas_Data/Atlas_Data/WT/Sqh-
mCherry/202001120940/timematch_Runt_cephallicFurrowOnset.txt'),
 PosixPath('/mnt/data/flydrive_clone/clean copy of Atlas_Data/Atlas_Data/WT/Sqh-
mCherry/202001120940/timematch_linearOffset_curlSign.txt'),
 PosixPath('/mnt/data/flydrive_clone/clean copy of Atlas_Data/Atlas_Data/WT/Sqh-
mCherry/202001120940/timematch_linearOffset_vmagAlign.txt')]
```

The file we need is `[...]_PIVlab.mat`. Let's load it. The result of loading a `.mat` file is a dictionary comprising the x and y components of the PIV field, and the grid on which they are defined. Please see the PIV lab website for detailed documentation.

[160]:
```
mat_file_path = [f for f in selected_embryo_path.iterdir() if str(f).endswith(".
 ↪mat")][0]
mat_file = io.loadmat(mat_file_path)

mat_file.keys()
```

[160]:
```
dict_keys(['__header__', '__version__', '__globals__', 'uu_lst', 'vv_lst',
'u_filt_lst', 'v_filt_lst', 'xx', 'yy'])
```

[161]:
```
# u_filt_lst and v_filt_lst contain the arrays of x and y components of the␣
 ↪(filtered) PIV field across the timepoints of the movie
# (here, 39).

mat_file["uu_lst"].shape
```

[161]:
```
(89, 151, 107)
```

[162]:
```
# let us visualize the flow field at one time oint using a stream plot

timepoint = 30

plt.streamplot(mat_file["xx"], mat_file["yy"], mat_file["u_filt_lst"][30],␣
 ↪mat_file["v_filt_lst"][30])
plt.axis("equal")
```

[162]:
```
(21.0, 1717.0, 21.0, 2421.0)
```

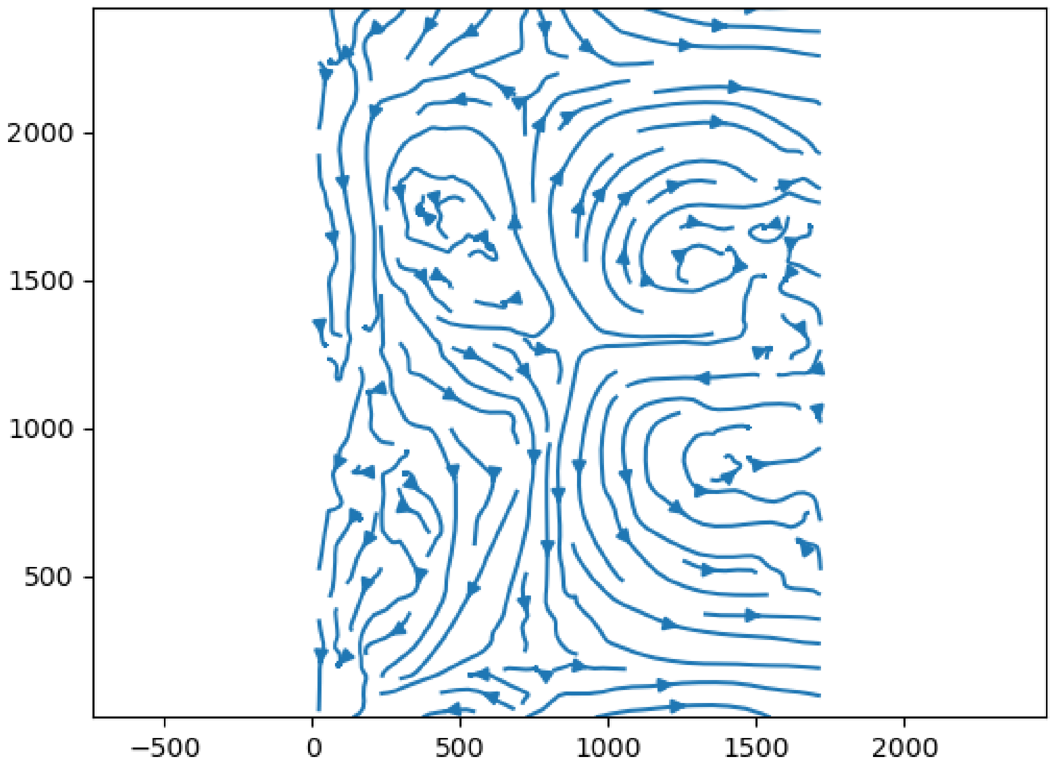

2000
1500
1000
500
−500
0
500
1000
1500
2000

DynamicAtlas tutorial for MATLAB-based interface: atlas assembly, timeline creation and fixed-data timestamping

Below follows a tutorial for users who wish to use the atlas methods on a new ensemble of data – whether by using a different set of data in the atlas, or by adding their own data to the atlas. We developed this more intensive code in MATLAB. Users who wish only to query the atlas and perform analysis of data of choice can do so more easily on our Python interface, for which we also have created a separate tutorial.

Example screenshots will be shown from running the code on a 2023 MacBook Pro (Apple M3 Pro chip) with 18 GB RAM and 512 GB storage, running MacOS Sequoia 15.1. However, the code is platform-independent, and the same procedure also works on the Windows or Linux operating systems (tested on Windows 10 and Ubuntu 18.04.6 LTS). The code should take on the order of 45 minutes to run on the demo dataset, depending on machine capabilities.

—

Table of Contents:

Required Specs and Download Instructions 39
Demo code walkthrough: Data Conventions 44
Demo code walkthrough: Atlas Assembly 48
Demo code walkthrough: Timeline Creation 58
Demo code walkthrough: Fixed-data Timestamping 70

—

Required Specs and Download Instructions

Below follows information about the required specifications for running the code, and information for the required downloads: MATLAB, demo data, and DynamicAtlas code.

Required Specs:

Any major operating system: Windows, MacOS, Linux
30 GB hard drive space (~25 GB for MATLAB, <5 GB for data & code download)
18 GB RAM or more (may still run with less, but subject to stalling or slowing)
Recent MATLAB version, from R2020a onwards (shown below on R2024a)
Tool to unzip compressed files (like 7Zip on Windows, ArchiveUtility on MacOS, or ArchiveManager on Linux)

Downloading MATLAB:

Follow the instructions listed here to download the MATLAB application:
https://www.mathworks.com/help/install/ug/install-products-with-internet-connection.html

During the installation process, a screen will appear inquiring which toolboxes to download. Check "select all" on this screen (shown below), as some functions used by DynamicAtlas are defined in the toolboxes.

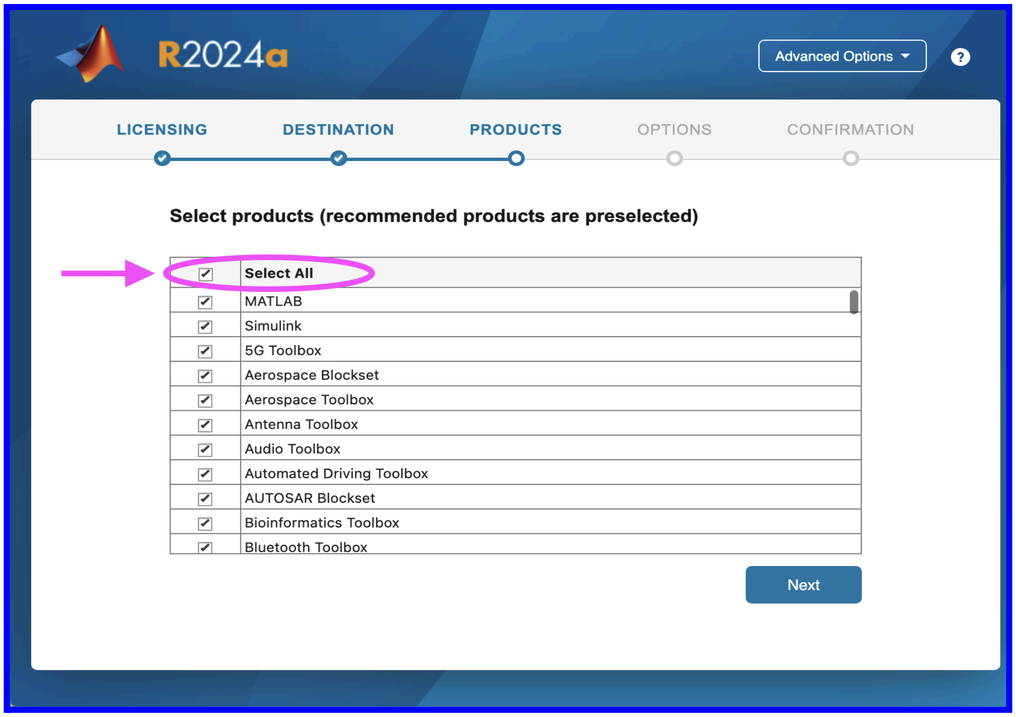

NOTE: A pre-installed version of Matlab may be used, but it is possible a function may not be recognized due to a toolbox not being previously downloaded. This may yield an error similar to the following (here, function ‘pdist2’ is missing):

```
Undefined function 'pdist2' for input arguments of type 'double'.

Error in dynamicAtlas.dynamicAtlas/makeMasterTimeLineRealspace (line 1231)
                        d1 = pdist2(cI, cJ);

Error in dynamicAtlas.dynamicAtlas/makeMasterTimeline (line 200)
                da.makeMasterTimeLineRealspace(genotype, label, Options)

Error in demo_dynamicAtlas_functionality (line 127)
da.makeMasterTimeline('WT','Runt', Options)
```

This can be fixed by looking up the toolbox containing this function, and downloading it, see instructions for downloading and adding new toolboxes here: (https://www.mathworks.com/help/install/ug/add-to-existing-installation.html).

To look up the toolbox of a given function, one method is to use the ‘doc’ command to access the function documentation, as below for ‘pdist2’:

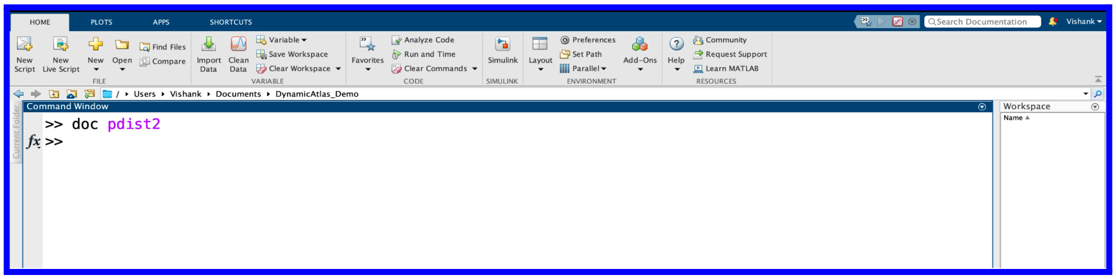

which will open the function’s documentation webpage. The toolbox containing the function can be found at the upper left corner of the documentation page, as shown (here, the Statistics and Machine Learning Toolbox contains ‘pdist2’):

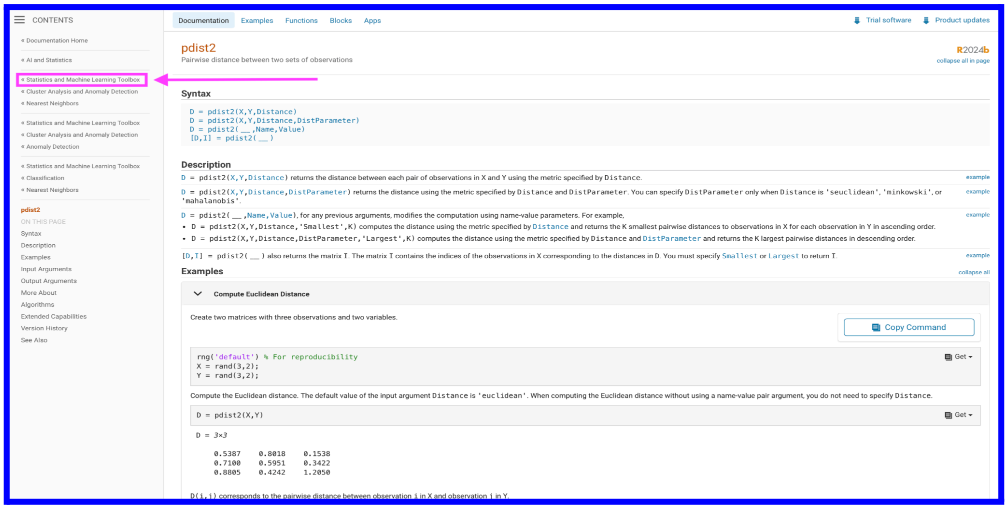

Downloading Demo Data:

We have uploaded our Atlas data to a Dryad repository (https://datadryad.org/stash/dataset/doi:10.25349/D9WW43 — downloads work best on the Firefox or Google Chrome browsers).

For this tutorial, however, we have assembled a small Demo dataset with minimal inputs. This demo dataset, which contains five Runt-labeled samples from the Atlas, can be accessed at its own link (hosted as a Zenodo data repository):

https://doi.org/10.5281/zenodo.14792464

with the demo file (~1 GB) called

DEMO_DATASET.tar.lz4

Download this dataset to a directory of choice. This data is in a compressed file format, and can be extracted with an archive utility. The extraction can also be done through a command line (Terminal on MacOS/Linux, Command Prompt on Windows), after changing to the directory where the file is stored using the command cd (e.g. cd /Users/Vishank/Desktop/DynamicAtlas_Demo). On MacOS or Linux, the data can then be extracted with e.g. the command…

lz4 -d DEMO_DATASET.tar.lz4 -c | tar xvf -

…as demonstrated below, before and after executing the command (on MacOS):

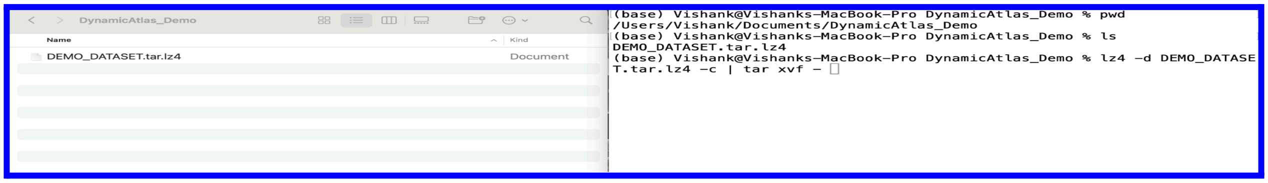

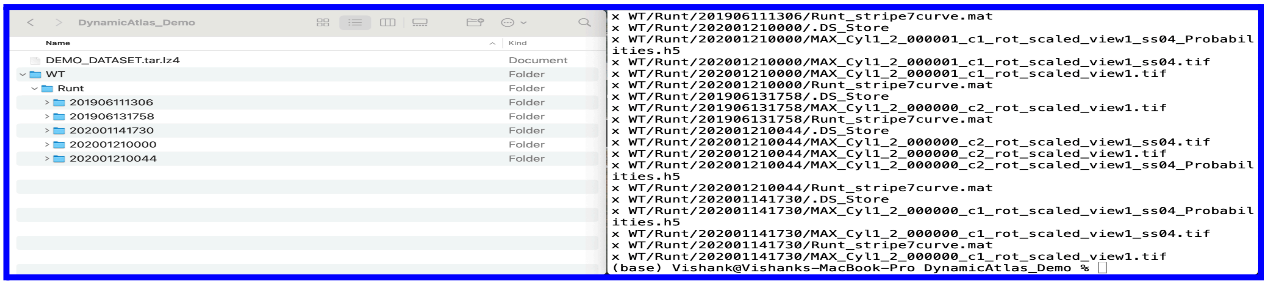

(Note pwd lists the working directory, and ls lists the files.) The data will then be extracted to a folder structure. The above extraction works on Linux similarly. On Windows, while Command Prompt can be used, the popular archive utility 7Zip can be used to perform this extraction more easily. Troubleshooting for this step follows below:

—

If the lz4 command is not available on MacOS, recent OS versions can extract the file using the tar command, for example: tar -lzip -xvf DEMO_DATASET.tar.lz4. Otherwise, the lz4 command can be installed with the popular Homebrew utility, see link: https://formulae.brew.sh/formula/lz4

On Linux, if the lz4 command is unavailable, it can be installed using commands shown at the following link: https://www.tecmint.com/install-lz4-linux/

On Windows, if 7Zip doesn't work for the extraction directly (a possibility), one can follow the method described here to extract by using 7Zip in a different way: https://superuser.com/questions/1292802/how-to-decompress-lz4
This will convert the .tar.lz4 to a .tar using lz4.exe (downloadable from https://sourceforge.net/projects/lz4.mirror/files/ — this .exe may need to be moved into the data directory), and 7Zip will then work as normal on this.tar file, as will the Command Prompt command 'tar'.

Extraction may also create extraneous files such as '.DS_STORE' and '_MACOSX' — delete all such files, as their presence may cause errors.

—

For our code, we use the following directory convention:

FOLDER CONVENTION: The data must be organized in folders as

Genotype > Marker > Dataset ID

For example, WT > Runt > 201906111306. If folders are not organized with this convention, errors may occur when the function buildLookupMap.m is called to construct the Atlas object. Depending on the file downloaded and the extraction method, folders may need to be manually created or arranged to ensure this convention.

Downloading DynamicAtlas code:

Code for the MATLAB-based atlas methods is located at the following Github repository:

https://github.com/npmitchell/dynamicAtlas

The code can be downloaded to a folder of choice from the GitHub website, or, preferably, cloned using the command line, e.g. with the command…

git clone https://github.com/npmitchell/dynamicAtlas.git

…as depicted with the following screenshots before and after executing the command:

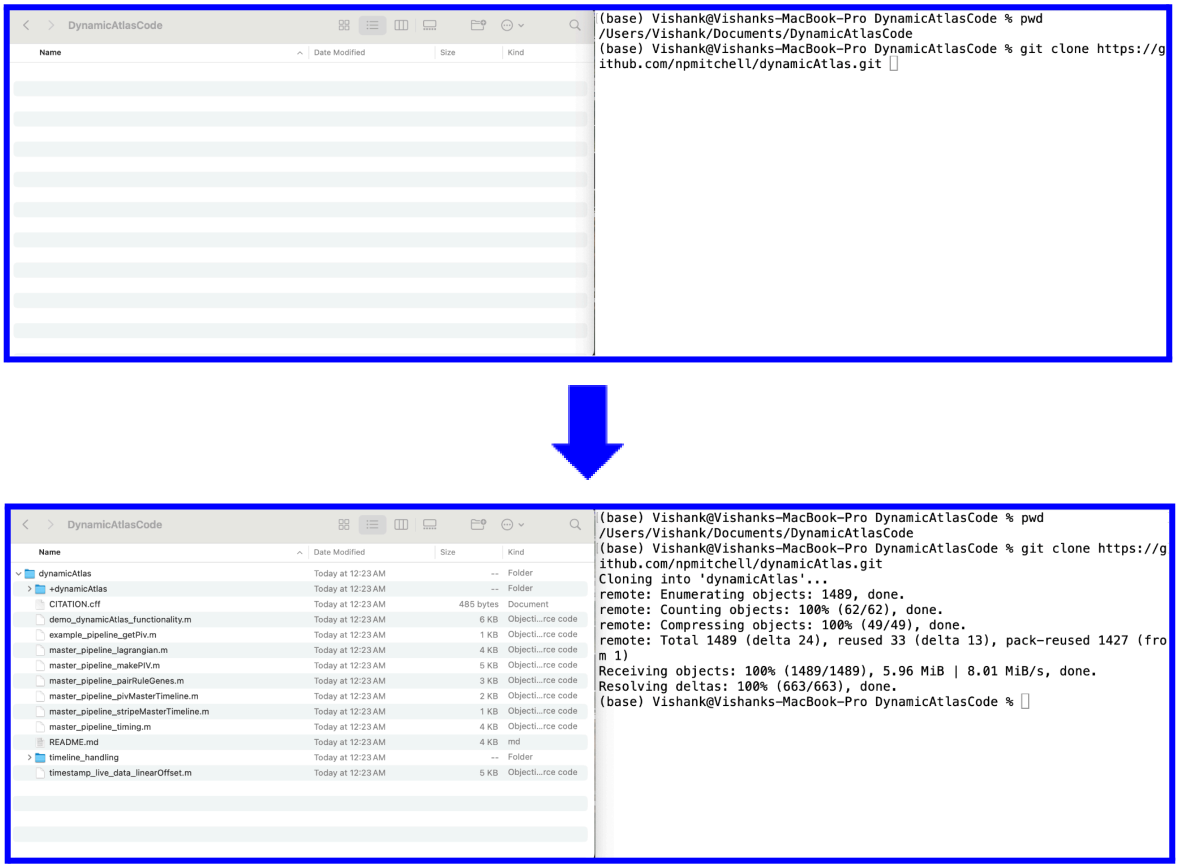

This folder, together with the MATLAB native library and toolboxes, comprises all the dependencies needed to run the MATLAB-based code. The code that has been written for this tutorial is contained within the…

demo_dynamicAtlas_functionality.m

…script, and what follows below will be a walkthrough of this code demonstrated on the above demo dataset.

Demo code walkthrough: Data Conventions

The Atlas code requires data to follow standard conventions across experiments, in terms of both naming conventions and content. The demo code demonstrates the creation of a master timeline using gene expression geometry to determine morphological time (see Figure 2 of the manuscript). Shown below are the minimal inputs required for running this code, for an example live dataset (ID: 202001141730):

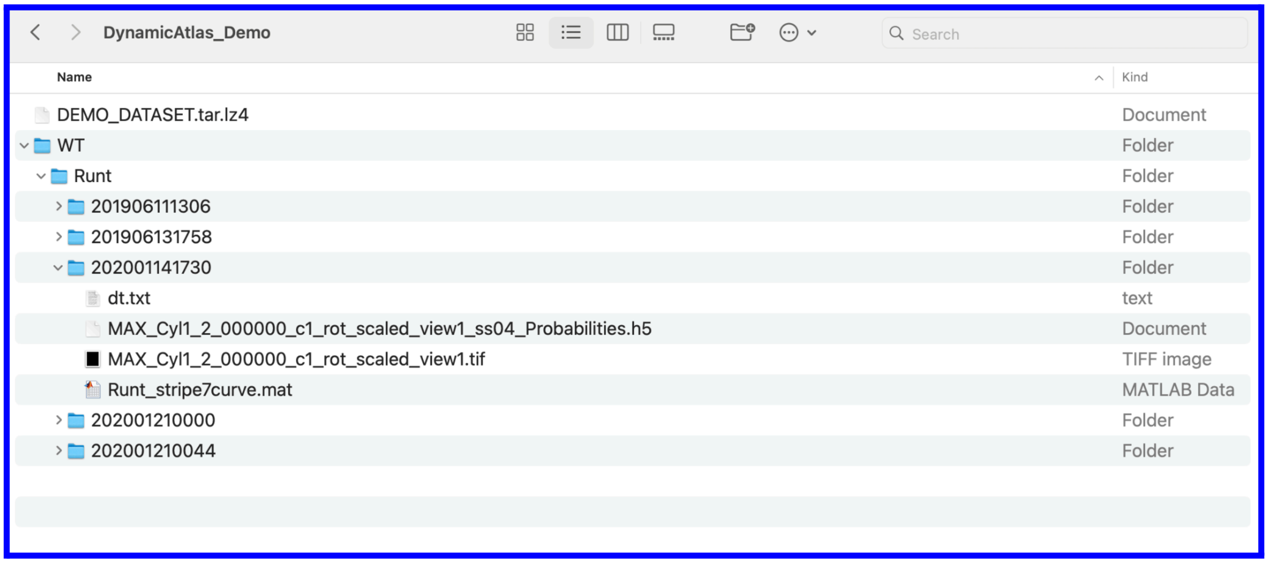

There are four files required. First, is a text file, 'dt.txt', which contains the time resolution of the data as a single number, in minutes per frame. The demo data was taken at 1 minute time resolution, so the file just contains the number 1, as shown:

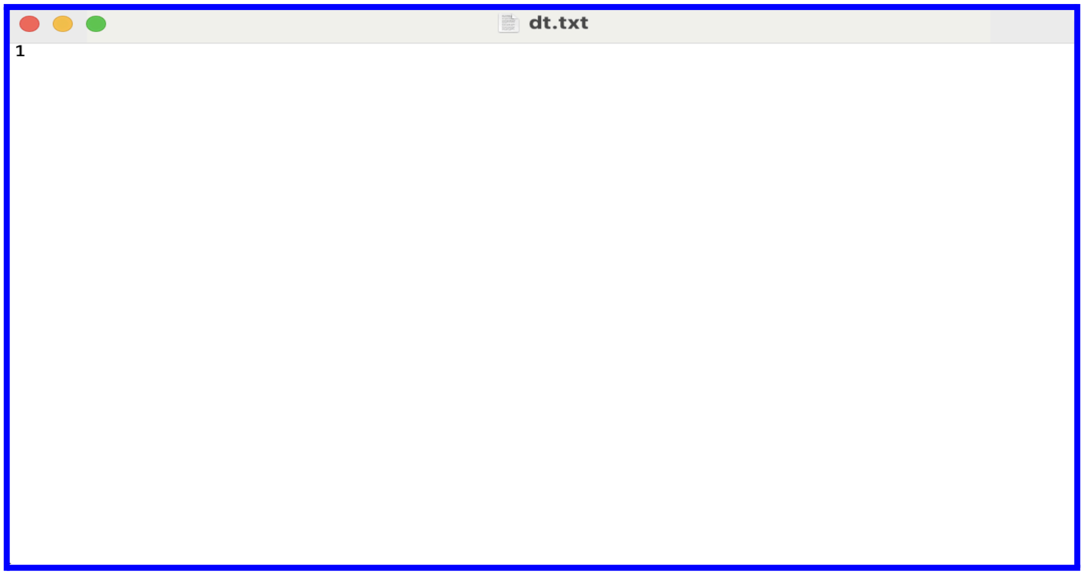

The second file is an h5 ‘probabilities’ file where each pixel is assigned a probability of being part of the expression pattern, based on a user’s training. This can be generated with the program Ilastik (https://www.ilastik.org/), or with a similar pixel classification algorithm. Shown below is the example h5 file loaded into Fiji (via File > Import > HDF5, note that the dataset must be selected and the dimensions listed must match those of the stack):

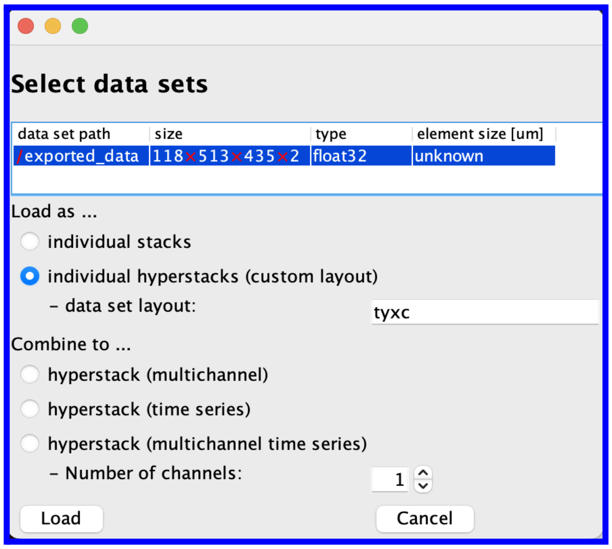

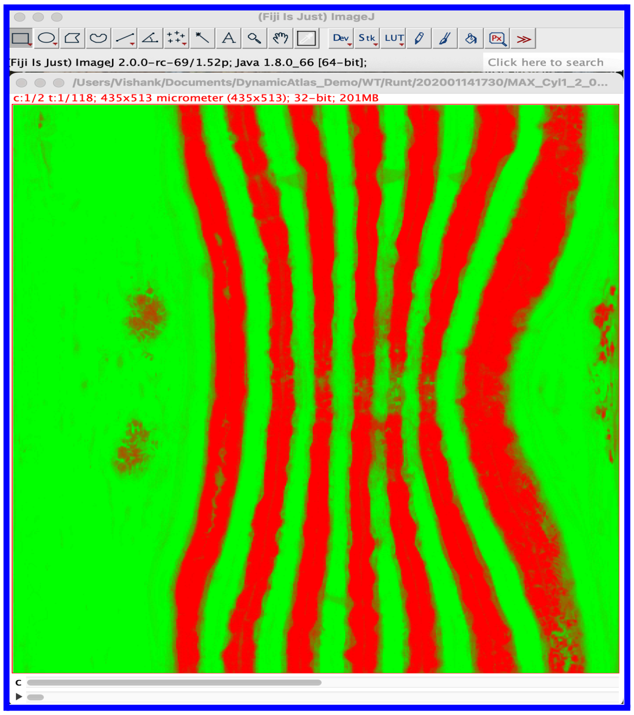

The third file is the data itself, a multipage tiff stack showing a movie of the projection of the surface over time. Opening this example file in Fiji shows the following:

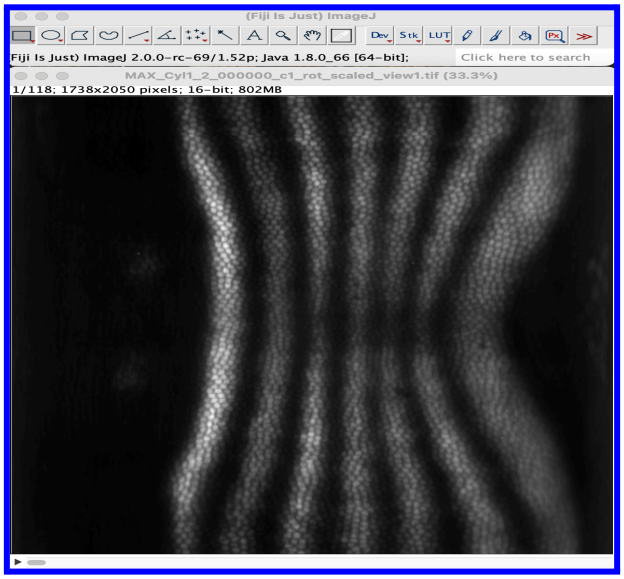

By convention, all of the data take the form of map projections with spatial size of 1738x2050 pixels (this resizing yields an error of order ~4%, see SI). This enables all datasets to be quantitatively compared to each other in space. The datasets are also all named with the convention: “MAX_Cyl1_2_******_c*_rot_scaled_view1.tif” where the asterisks are replaced with time and channel respectively, though a different convention can be chosen as long as suitably specified in the options fed into the function.

The fourth file is a Matlab variable in the .mat format containing the xy-coordinates of the detected expression pattern boundary, in this case the boundary of Runt stripe 7 (‘Runt_stripe7curve.mat’). The atlas code has functions for performing the boundary detection (see dynamicAtlas/+dynamicAtlas/stripeExtraction) but this can also be done using another method of choice, as long as the result is saved in the same format. Shown below are the Matlab variable, and the (x,y) points plotted in space, for the first frame of the example movie, with data for the curves contained in ‘stripe7curves’: Each

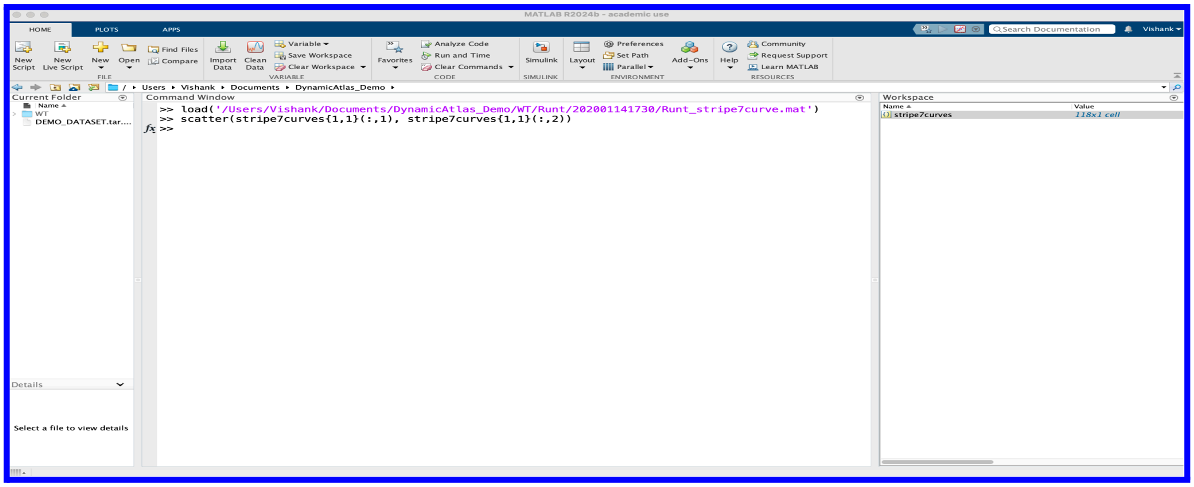

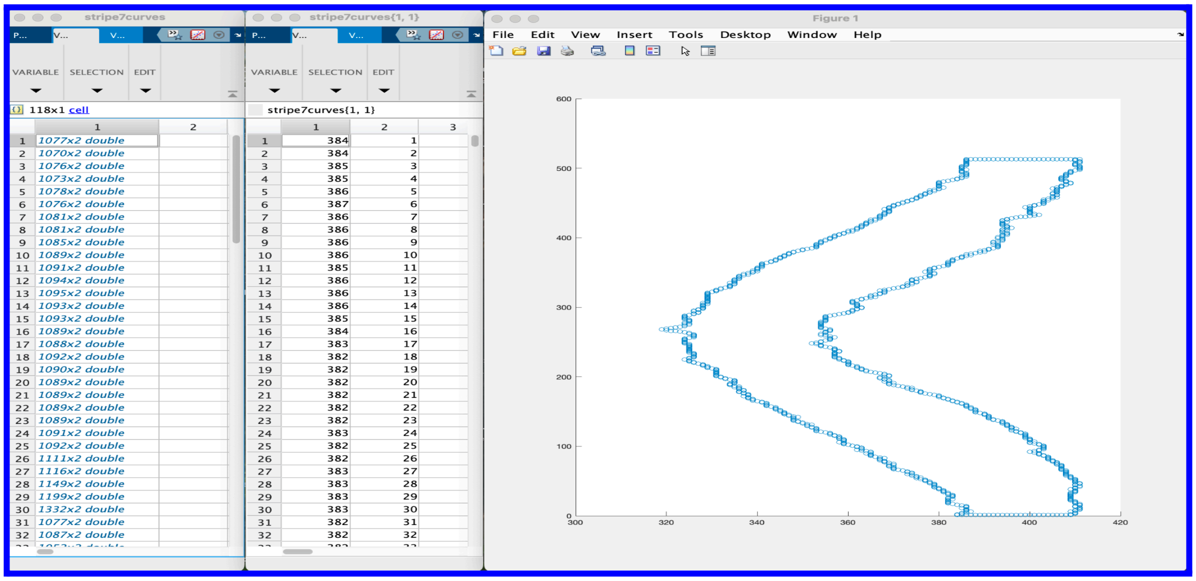

live dataset requires these four files. Fixed datasets do not require the .h5 file or the .txt file, but each fixed dataset does require the .tif file and the .mat file.

For the fixed datasets, the .mat file denotes the curve by a similar variable (stripe7curve). Additionally, the file will have three extra variables, denoting the coordinates as fractional positions along the projection (stripe7curve_frac), and also variables indicating the AP and DV dimensions (szAP, szDV), as measured by the pixel dimensions of the image projections they came from. In the convention followed by the existing .tif data (described above), szAP will be 1738, and szDV will be 2050. An example .mat variable for a fixed dataset is depicted below:

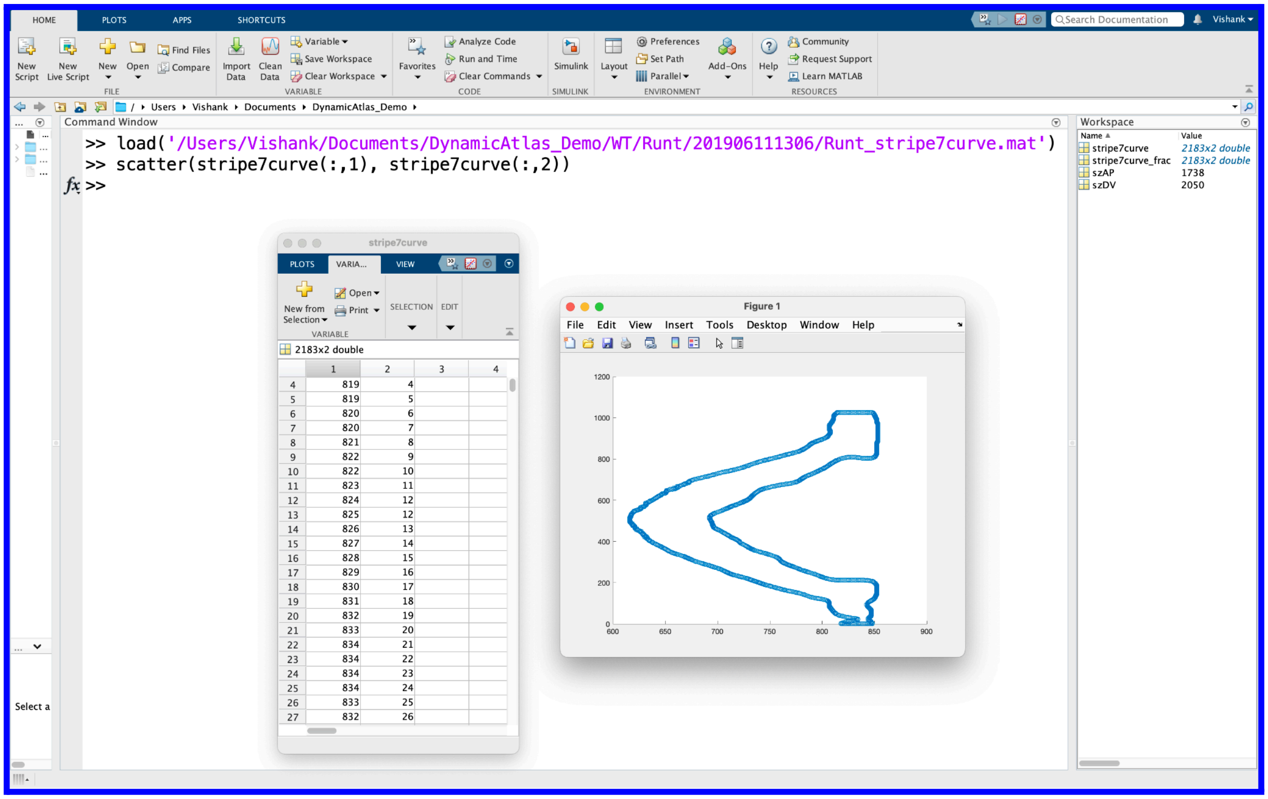

Given the aforementioned files, with the appropriate consistent naming conventions, the core atlas code shown in the demo script can be successfully run.

Note: Other than the files mentioned above, there should be no other files in the data folders. If there are, they are extraneous files caused by the extraction process. Delete any such extra files, since they can cause errors in Code Block II below if they remain.

Demo code walkthrough: Atlas Assembly

[Blocks I - IX]

With the data following the above conventions, the demo code can now be run. The first part of the code involves creating a dynamicAtlas object, which allows one to load data of choice and explore their properties (this is similar to the functionality of the Python-based interface that is also included in the Atlas, see SI). The code is separated into distinct blocks (via the %% symbols), which can be run sequentially in Matlab using the ‘Run and advance’ button in the Matlab editor, as shown:

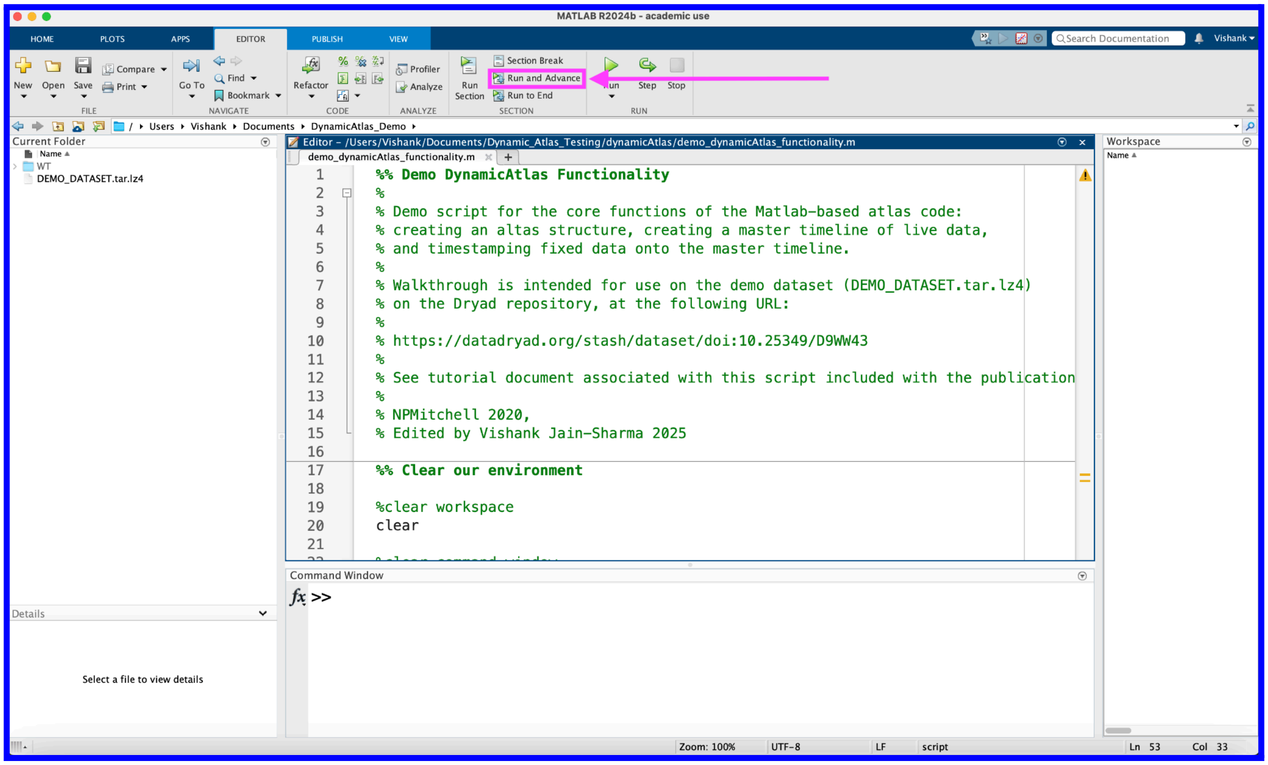

The walkthrough will now show the individual blocks of this code one by one, along with explanations and outputs (generated after ‘Run and Advance’). Note that comments in the code are included with the % symbol. To follow along interactively, open Matlab, and from the dynamicAtlas code folder that was previously downloaded, open the script:

demo_dynamicAtlas_functionality.m

in the Matlab editor (can be done using the ‘Open’ icon in the top left of the window).

The first block of code (after the introductory comments), **Code block I**, merely serves to clear our Matlab environment:

```
%% I. Clear our environment

%clear workspace
clear

%clear command window
clc

%close open figures
close all

```

In particular, this clears our workspace, our command window, and closes all open figures. This helps to avoid duplicate variables carrying over from previous work. The result after clicking 'Run and Advance' will look like this:

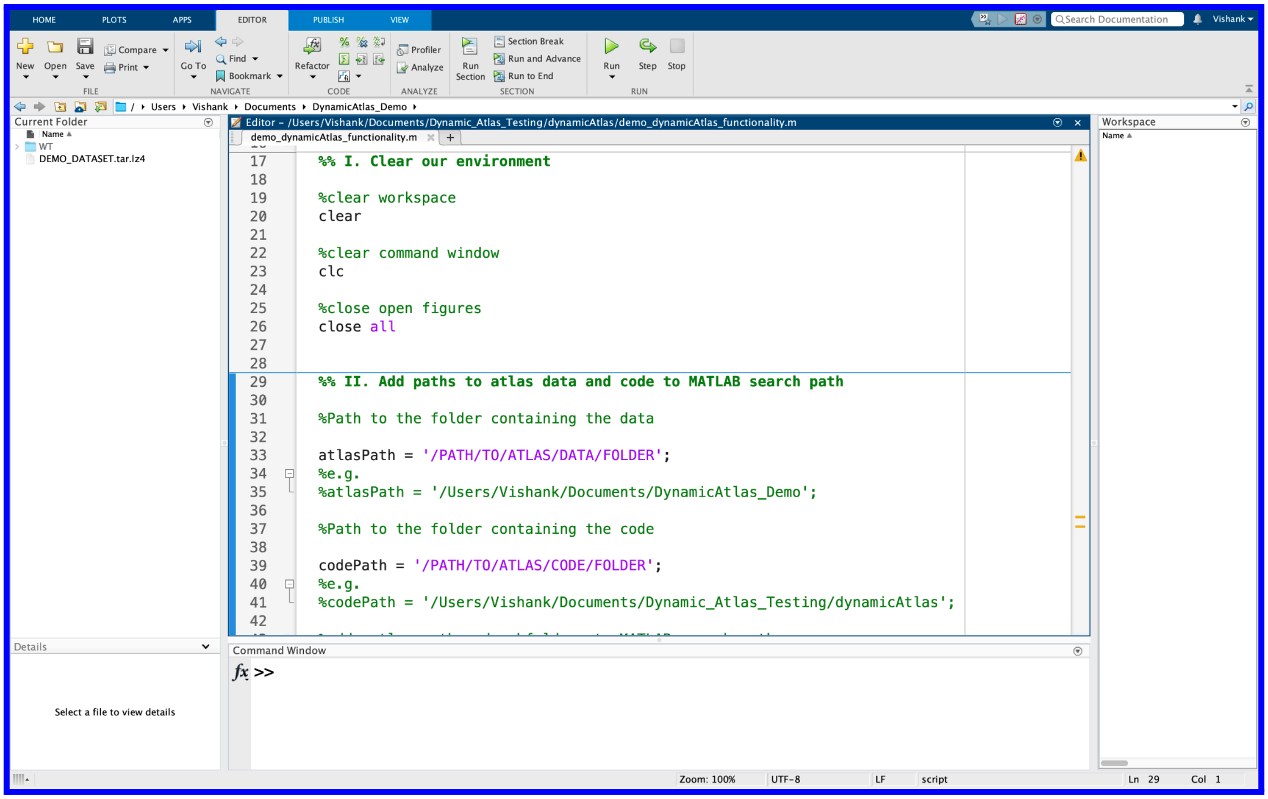

Code block II adds the necessary paths to the Matlab search path, so that Matlab is able to read information from them. In particular: the path to the atlas data, and the path to the atlas code. (If the paths are not correctly defined, Matlab can throw errors simply based on not finding the appropriate files.) The user should set the variable atlasPath

(line 35 below) as the path to the directory that contains the atlas data, i.e. the directory containing the folder ‘WT’. The user should set the variable codePath (line 41 below) as the path to the directory named ‘dynamicAtlas’, i.e. the code folder that the user downloaded to a location of choice. This requires replacing the example paths below with the paths corresponding to the user’s computer. The block adds these paths, and all their subfolders, to the Matlab search path (a warning might appear – ignore it):

```
29  %% II. Add paths to atlas data and code to MATLAB search path
30
31  %Path to the folder containing the data
32
33  %atlasPath = '/PATH/TO/ATLAS/DATA/FOLDER';
34  %e.g.
35  atlasPath = '/Users/Vishank/Documents/DynamicAtlas_Demo';
36
37  %Path to the folder containing the code
38
39  %codePath = '/PATH/TO/ATLAS/CODE/FOLDER';
40  %e.g.
41  codePath = '/Users/Vishank/Documents/Dynamic_Atlas_Testing/dynamicAtlas';
42
43  %adds atlas path and subfolders to MATLAB search path
44  addpath(genpath(codePath));
45
46  %adds code path and subfolders to MATLAB search path
47  addpath(genpath(codePath));
48  pkgDir = fullfile(codePath, '+dynamicAtlas');
49  cd(pkgDir);
50  addpath(genpath('./'));
51  cd(atlasPath)
52
```

The result after clicking ‘Run and Advance’ will look similar to the following:

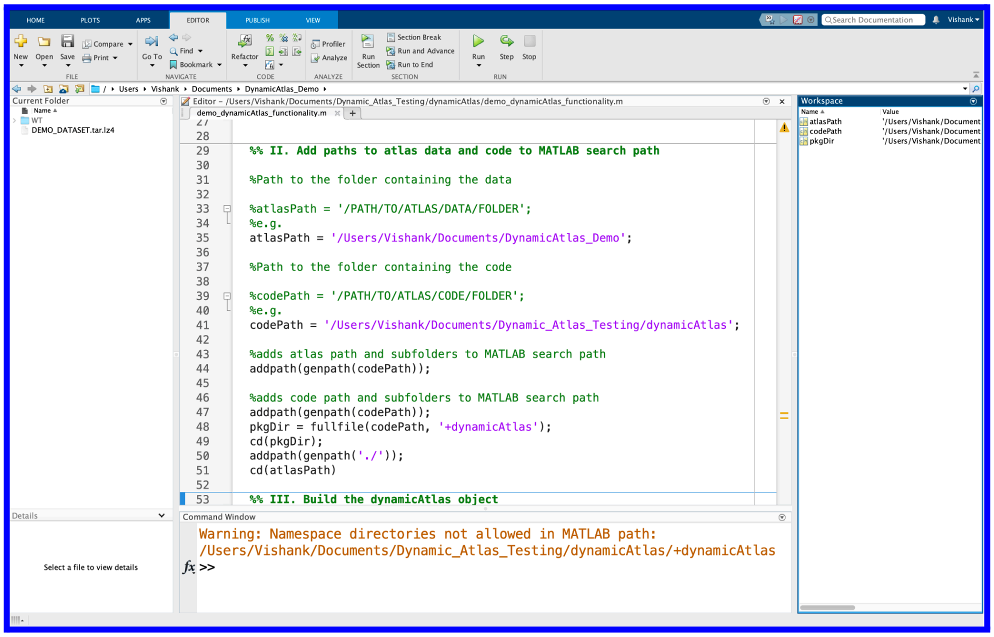

(Note: if extra files were created in extraction, errors can occur; see ‘Data Conventions’)

Code block III builds a dynamicAtlas object, which is a structure that represents the atlas as a whole. The user can choose which genotypes to include in the atlas by specifying them as an argument in the atlas definition (line 68 below).

```
53  %% III. Build the dynamicAtlas object
54  %
55  % Build dynamic atlas with all genotypes in the atlasPath using:
56  % da = dynamicAtlas.dynamicAtlas(atlasPath);
57  %
58  % Or, choose which genotypes to include in atlas as below.
59  % By default, all are included
60
61  %options specifying how to construct the atlas
62  Options = struct();
63
64  %method of timeline construction
65  Options.timeLineMethod = 'realspace';
66
67  %constructing the atlas with wildtype genotypes (WT)
68  da = dynamicAtlas.dynamicAtlas(atlasPath, {'WT'}, Options);
69
```

Here, the demo atlas only has the wildtype genotype, so the argument used is just {'WT'}. In general, this should be specified as { 'Genotype1', 'Genotype2', … }. If no argument is specified, all genotypes are included by default. The code attempts to find information about the data contained within the folders corresponding to these genotypes, and loads in all the metadata it can find. The output will look like this:

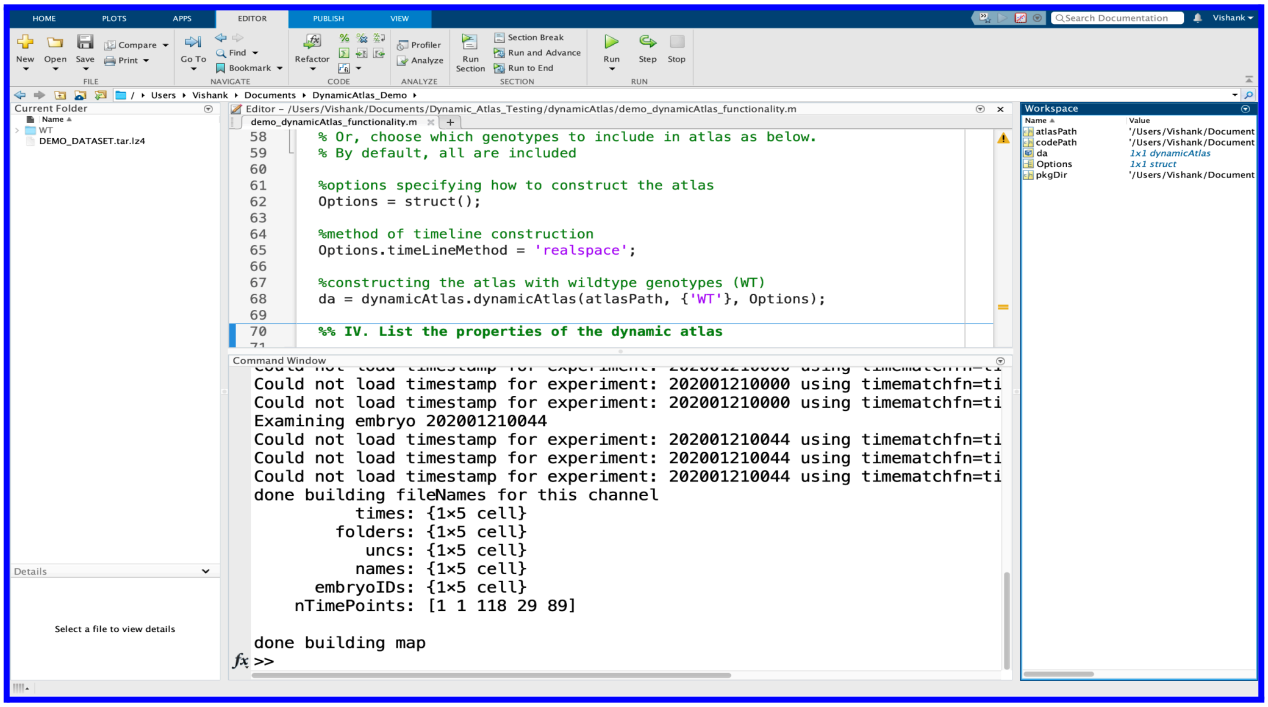

Shown are examples of the information found for the demo dataset. For example, there are two fixed datasets here (201906111306 and 201906131758), and so there are two corresponding entries of 1 in nTimePoints. The variable ‘da’ here represents the atlas object, and other methods in the code interact with this object to perform computations.

Code block IV lists the properties of the dynamicAtlas object:

```
%% IV. List the properties of the dynamic atlas

properties(da)

```

and will show an output like:

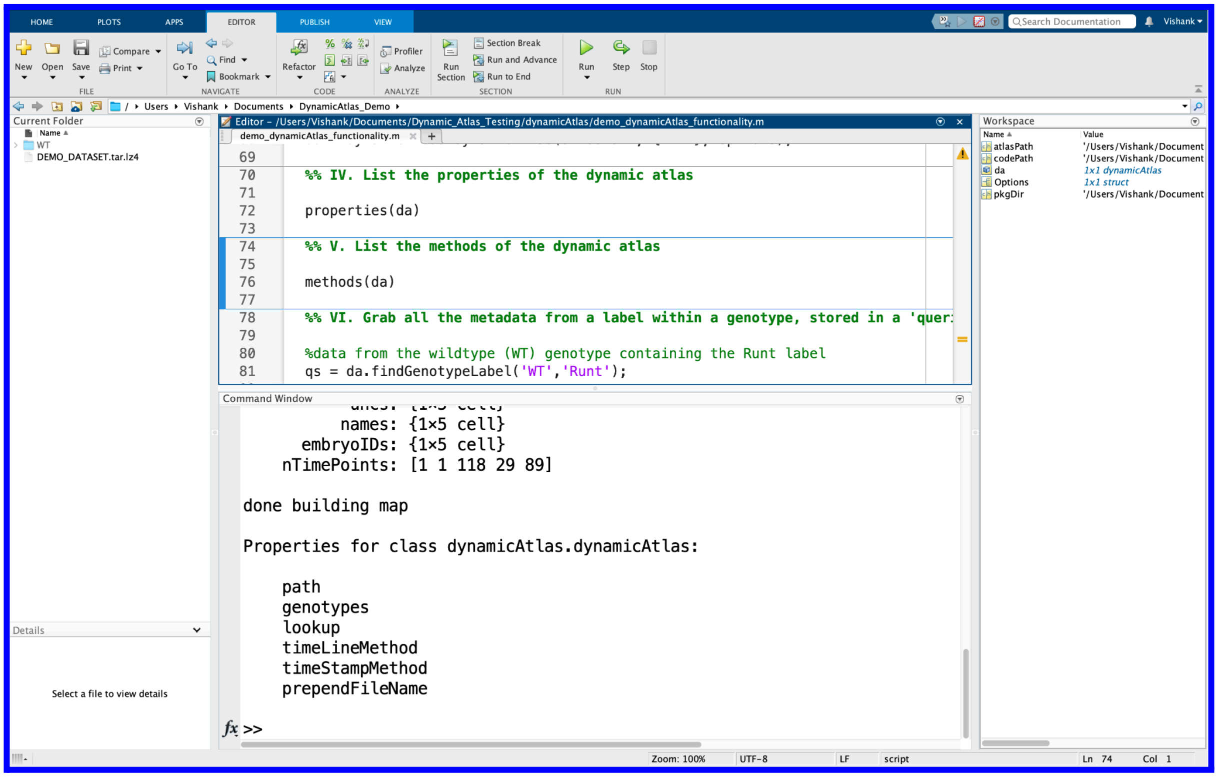

These are the fields of information contained in the ‘da’ variable, which can also be examined by double clicking on the variable in the Matlab workspace.

Code block V lists the methods in the dynamicAtlas software that can be invoked by the dynamic atlas object:

```
%% V. List the methods of the dynamic atlas

methods(da)

```

and will show an output like:

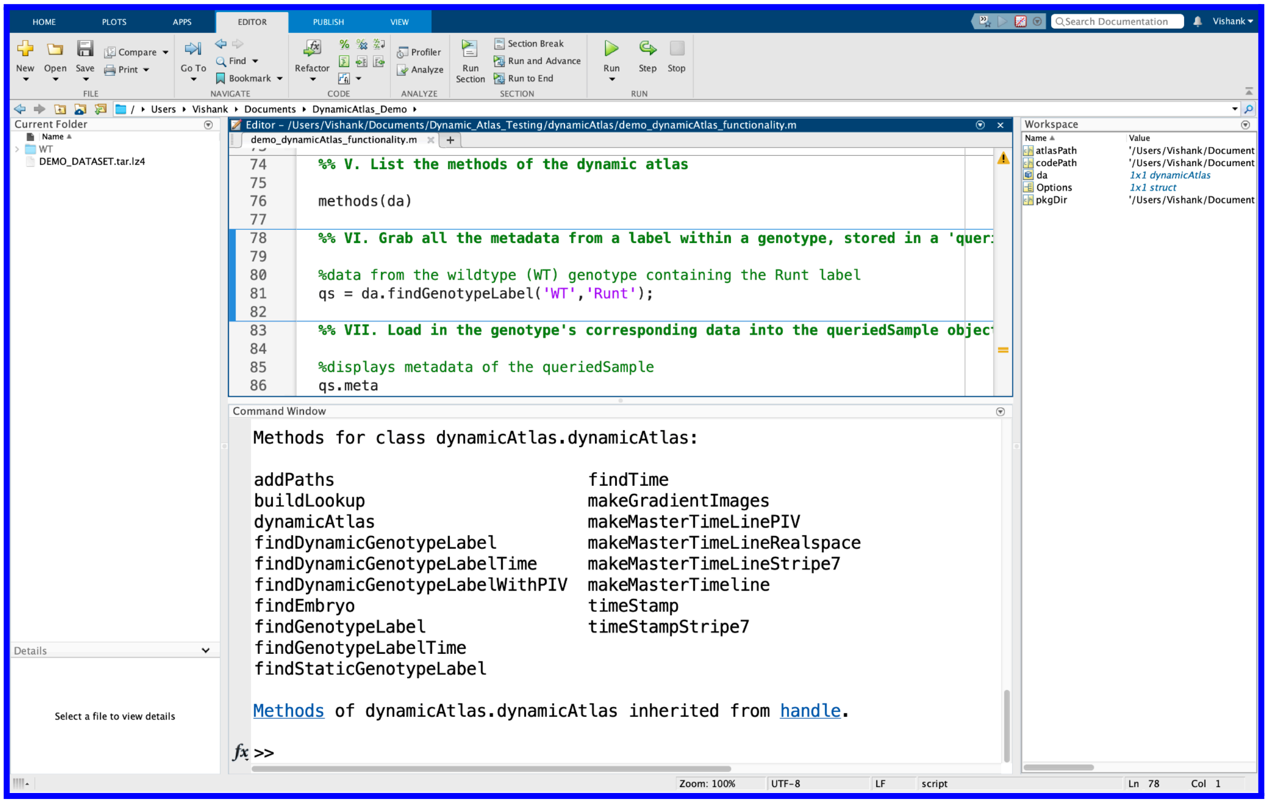

These can be invoked by any dynamicAtlas object, such as ‘da’ above.

Code block VI grabs all the metadata from a label (e.g. Runt) within a given genotype, and stores this within an object called a queriedSample.

```
%% VI. Grab all the metadata from a label within a genotype, stored in a 'queriedSample'

%data from the wildtype (WT) genotype containing the Runt label
qs = da.findGenotypeLabel('WT','Runt');

```

There is no additional output to the command window, but the queriedSample variable ‘qs’ is generated, as below. The fields will contain the metadata, and places where data can be stored.

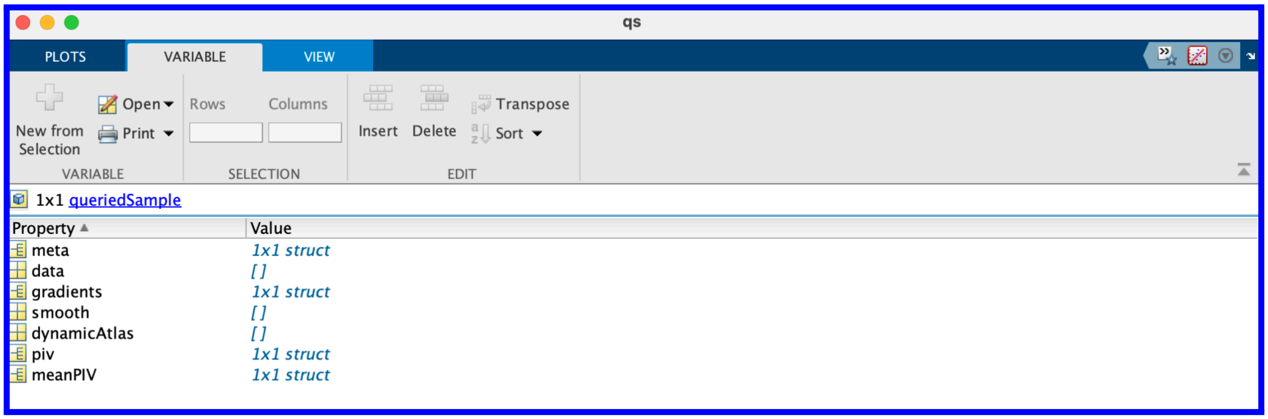

Code block VII uses the metadata pointed to by qs to load in the data, and displays the contents of qs to the command window.

```
%% VII. Load in the genotype's corresponding data into the queriedSample object

%displays metadata of the queriedSample
qs.meta

%loads in the corresponding data
qs.getData()

%displays the data that was loaded
qs.data

```

The output will look like below:

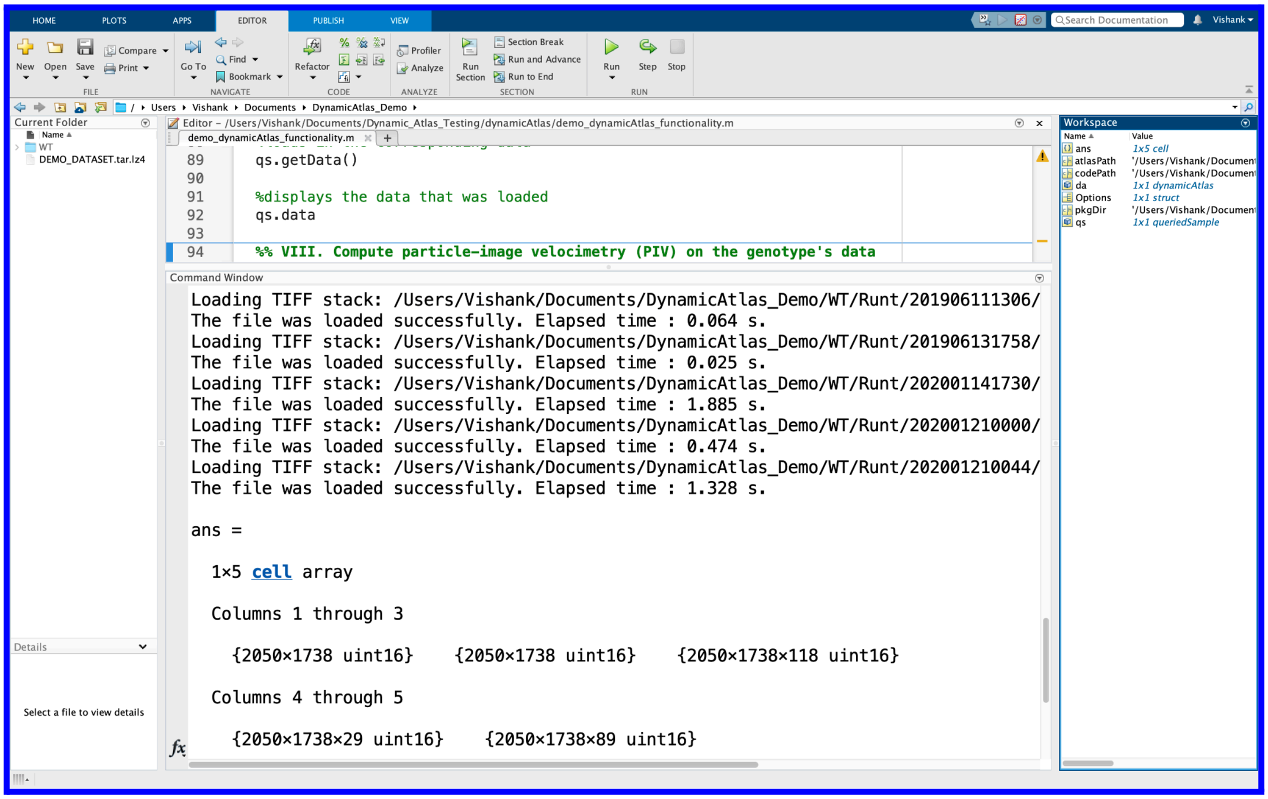

The data contained within the tiff files of the folders assigned to a label (here, embryos labeled for Runt within the WT genotype) are loaded into qs one by one, and the tiff dimensions are displayed in the command window. Contents are stored in qs.data, and can be accessed there.

Code block VIII computes the flow on the data within the queriedSample, via particle image velocimetry (PIV). (Note that if PIV has already been computed and one does not wish to re-compute it again, Options.overwrite can be changed to ‘false’, and this will be skipped.)

```
%% VIII. Compute particle-image velocimetry (PIV) on the queriedSample data

%options specifying how to compute the PIV
Options = struct();

%overwrite existing PIV computations
Options.overwrite = true;

%computes PIV on the queriedSample data
qs.ensurePIV(Options);

```

When this code block is run, the data from the live datasets will be loaded in. Windows similar to the following will pop up as the code is running:

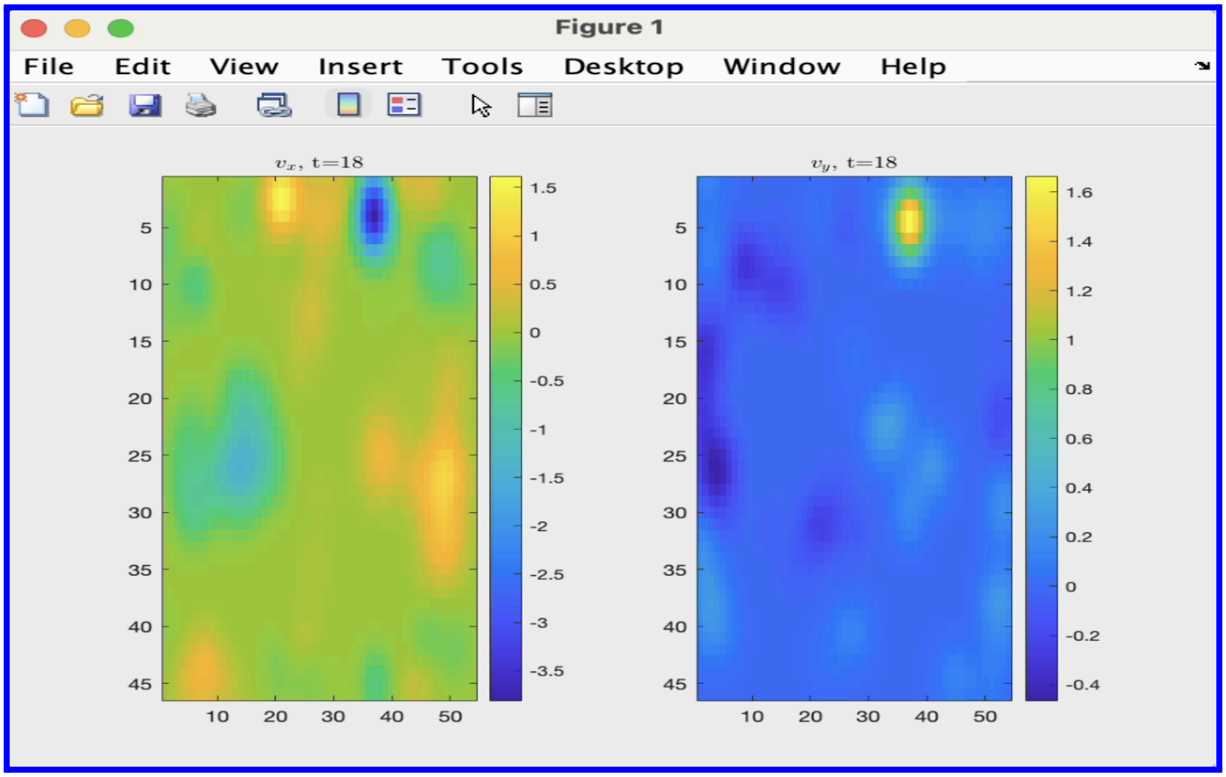

These windows show the computations as they are being performed, displaying the x and y components of the velocity respectively in space, here at time frame 18. When the computations are done, two new folders will be generated in each live dataset’s folder — PIV and PIV_filtered (displayed opened up below) — as shown:

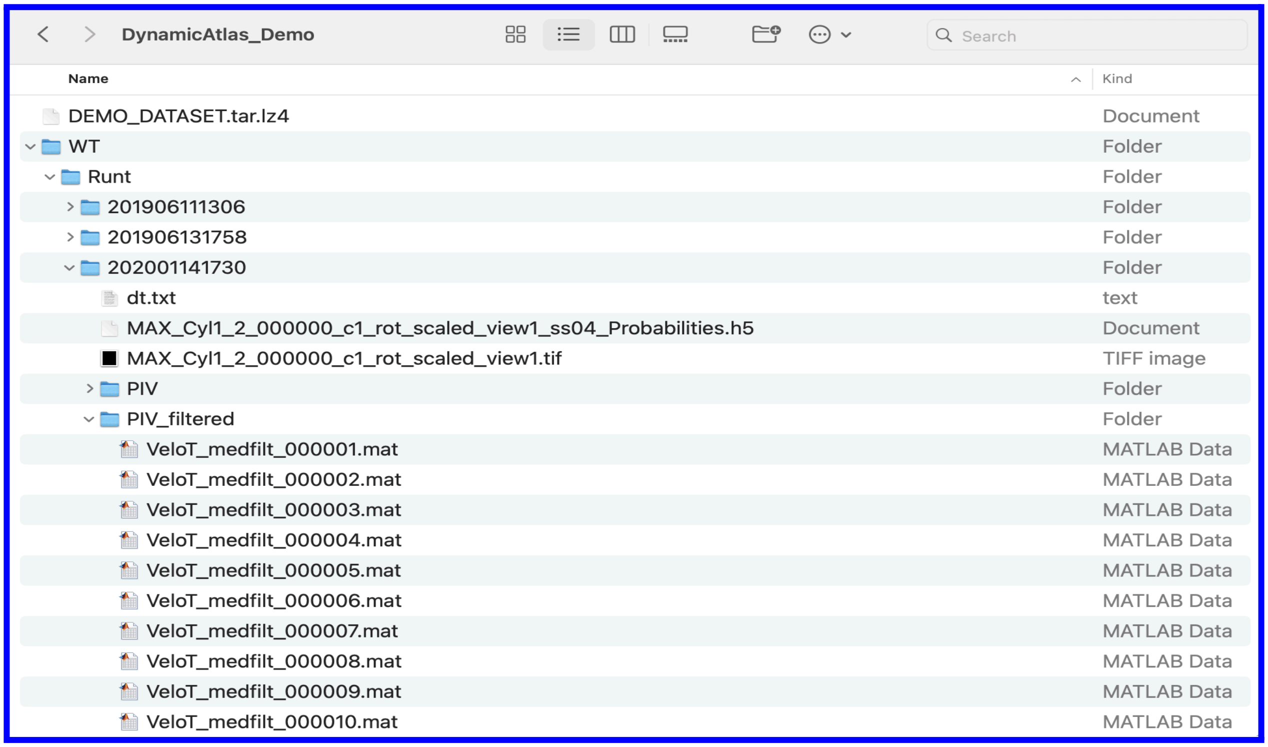

Both contain results of the flow field computation. ‘PIV’ contains the computed flow field, and ‘PIV_filtered’ contains the result of performing a median filter of order 3 on the contents of ‘PIV’, to smooth the flow field in time. Each .mat file contains the velocity components VX and VY at the indicated time frame. The output of the code block in Matlab will look like below:

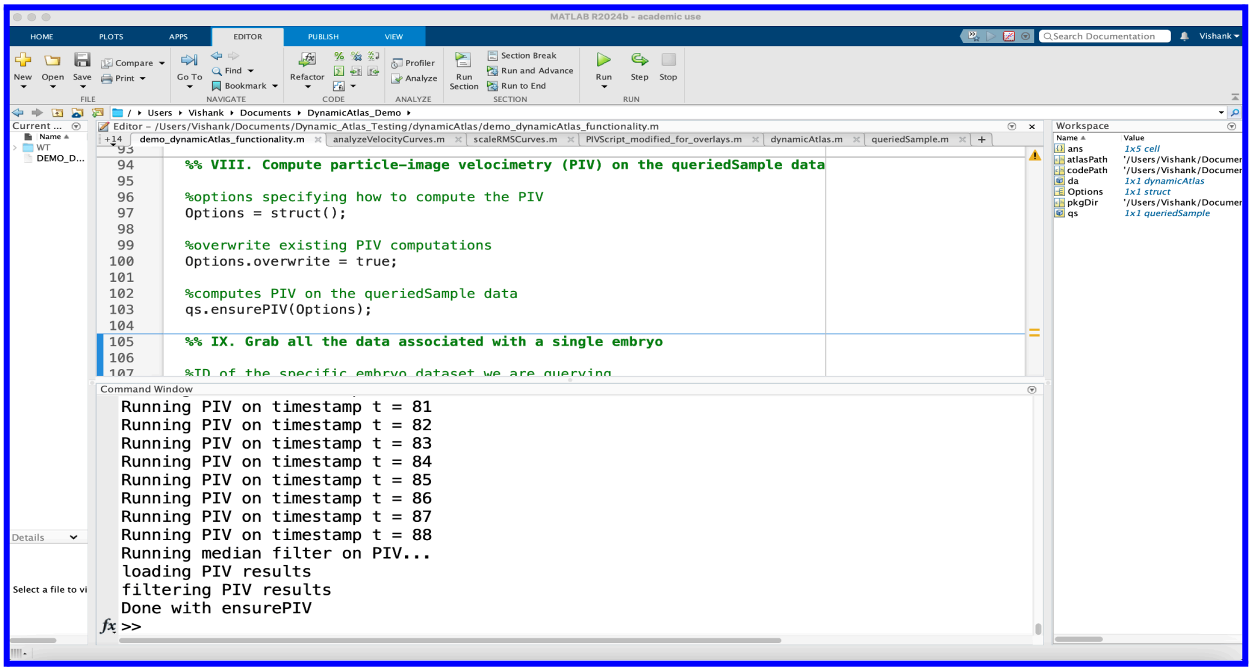

Code block IX loads in the data associated with a given embryo, by defining a new queriedSample (qs2) with just one embryo (here, ID: 202001141730).

```
%% IX. Grab all the data associated with a single embryo

%ID of the specific embryo dataset we are querying
embryoID = '202001141730';

%queriedSample with just this embryo's metadata
qs2 = da.findEmbryo(embryoID) ;

%gets the data of this embryo
qs2.getData();

```

The data for this embryo will be stored in qs2. The output will look like below:

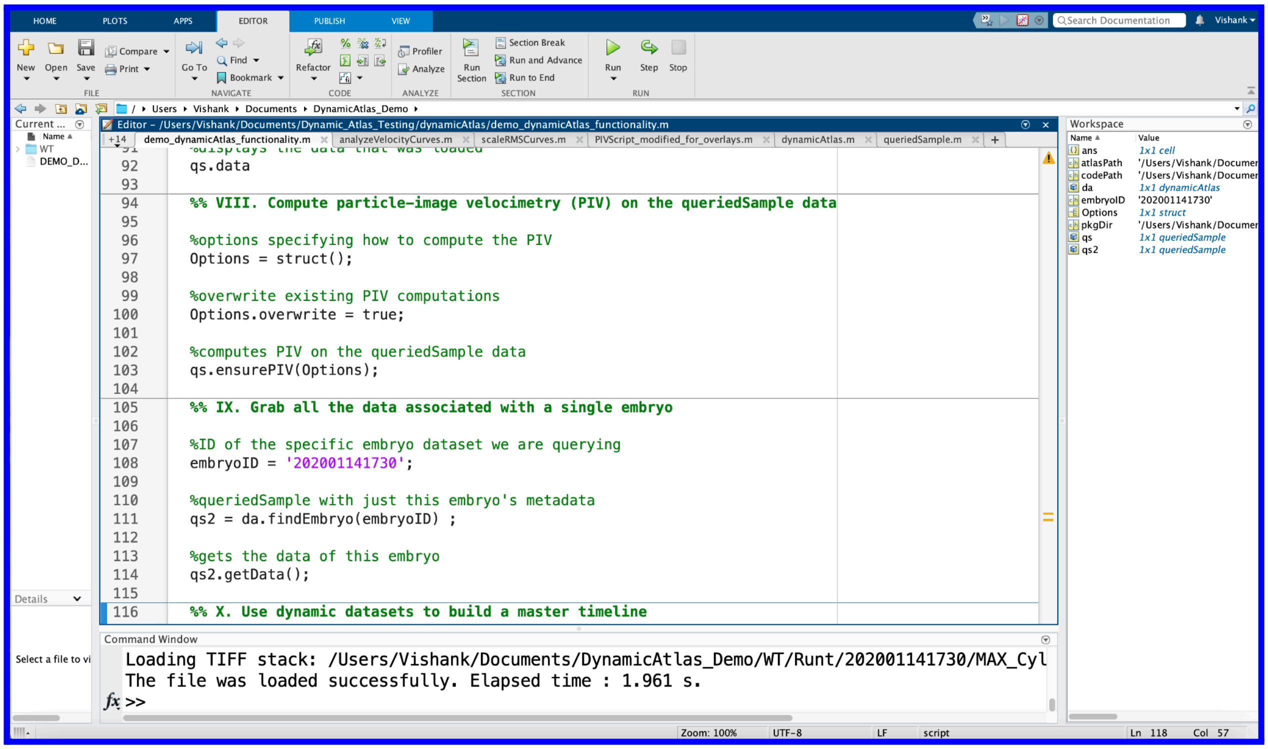

This method can be used to examine properties of any embryo of interest within the overall dataset.

Demo code walkthrough: Timeline Creation

[Block X]

With the data loaded, and defined as above, the morphological timeline creation features of the atlas can now be used. Live datasets are systematically cross-correlated with each other, correspondence curves are found between their timelines, and these timelines are then annealed together to create one consensus timeline. This timeline creation procedure is mostly automated, but a few steps require manual input, and these are explained below. (Note: if the code gets interrupted, this section can just be run again, as the code is written to pick up where it left off when it was last attempted.)

Code block X invokes master timeline creation with options of the user's choosing.

```
%% X. Use dynamic datasets to build a master timeline
%
% Aligning dynamic runt nanobody data against each other

%options specifying how to compute the timeline
Options = struct();

%saving the plots generated while computing the timeline
Options.save_images = 1;

%makes the master timeline from the live WT datasets with the Runt label
da.makeMasterTimeline('WT','Runt', Options)
```

The first step is to choose a live dataset to be the 'master timeline designee'. This will be the dataset which all others are initially compared to for the alignment, but later just becomes an indicator of the equivalence classes of the timeline. The following prompt is displayed in the Matlab command window to do so, by entering a dataset index:

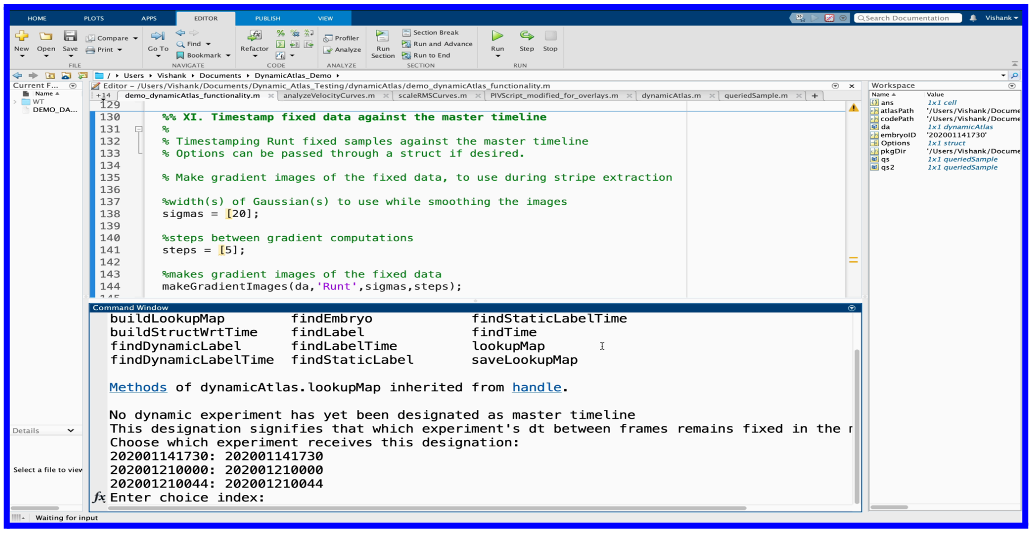

(Note that any choice can be made, but choosing a longer dataset tends to yield better time matching.) If, for example, the first live dataset is chosen (dataset 202001141730 here), the number 1 should be entered, and the following output will result:

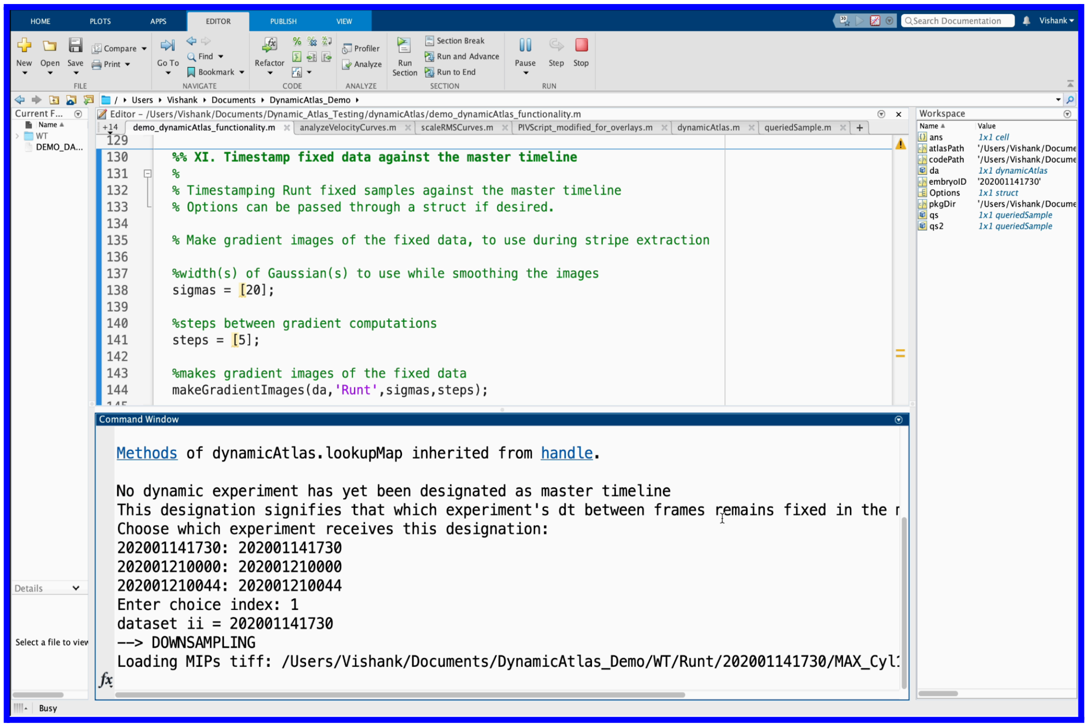

The script will also create a text file, 'master_timeline_designee.txt', in the folder of the selected dataset, as shown:

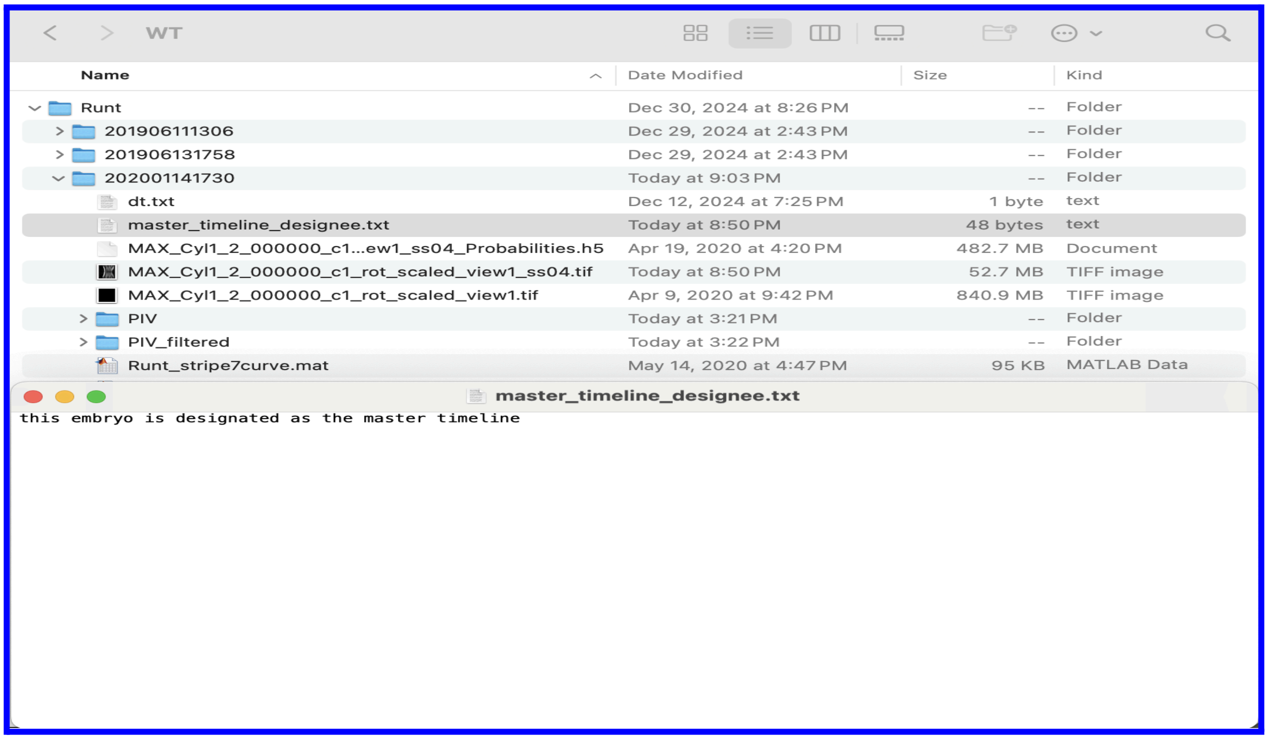

The live datasets will then all be loaded in.

Next, all the live datasets are systematically correlated with each other (cross-correlation), including to themselves (auto-correlation), yielding a correlation matrix for every combination. These correlations are computed using the Pearson correlation coefficient between the images (see Methods). This step may take some time, because the number of comparisons made scales quadratically with the number of live datasets present. After a correlation matrix is computed, it is briefly displayed as a heatmap. Shown is an example of such a heatmap:

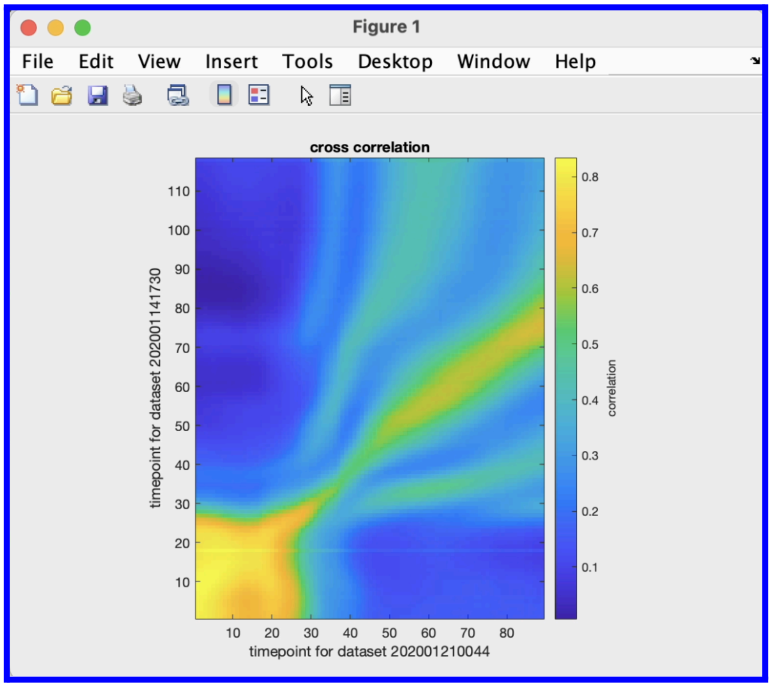

This step is fully automated, and the user does not need to perform any action while these correlation matrices are generated.

The next step is to find optimal correspondence curves through all of these heatmaps (see main text Figure 2). This step requires user input. The program attempts to guess endpoints of the curve based on maximum values of the heatmap at the edges. Users can change these endpoints if they are not correct. Given endpoints, the program attempts first to guess the points which will lie on the correspondence curve by finding high correlation values. If the user indicates these are not accurate, the program then performs the fast-marching algorithm between the endpoints to obtain the curve (see main text Methods). The user can then indicate whether this new result is acceptable, and the path will be stored.

Note that for any prompts indicated on the figures, the user must click on the figure window to interact with them. Else, the user's input will be entered into the Matlab command window, and won't be read in by the interactive GUI.

Below shows the steps described above for an example heatmap. First, the user is prompted (instructions at the top of the heatmap) to check if the path looks ok, based on the guessed points that are displayed:

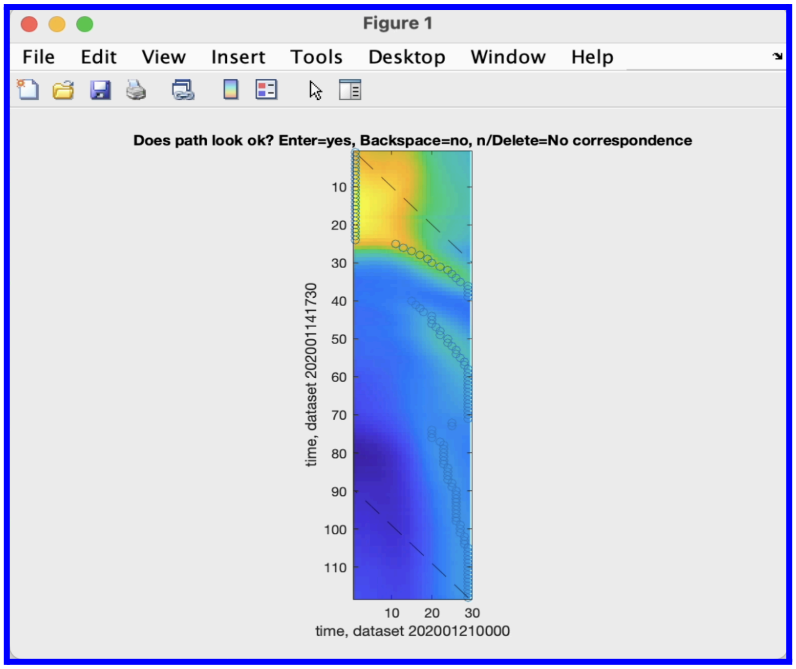

The path should follow a ‘ridge’ of high correlation through the heatmap, and only contain one point per row and column. If yes is selected, the path is accepted, and a new heatmap is shown. If no is selected, the user can then adjust the endpoints of the path and instruct the path to be recomputed. No is selected in this case because there are duplicate points on some columns. (Better paths tend to emerge when No is selected, and the user steps below are performed.)

Next, the user is prompted to evaluate the guessed endpoints (slightly darker red):

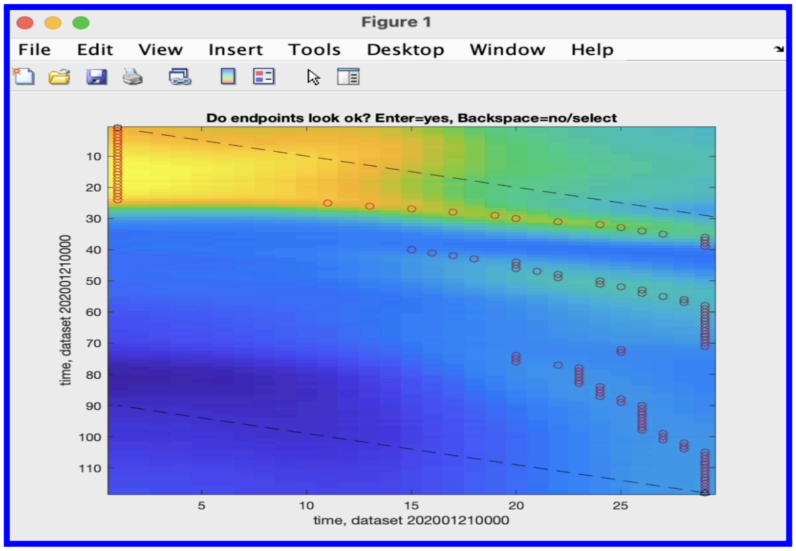

If no is selected, the start point is queried (solid red point):

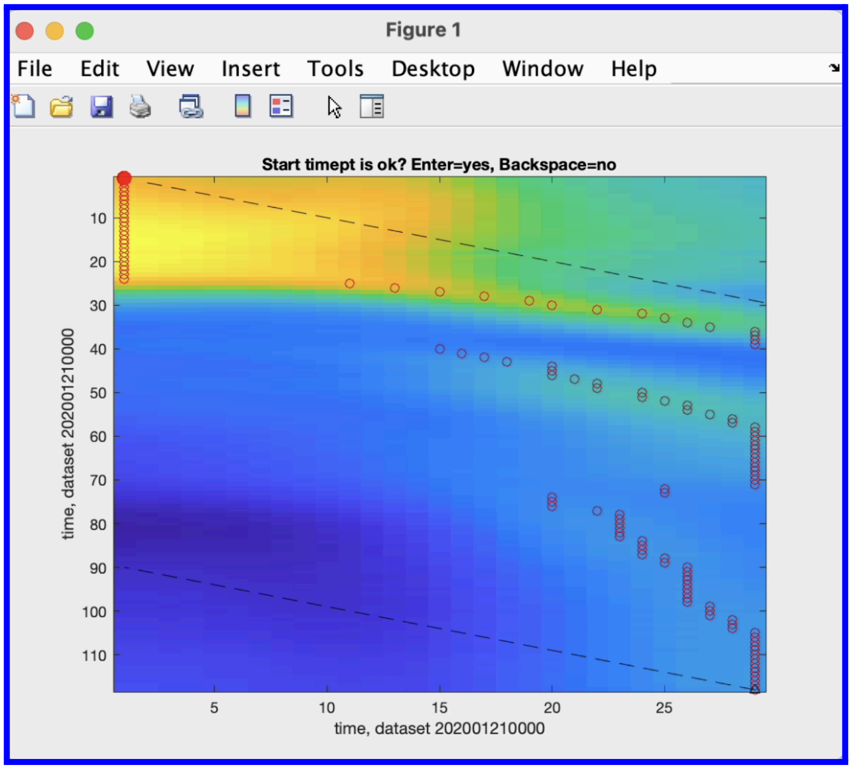

If no is selected, another start point is guessed:

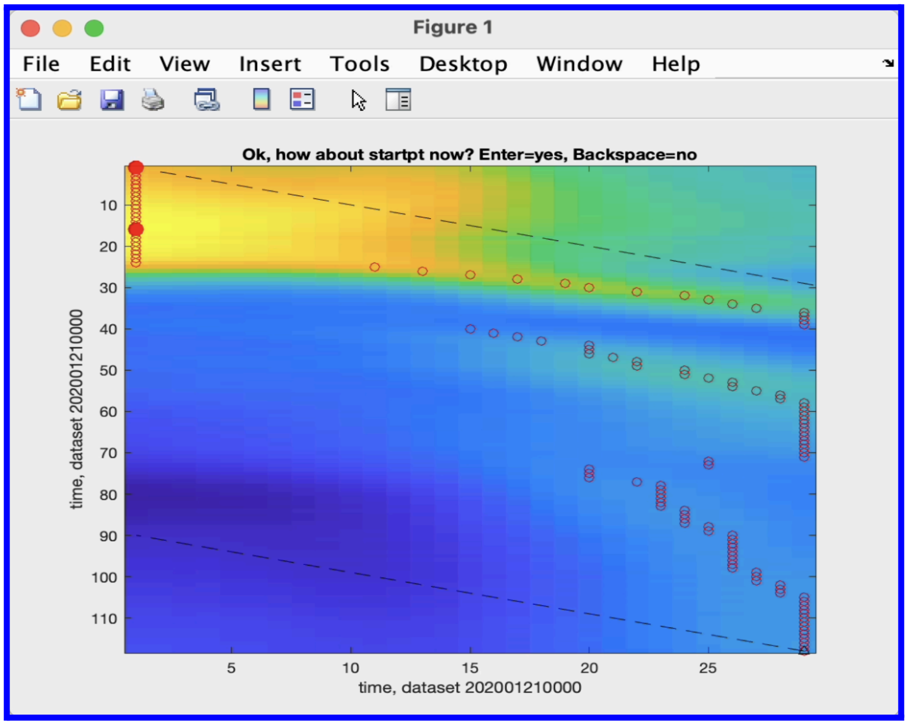

If no is selected, the user will be prompted to click on a point of choosing (not shown here), and the program will find the closest point to the user's input on the boundary of the map. If yes is selected, the start becomes green, and the end timepoint is queried (solid red point):

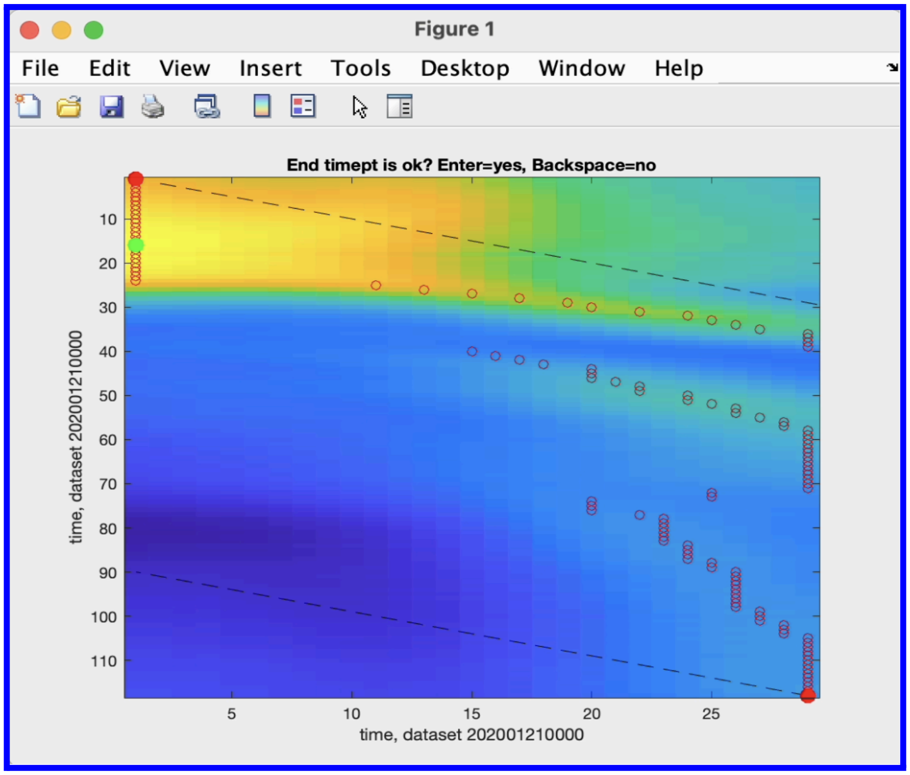

If no is selected, another end point is guessed:

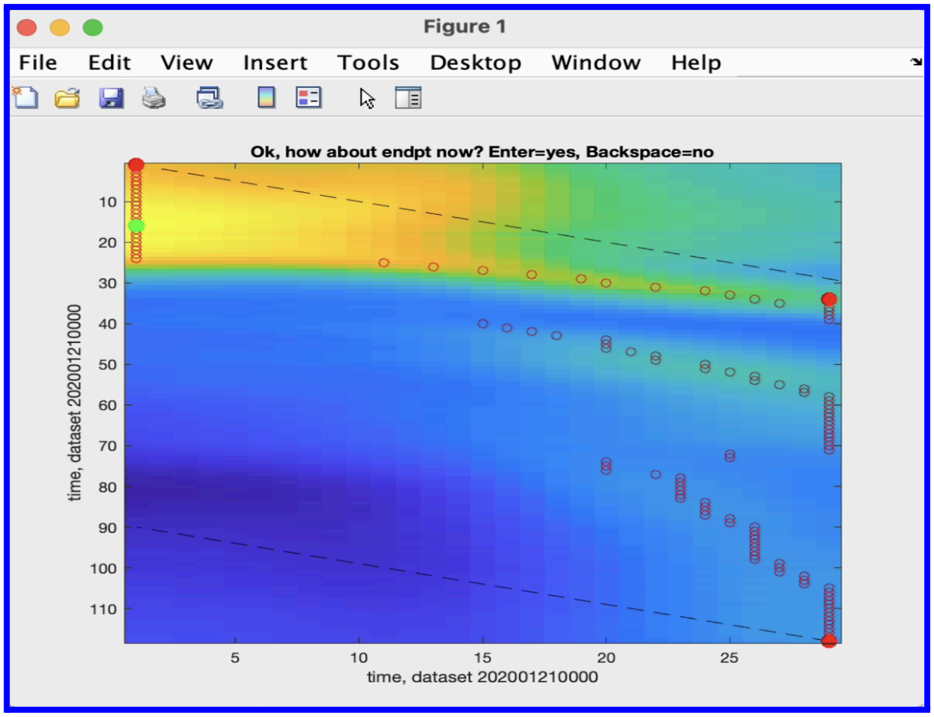

If no is selected, the user will be prompted to click on a point of choosing (not shown here). If yes is selected, both endpoints are now chosen, and the fast-marching algorithm is performed between them, with a window similar to the following popping up during its iterations:

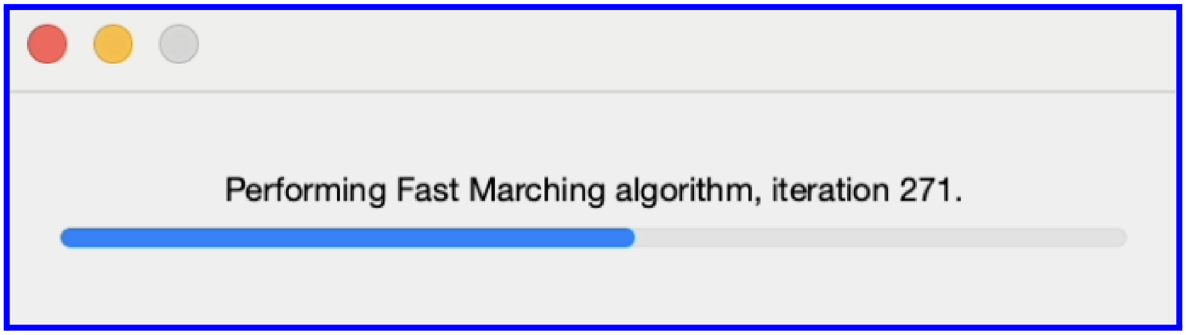

When the algorithm is done, the new guess for the path is displayed by showing sample points on the path (visually, these points should follow a high correlation 'ridge' from end to end):

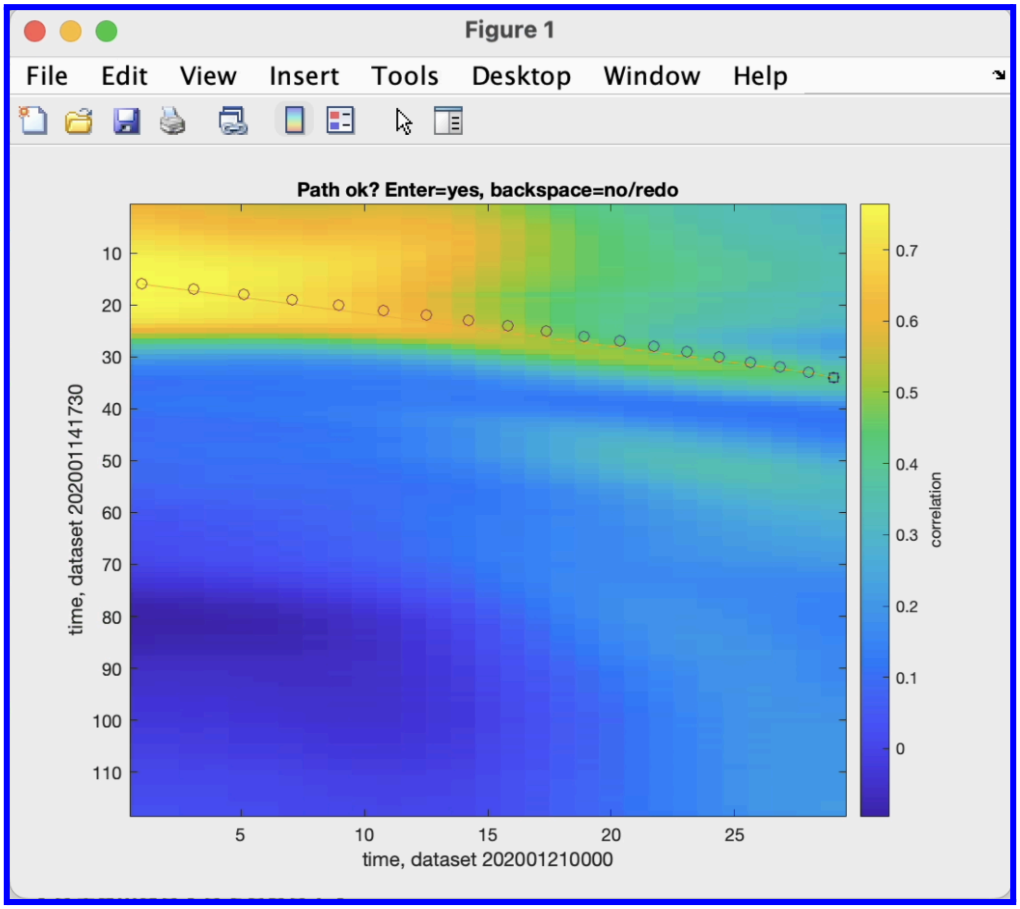

If no is selected, the process can be redone with a new choice of endpoints. If yes is selected, the path is then accepted, a new heatmap is shown, and the above procedures are repeated.

Once the correspondence curves are found for all the heatmaps, the rest of the program follows automatically. Correlations are computed between stripe 7 curves:

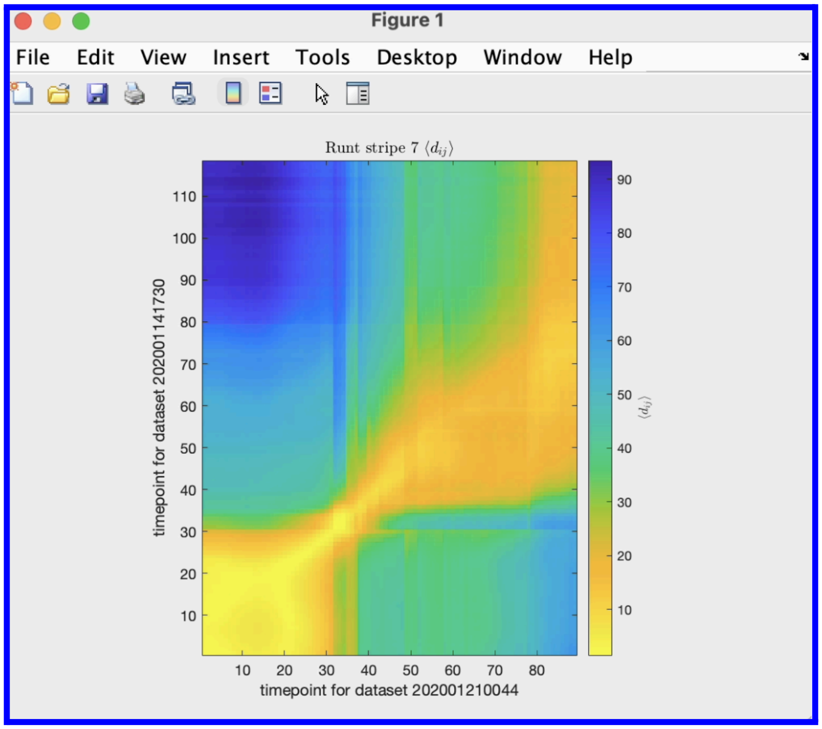

Correspondences are depicted by connecting bonds of a 'network' of time nodes between datasets according to the curves:

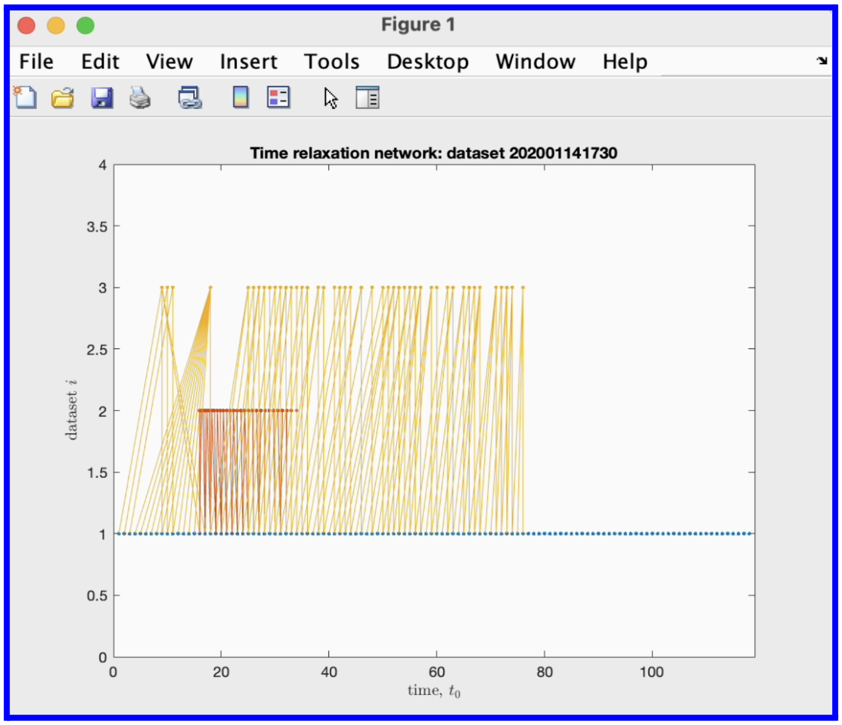

The network as a whole is then annealed together to form an optimal consensus timeline by treating the bonds as springs containing energy, and relaxing the network until an energy minimum is reached (this can take time):

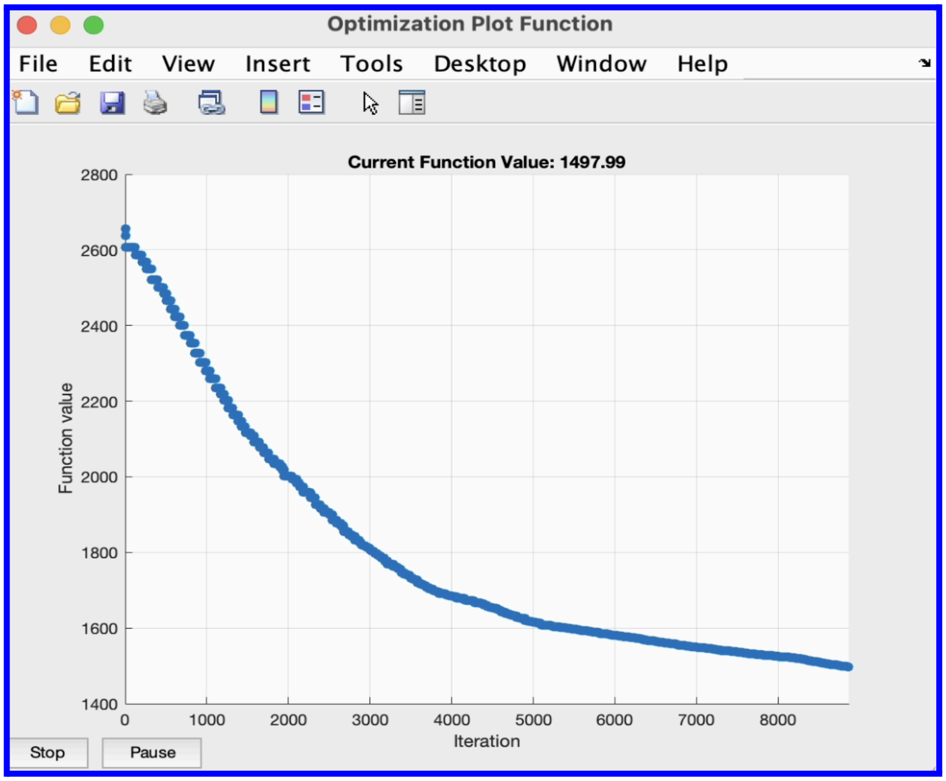

All dataset timestamps can then be represented on the master timeline with an uncertainty given by the chi-squared goodness of fit curve at each point (see main text Methods):

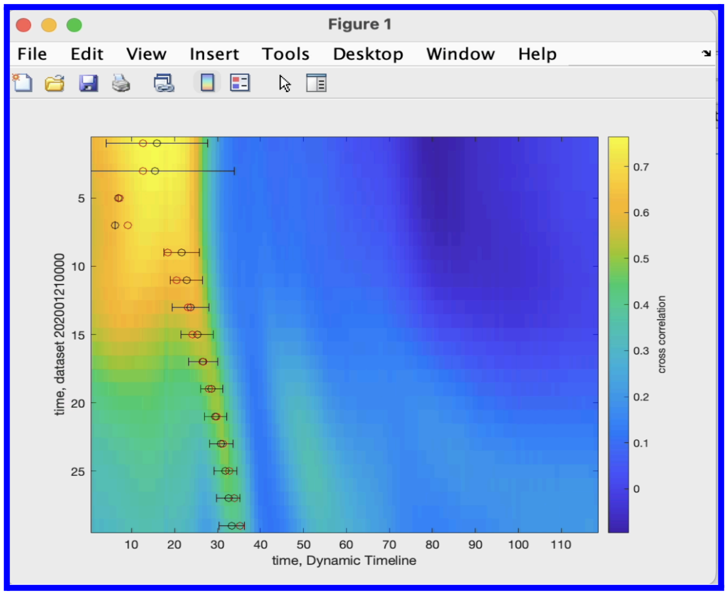

When the timeline creation is done, the code block will show the following output:

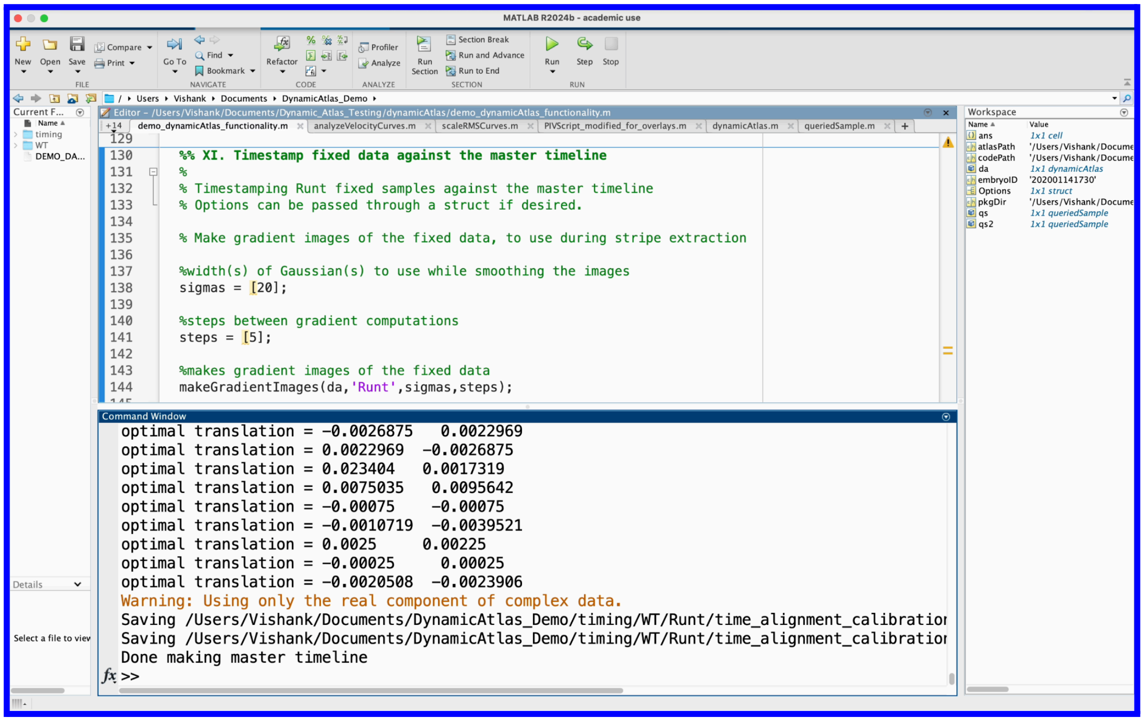

The program will save the time matching data to each of the live folders, in the form of both a .txt file and .mat file (same data in both). The data consist of the sequence of timestamps from the live dataset onto the master timeline. The second column indicates the resolution of time stamping (for this data, 1 minute, as this was the interval between frames for all movies):

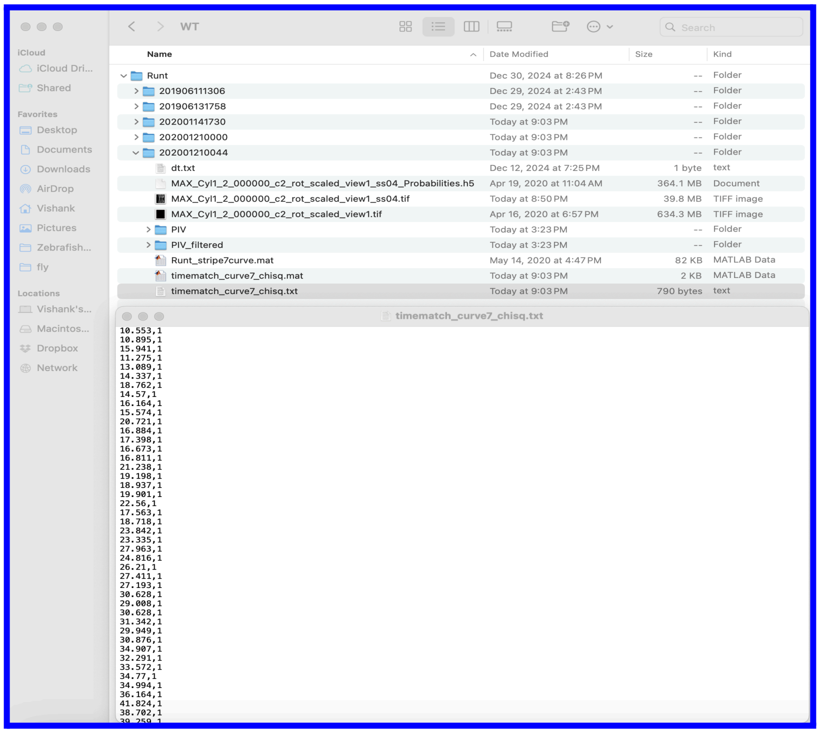

Additionally, the program will generate a ‘timing’ folder for all timeline-related data and figures, on the same folder level as the genotype folder (WT in this case):

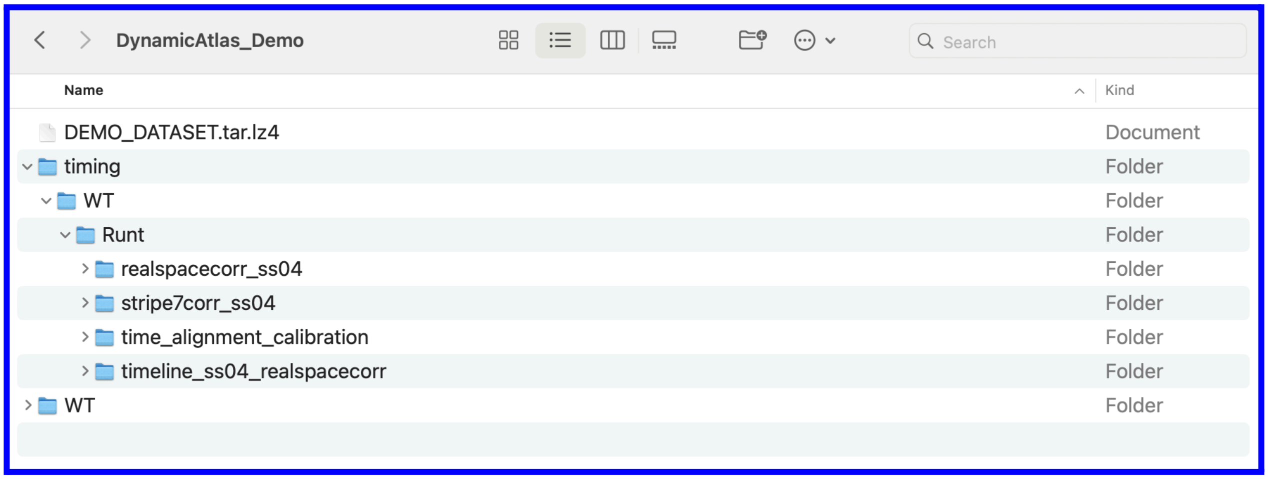

The folder ‘realspacecorr_ss04’ contains data of stripe detections, image correlations, and correspondence curves computed during intermediate steps of the timeline generation.

The folder ‘stripe7corr_ss04’ contains data of the correlations between the stripes.

The folder ‘time_alignment_calibration’ contain snapshots of the timestamping of the individual live datasets to the master timeline.

The folder ‘timeline_ss04_realspacecorr’ contains data of the master timeline as a whole, including the network construction, network relaxation, and the final timestamps of the master timeline. The timeline correspondences from live datasets to the master timeline can be visually observed in ‘time_correspondences.png’’.

Importantly, within this folder, the full master timeline data is stored within the variable ‘ttc.mat’, which contains all the explicit time correspondences between all datasets to each other. Opening this variable yields:

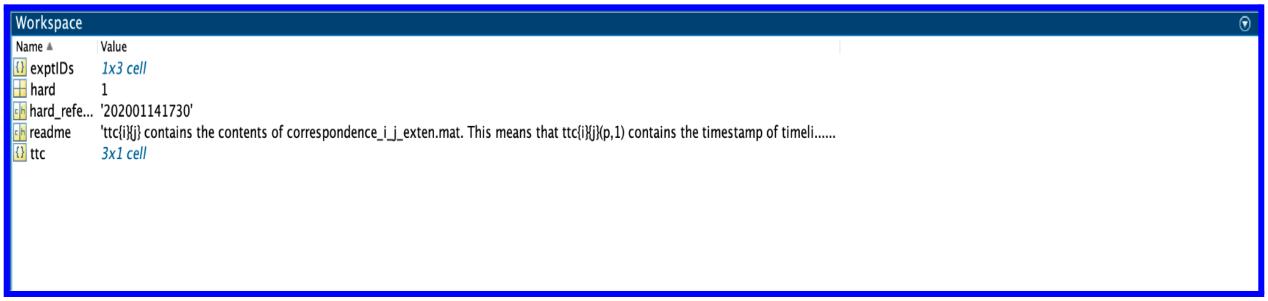

The variable exptIDs contains the experiment IDs, and their indices are given by their position in this array. The readme explains the convention used for comparisons, and ttc contains the

correspondence data. These consist of two-column arrays, with the left column representing the time frames of a selected embryo, and the right column representing the corresponding times in another embryo. An example of an array in ttc is shown below, which represents the correspondence between time frames in embryo 1 and equivalent times in embryo 3:

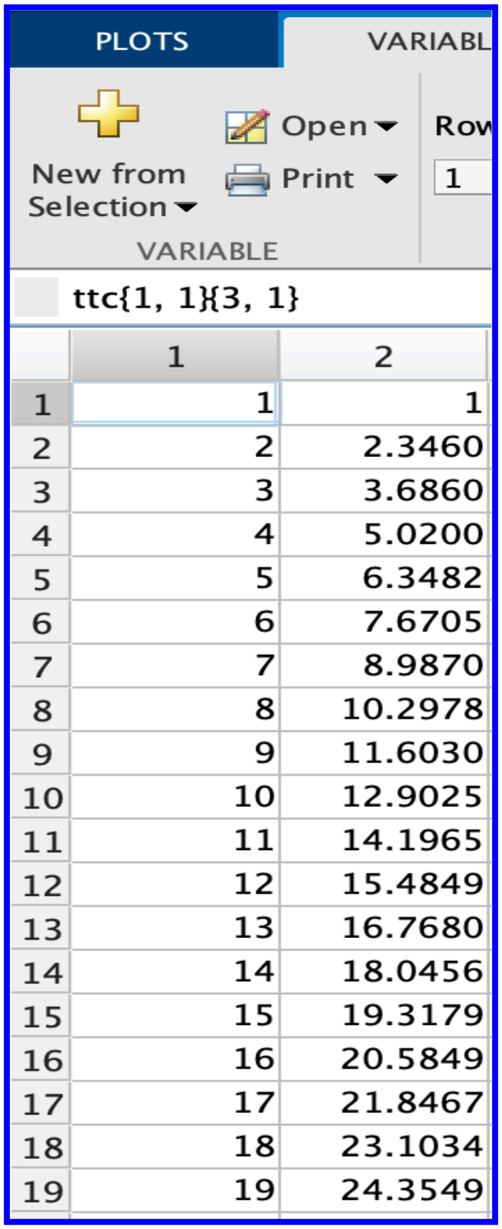

	1	2
1	1	1
2	2	2.3460
3	3	3.6860
4	4	5.0200
5	5	6.3482
6	6	7.6705
7	7	8.9870
8	8	10.2978
9	9	11.6030
10	10	12.9025
11	11	14.1965
12	12	15.4849
13	13	16.7680
14	14	18.0456
15	15	19.3179
16	16	20.5849
17	17	21.8467
18	18	23.1034
19	19	24.3549

From the saved information mentioned above, the master timeline for this series of live datasets can now be directly accessed and queried for use in other computations of choice. For example, a timeline was previously generated using this method from live data contained within the full atlas, and this is the source of the time matching data that one can query using the Python interface referenced in the main text and the Supplementary Information.

Demo code walkthrough: Fixed-data Timestamping

[Block XI]

The final step of atlas timeline creation is to timestamp a fixed dataset onto the live master timeline (see main text Figure 2). This code block performs the fixed timestamping for the fixed datasets within the ensemble.

Code block XI timestamps all the fixed Runt samples to the live-generated master timeline. (Note that in general, the fixed samples will include data from embryos that have been co-stained for another gene in addition to Runt, so this enables timestamping of other gene expression patterns by proxy – see main text Figure 2).

```
132  %% XI. Timestamp fixed data against the master timeline
133  %
134  % Timestamping Runt fixed samples against the master timeline
135  % Options can be passed through a struct if desired.
136
137  %path to the folder of the embryo chosen as the master timeline designee
138  %(done earlier in the timeline creation block X, the folder will have
139  % 'master_timeline_designee.txt' within it)
140  masterDesigneeDir = '/Users/Vishank/Documents/DynamicAtlas_Demo/WT/Runt/202001141730';
141
142  %width(s) of Gaussian(s) to use while smoothing the images
143  sigmas = [20];
144  %steps used by the gradient during the computation
145  steps = [1];
146  %specifying to compute gradients only on the fixed datasets
147  %(only fixed are timestamped, so only necessary to compute these here)
148  fixedOnly = 1;
149
150  %makes gradient images of the fixed data to use in the alignment
151  makeGradientImages(da,'Runt',sigmas,steps,fixedOnly);
152
153  %options specifying how to timestamp the fixed samples
154  Options = struct();
155  %passes in the directory of the master timeline designee
156  Options.masterDesigneeDir = masterDesigneeDir;
157  %indicating that stripe information should be loaded in from the .mat
158  %variable stored in the folder
159  Options.loadStripeMat = 1;
160
161  %timestamps fixed samples with the Runt label to the master timeline
162  da.timeStamp('WT', 'Runt', Options)
163
164  disp('Demo done.')
```

Note that the user must set their own input here (line 140 above): the path to the directory of the dataset previously chosen by the user as the master timeline designee in code block X (in this case, dataset ID: 202001141730). This folder is easy to verify by checking if it contains the ‘master_timeline_designee.txt’ file within it, as shown below:

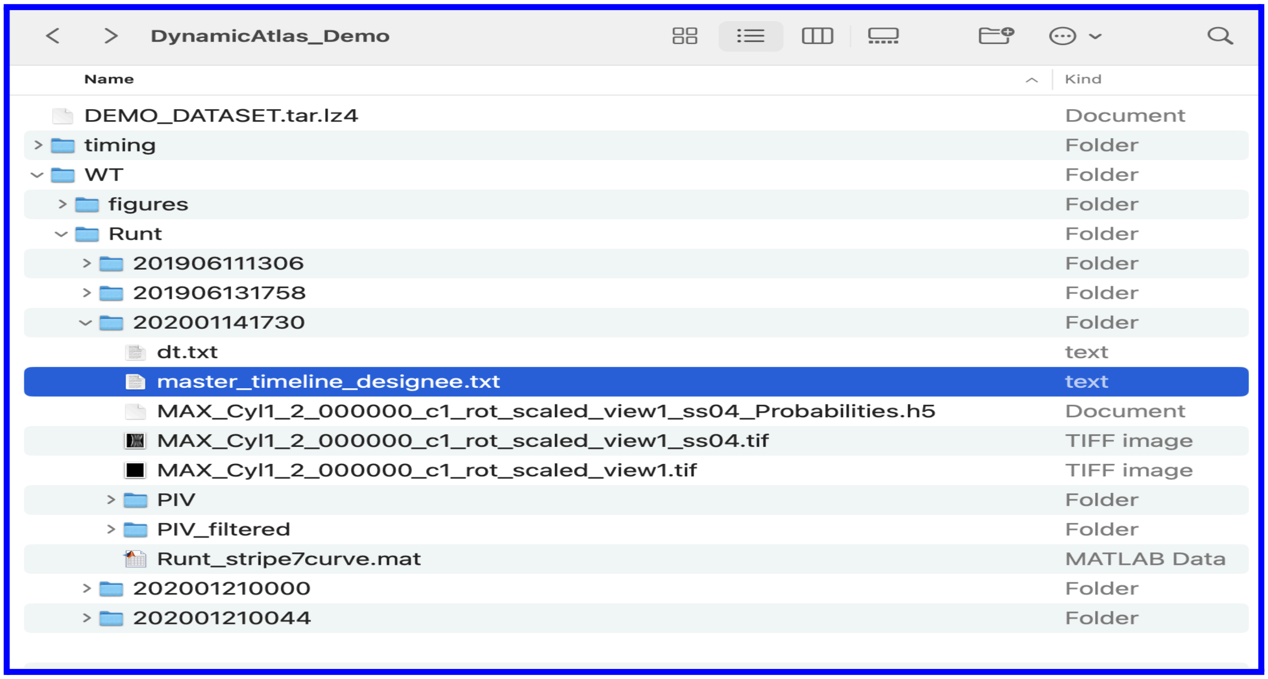

The rest of the variables in this code block do not need to be changed.

The first step of the code block is to create smoothed gradient images of the fixed datasets to be used to perform the time stamping. This is done with the function makeGradientImages, which will process the fixed datasets (here, ID: 201906111306 and 201906131758) using the sigma contained in 'sigmas' for the Gaussian and the gradient step size contained in 'steps'. When the code through makeGradientImages is run, gradient images will be saved in the folder of each fixed dataset, as shown for the fixed dataset 201906111306 and its folder 'sigma020_step001' below:

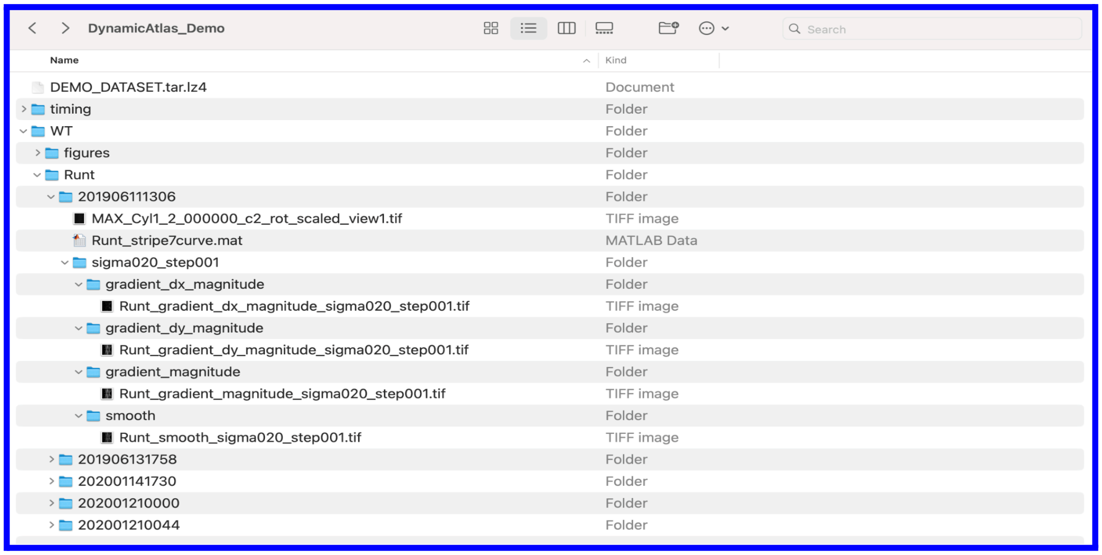

Below is a side by side comparison of the original data (MAX_Cyl1_2_000000_c2_rot_scaled_view1.tif) and the smoothed gradient image (Runt_smooth_sigma020_step001.tif) when opened in Fiji:

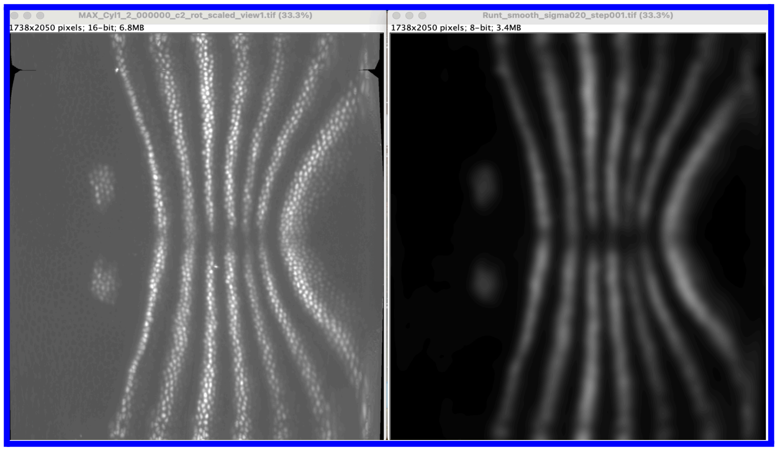

The smoothed images generated from this code are more amenable for comparison to the continuous reference timeline.

The remaining code performs the timestamping. Once run, a series of automatic computations will take place, then for each fixed dataset, a GUI window will pop up for the user to manually adjust the reference timeline coordinate to best fit the current dataset, by minimizing stripe residuals (left plot). The program provides a visual aid for this process (right plot), by displaying the dataset stripe (in orange) and the currently-selected reference timeline stripe (in blue). The instructions at the top of the left plot describe how to adjust the fit, in both position and width. The program will provide an initial guess for a timestamp, but typically the user will have to adjust this further. Below shows the initial window for dataset 201906131758:

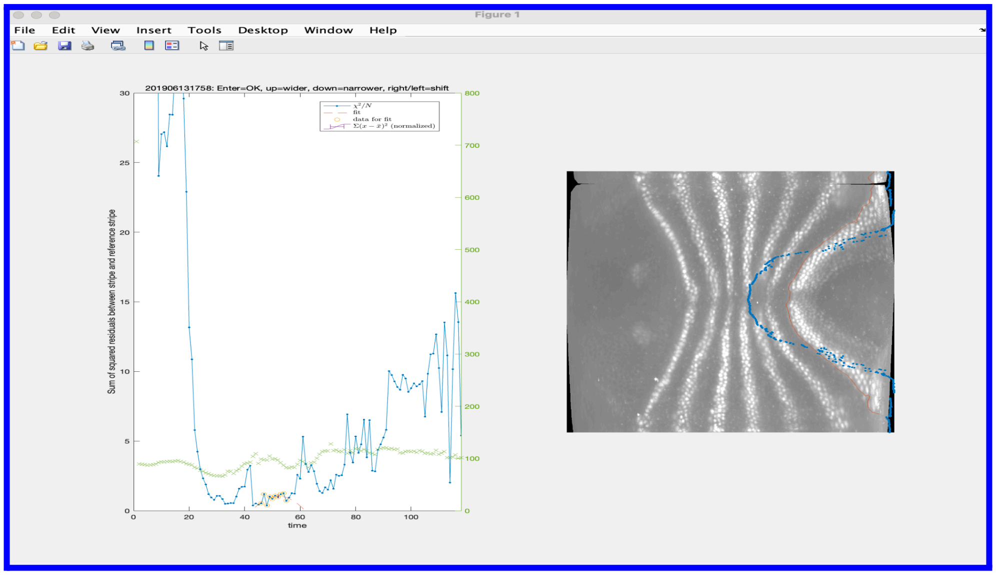

And an improved fit after user-inputted adjustments:

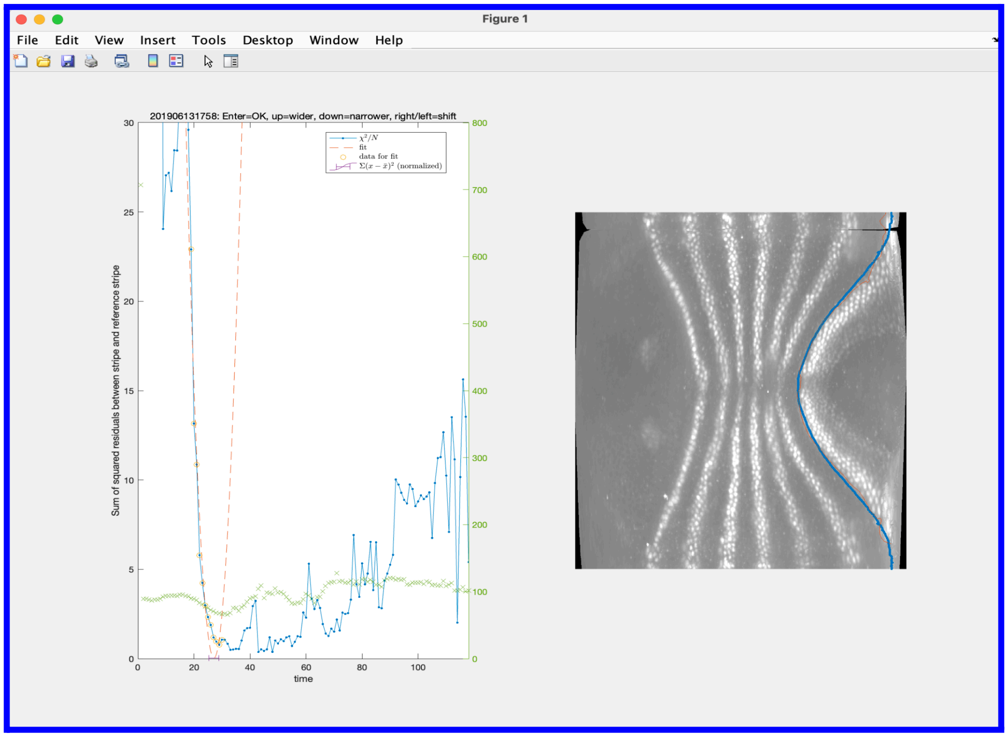

Pressing ‘Enter’ accepts the fit once it is completed. This process will be carried out for each fixed dataset. Once completed, further automatic computations will occur, and the resulting timestamps will be saved to disk. The code block, and the demo script as a whole, will then be complete, showing the following output:

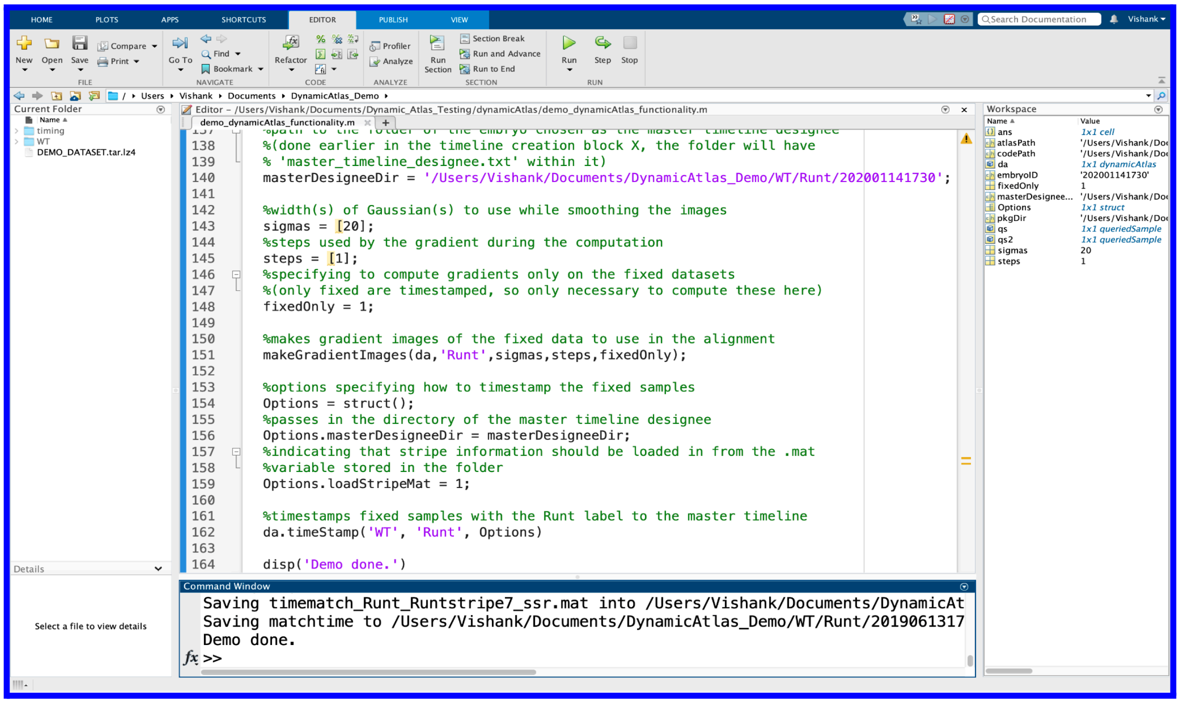

After this step is complete, the time matching results will be saved in each of the individual dataset folders. For example, for 201906111306, the following files will now be contained within:

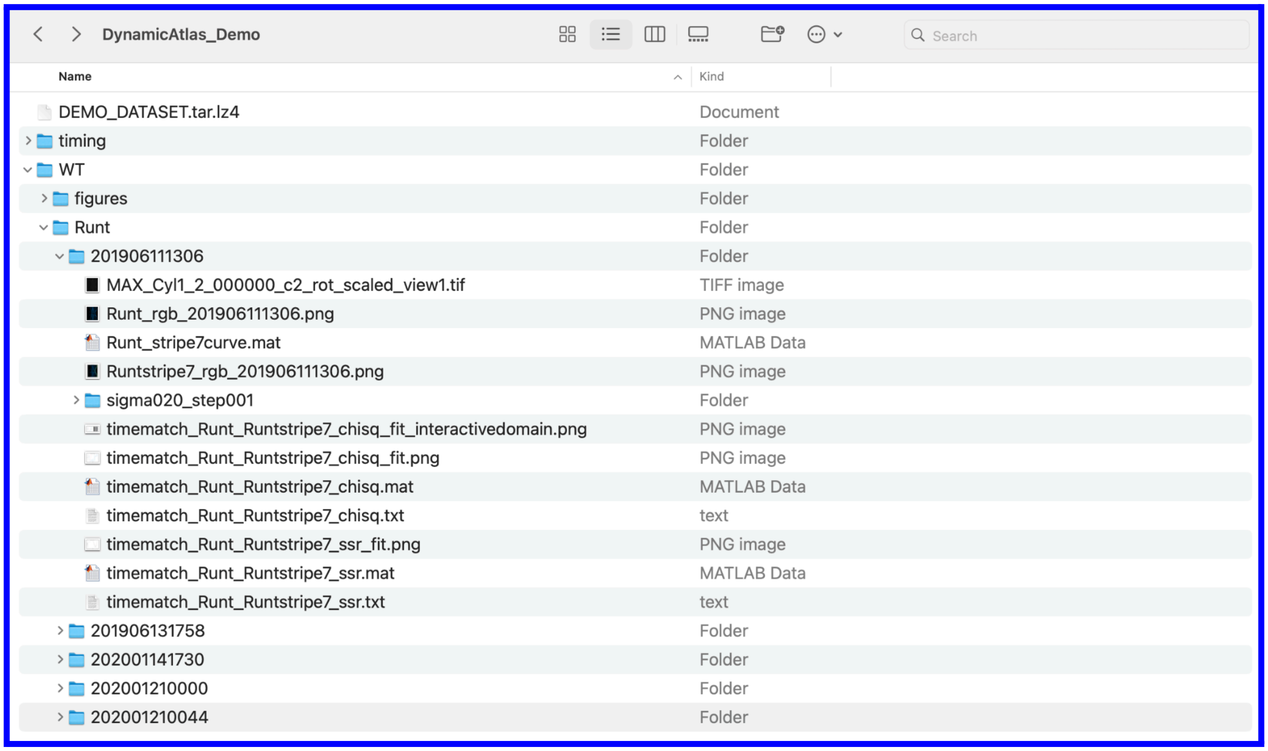

The time matching results, using the respective methods of minimizing chi-squared and sum-squared-residuals between stripes, are saved in the files prefaced by 'timematch'. Specifically, the values stored are the timestamp, and the 1 sigma (68% CI) uncertainty. Shown below is the image 'timematch_Runt_Runtstripe7_chisq_fit.png':

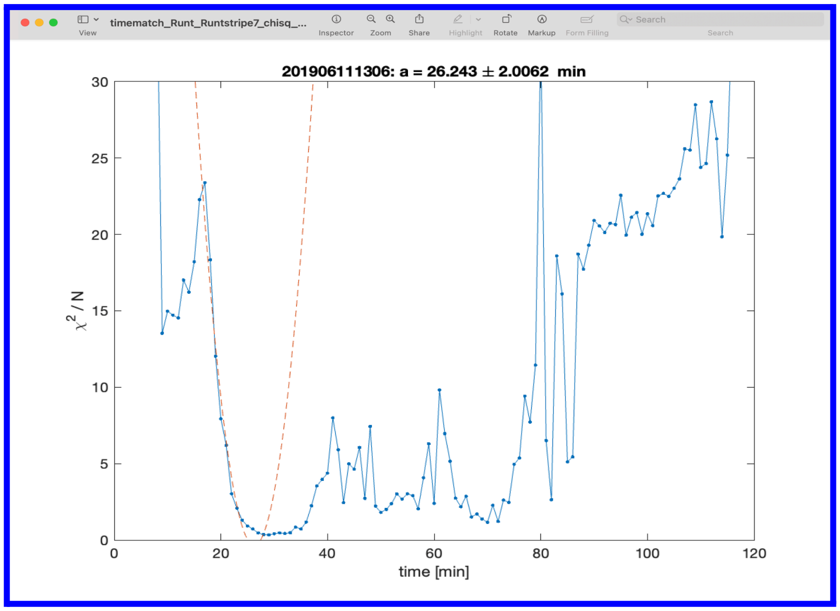

The timestamp and the fit uncertainty values are shown at the top. These are also stored in the corresponding .txt file, and .mat file (with more detail), as shown below:

.txt file:

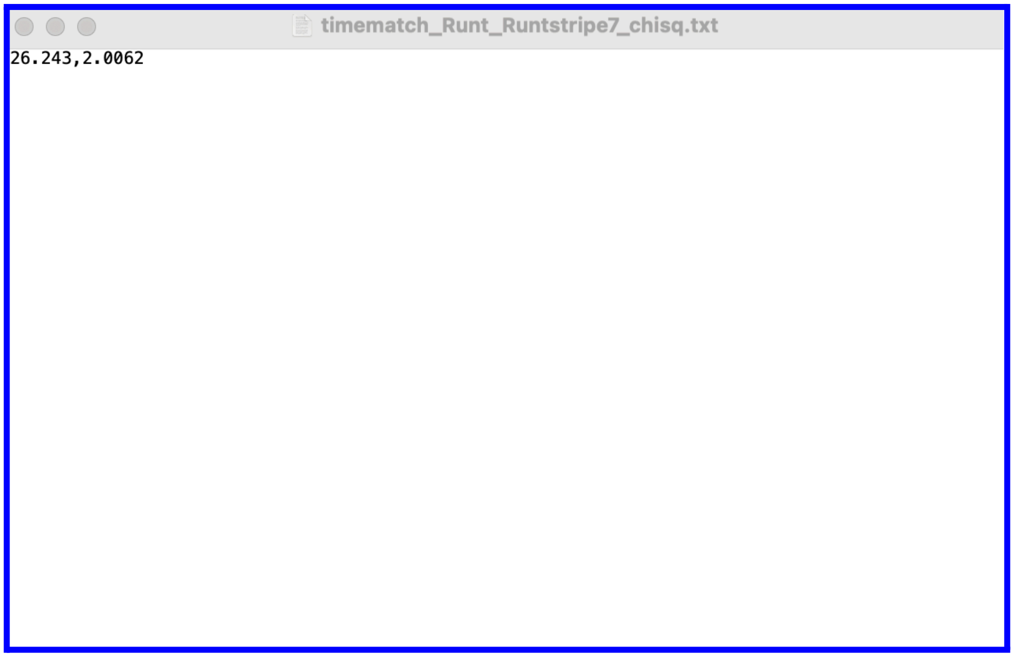

.mat file:

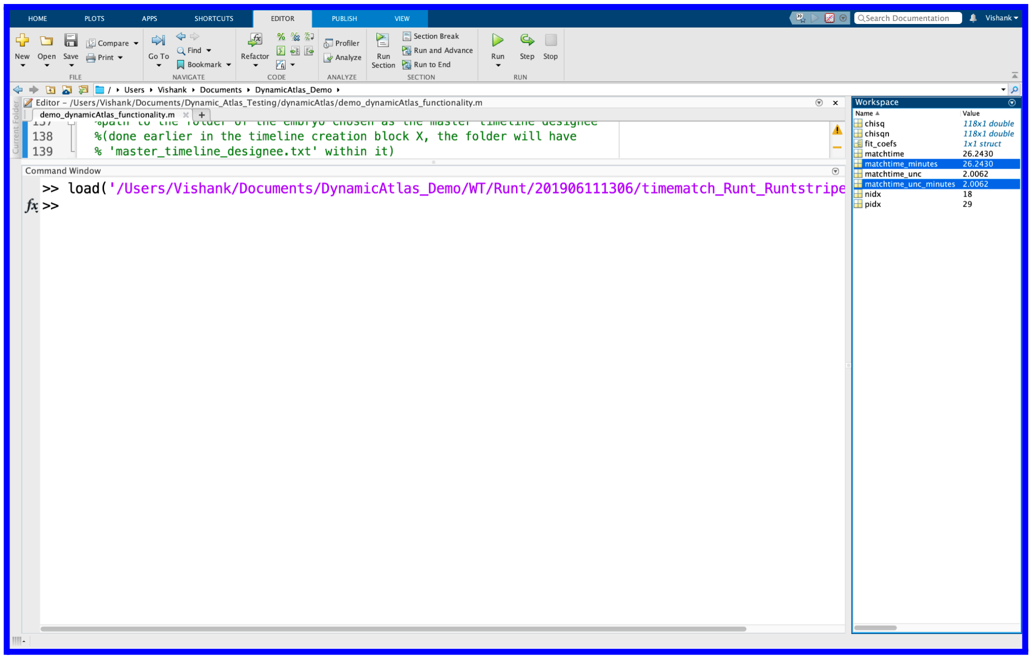

—

The demo code is now finished. All quantitative computations, and their corresponding visualizations, are stored as additional files within the existing dataset folders, or as files in the new ‘timing’ folder created during master timeline generation. The methods demonstrated in this demo are general, and other Atlas data on the Dryad repository, as well as new datasets (as long as arranged using the same conventions) can be processed similarly: to create an atlas, to create an ensemble timeline, and to timestamp fixed datasets onto the timeline.

—
