# Supplementary material for: DynamicAtlas: a morphodynamic atlas for Drosophila development
Source: Nat Methods. 2025 Dec 24;23(1):260–70. doi: 10.1038/s41592-025-02897-8 (PMC12791008; doi:10.1038/s41592-025-02897-8)
Supplement: Supplementary file 2 — Reporting Summary [file 41592_2025_2897_MOESM2_ESM.pdf]

nature portfolio

Corresponding author(s): Sebastian Streichan

Last updated by author(s): Jun 17, 2025

Reporting Summary

Nature Portfolio wishes to improve the reproducibility of the work that we publish. This form provides structure for consistency and transparency in reporting. For further information on Nature Portfolio policies, see our Editorial Policies and the Editorial Policy Checklist.

Statistics

For all statistical analyses, confirm that the following items are present in the figure legend, table legend, main text, or Methods section.

n/a	Confirmed	
☐	☒	The exact sample size (*n*) for each experimental group/condition, given as a discrete number and unit of measurement
☒	☐	A statement on whether measurements were taken from distinct samples or whether the same sample was measured repeatedly
☐	☒	The statistical test(s) used AND whether they are one- or two-sided<br>*Only common tests should be described solely by name; describe more complex techniques in the Methods section.*
☒	☐	A description of all covariates tested
☒	☐	A description of any assumptions or corrections, such as tests of normality and adjustment for multiple comparisons
☐	☒	A full description of the statistical parameters including central tendency (e.g. means) or other basic estimates (e.g. regression coefficient) AND variation (e.g. standard deviation) or associated estimates of uncertainty (e.g. confidence intervals)
☐	☒	For null hypothesis testing, the test statistic (e.g. *F*, *t*, *r*) with confidence intervals, effect sizes, degrees of freedom and *P* value noted<br>*Give P values as exact values whenever suitable.*
☒	☐	For Bayesian analysis, information on the choice of priors and Markov chain Monte Carlo settings
☒	☐	For hierarchical and complex designs, identification of the appropriate level for tests and full reporting of outcomes
☐	☒	Estimates of effect sizes (e.g. Cohen's *d*, Pearson's *r*), indicating how they were calculated

Our web collection on statistics for biologists contains articles on many of the points above.

Software and code

Policy information about availability of computer code

Data collection	Light sheet imaging data was collected using a custom MuVi SPIM confocal light sheet microscope (see doi: 10.1038/nmeth.2064 for details). For control and automation of the microscope hardware and for development of data capture scripts, the open source software package microManager (https://micro-manager.org/ (version 1.4)) was used. Additionally, the commercial software package Matlab (from release Matlab R2015a to present) was used to generate custom scripts (previously described in: doi: 10.1038/nmeth.2064) to control galvanometric mirrors of the microscope. For fusion and deconvolution of the light sheet data, open source software package ImageJ (https://imagej.net/software/fiji/ (any version released after 2014)) was used with the Multiview Deconvolution plugin (available here: https://github.com/PreibischLab/multiview-reconstruction and described previously doi: 10.1038/nmeth.2929). Additionally, the commercial software package Matlab (from release Matlab R2015a to present) was used to generate cartographic projections of light sheet data as described previously (doi: 10.1038/nmeth.3648).
Data analysis	Commercial software used: Matlab R2015a to present release. DynamicAtlas software resources, custom-developed in this publication, were used to perform computational analysis of tissue dynamics, in particular to generate time stamps and perform time alignment across datasets. DynamicAtlas code for the Python-based interface is located on a Zenodo data repository, publicly available at the following URL: "https://doi.org/10.5281/zenodo.14285126" (Version released for publication: v1). DynamicAtlas code for the MATLAB-based interface is located on a Github repository, publicly available at the following URL: "https://github.com/npmitchell/dynamicAtlas" (Version released for publication: v1.1.0).

For manuscripts utilizing custom algorithms or software that are central to the research but not yet described in published literature, software must be made available to editors and reviewers. We strongly encourage code deposition in a community repository (e.g. GitHub). See the Nature Portfolio guidelines for submitting code & software for further information.

Data

Policy information about availability of data

All manuscripts must include a data availability statement. This statement should provide the following information, where applicable:

- Accession codes, unique identifiers, or web links for publicly available datasets
- A description of any restrictions on data availability
- For clinical datasets or third party data, please ensure that the statement adheres to our policy

The experimental data contained within the atlas (including the live datasets used to perform analysis of germband extension) are available on a public Dryad Repository at the following URL: "https://doi.org/10.25349/D9WW43". This includes the spreadsheet of metadata used by the Python interface to query data (MorphodynamicAtlas.csv). We have also included a minimal `demo dataset' (DEMO_DATASET.tar.lz4), used in the MATLAB tutorial contained in the Supplementary Information. This demo dataset is located on a Zenodo data repository, publicly available at the following URL: "https://doi.org/10.5281/zenodo.14792464".

Human research participants

Policy information about studies involving human research participants and Sex and Gender in Research.

Reporting on sex and gender	N/A
Population characteristics	N/A
Recruitment	N/A
Ethics oversight	N/A

Note that full information on the approval of the study protocol must also be provided in the manuscript.

Field-specific reporting

Please select the one below that is the best fit for your research. If you are not sure, read the appropriate sections before making your selection.

☒ Life sciences ☐ Behavioural & social sciences ☐ Ecological, evolutionary & environmental sciences

For a reference copy of the document with all sections, see nature.com/documents/nr-reporting-summary-flat.pdf

Life sciences study design

All studies must disclose on these points even when the disclosure is negative.

Sample size	Our atlas consists of 500 unique live and fixed embryo datasets, including 18 mutant genotypes, detailed on Pages 8-9 of the Supplementary Information in Supplementary Table 1. This sample size was determined by the total number of Drosophila embryos imaged and post processed that passed the quality control standard described below (Data exclusions section). Given the reproducibility of Drosophila tissue dynamics across embryos, this sample size is sufficient to robustly study dynamics of morphogenetic processes across genotypes.
Data exclusions	For this study, experimental data inclusion/exclusion decisions were based on pre-established criteria of image and sample quality. All of the data included in this study was acquired on a custom light-sheet microscope. Certain visibly obvious imaging and fusion artifacts (described in detail on Page 11 of the Supplementary Information in Supplementary Note 3) specific to in-toto light sheet microscopy can occur infrequently using the data collection methodologies we employed. To account for these infrequent effects (which visibly and obviously manifest during the multi-view fusion and cartographic surface projection phases of data processing), datasets that did not successfully fuse and/or datasets in which the surface projection was not optimal were excluded from inclusion in the atlas.
Replication	For all experimental classes included in the atlas (including different Drosophila mutants analyzed and different fluorescent markers analyzed), a minimum of three independent embryos were included to increase replicability. All attempts at replication were successful.
Randomization	Not applicable. This work describes a non-biased atlas of protein expression and tissue flow during Drosophila gastrulation and midgut morphogenesis. Drosophila embryos were segregated into genotypic classes and further segregated by which fluorescently tagged proteins or immunofluorescent targets were used during imaging.
Blinding	Blinding is not relevant for this study because we are generating a descriptive atlas of protein expression and tissue flow during Drosophila embryogenesis. This study does not address a particular hypothesis, but rather provides an unbiased dataset of protein expression and tissue flow during Drosophila embryogenesis in wild type and mutant strains.

Reporting for specific materials, systems and methods

We require information from authors about some types of materials, experimental systems and methods used in many studies. Here, indicate whether each material, system or method listed is relevant to your study. If you are not sure if a list item applies to your research, read the appropriate section before selecting a response.

Materials & experimental systems

n/a	Involved in the study
☐	☒ Antibodies
☒	☐ Eukaryotic cell lines
☒	☐ Palaeontology and archaeology
☐	☒ Animals and other organisms
☒	☐ Clinical data
☒	☐ Dual use research of concern

Methods

n/a	Involved in the study
☒	☐ ChIP-seq
☒	☐ Flow cytometry
☒	☐ MRI-based neuroimaging

Antibodies

Antibodies used

PRIMARY ANTIBODIES: Rabbit anti-Even-Skipped: Gift from Mark Biggin. Guinea Pig anti-Runt: Gift from Wieschaus lab. Mouse anti-Paired: Gift from Nipam Patel (Pax3/7 DP312). Rabbit anti-Sloppy Paired: Gift from Mark Biggin (Rabbit 20257). Rat anti-Hairy: Gift from Wieschaus Lab (Rat 674). Rabbit anti Fushi-Tarazu: Gift from Mark Biggin (Rabbit 11175). Rat anti-Toll6: Gift from Liqun Luo (residues 62-81 RPLTAGAGGDPSLYDAPDDC). Rabbit anti-Tartan: Gift from Wieschaus Lab. Rabbit anti-Bazooka: Gift from Wieschaus Lab. Mouse Anti-Neurotactin: Developmental Studies Hybridoma Bank (Cat # BP106). Rabbit Anti-GFP: Invitrogen (Cat # A11122). Rat Anti-E-cadherin: Developmental Studies Hybridoma Bank (Cat # DCAD2).

SECONDARY ANTIBODIES: Donkey and goat secondary antibodies conjugated to Alexa Fluor 488, 568, 647. Supplied by ThermoFisher Scientific:

Donkey: Alexa Fluor 488 (Catalog # A-21206, RRID AB_2535792), Alexa Fluor 568 (Catalog # A10042, RRID AB_2534017), Alexa Fluor 647 (Catalog # A-31573, RRID AB_2536183)

Goat: Alexa Fluor 488 (Catalog # A-11008, RRID AB_143165), Alexa Fluor 568 (Catalog #A-11011, RRID AB_143157), Alexa Fluor 647 (Catalog # A-21244, RRID AB_2535812)

Validation

All antibodies except Rabbit Anti-GFP are generated against Drosophila melanogaster proteins. All antibodies have been validated by the manufacturer, and/or in corresponding publications, as applicable. Listed below (information also contained in Supplementary Table 2 on Page 10 of the Supplementary Information):

GFP (Rabbit, Invitrogen A11122) Validated by manufacturer and referenced in 2052 publications: https://www.thermofisher.com/antibody/product/GFP-Antibody-Polyclonal/A-11122
Even-Skipped (Rabbit, Gift from Mark Biggin, Rabbit #10900) Referenced in Perry et al., Current Biology 2012.
Runt (Guinea Pig, Gift from Wieschaus Lab)
Paired (Pax 3/7 DP312) (Mouse, Gift from Nipam Patel) Referenced in Davis et al., Developmental biology 2005.
Sloppy Paired 1 (Rabbit, Gift from Mark Biggin, Rabbit #20257)
Hairy (Rat, Gift from Wieschaus Lab, Rat #674)
Fushi Tarazu (Rabbit, Gift from Mark Biggin, Rabbit #11175)
Toll-6 (residues 62 to 81 RPLT-AGAGGDPSLY-DAPDDC) (Rat, Gift from Liqun Luo) Referenced in Ward et al., Neuron 2015.
Tartan (Rabbit, Gift from Wieschaus Lab) Referenced in Lefebvre et al., eLife 2023.
Bazooka (Rabbit, Gift from Mo Weng) Referenced in Gu et al., Molecular Biology of the Cell 2024
Nerotactin (Mouse, DSHB BP106) Referenced in 20 publications: https://dshb.biology.uiowa.edu/BP-106-anti-Neurotactin
E-cadherin (Rat, DSHB DCAD2) Referenced in 48 publications: https://dshb.biology.uiowa.edu/DCAD2

Animals and other research organisms

Policy information about studies involving animals; ARRIVE guidelines recommended for reporting animal research, and Sex and Gender in Research

Laboratory animals

Drosophila melanogaster: Laboratory strain: Oregon R (BDSC (#5)). Data included in the manuscript includes images of Drosophila melanogaster embryos 3-4 hours post fertilization (stages 6-8), 7-9 hours post fertilization (stage 12), and 13-15 hours post fertilization (stages 15-16). Both male and female animals were used; embryo sex could not be determined at these stages. Other strains used are listed below (also listed in Supplementary Table 1 on Pages 8-9 of the Supplementary Information):

UAS-Baz::GFP (Krahn et al., Current Biology 2010)
Klar SqhGFP Tl[rm9] (Gift from Wieschaus Lab)
Klar SqhGFP Spz[4] (Gift from Wieschaus Lab)
sqh1 FRT101/FM7; P{w+ sqh-sqhAE::GFP}attP1 (Gift from Adam Martin; Vasquez et al., Journal of Cell Biology 2014)
Halo [DF2L] snail [IIG05] / CyO, Sqh-GFP (Gift from Adam Martin; Martin et al., Nature 2009)

Halo [DF2L] twist [ey53] / CyO, Sqh-GFP (Gift from Adam Martin; Martin et al., Nature 2009)
P{sGMCA-MoeGFP}on III (Kiehart et al., The Journal of cell biology, 2000)
endo-Ecad::GFP (BDSC (#60584))
w; ubi-DE-cad::GFP (Oda et al, Journal of cell science, 2001)
w; ui-DE-Cad::GFP shg[R69]; Sqh::mCherry[M1] (Gift from Adam Martin; Martin et al., Nature 2009)
yw; sqh-sqh::mCherry[B1] (Gift from Adam Martin; Martin et al., Nature 2009)
w ;; sqh-sqh::mCherry[A11] (Gift from Adam Martin; Martin et al., Nature 2009)
Toll-8::SYFP2 (Gift from Jennifer Zallen; Paré et al, Nature 2014)
Even-Skipped::SYFP2 (Ludwig et al, PLoS genetics 2011)
P{ubi-GFP::rock}/TM3 (Gift from Yohanns Bellaiche; Bardet et al., Developmental cell 2013)
H2A::RFP (Gift from Wieschaus Lab)
H2Av::mCherry (Streichan Lab; Krzic et al., Nature Methods 2012)
sqh-utr::mCherry/ CyO (Gift from Thomas Lecuit; Rauzi et al., Nature 2010)
yw sqh[1] FRT101/FM7; P{w+ sqh-TS::GFP}attP40 (Gift from Adam Martin; Vasquez et al., Journal of Cell Biology 2014)
sqh[Ax3]; P[w+ sqh-gfp]42 (Royou et al., Journal of Cell Biology 2002)
sqh–GFP::ROCK(K116A) (Gift from Jennifer Zallen; de Matos Simões, Developmental cell 2010)
y1 w*; P{UAS-Lifeact::GFP}VIE-260B (BDSC (#35544))
Runt::LlamaTag-GFP (Gift from Hernan Garcia; Bothma et al., Cell 2018)
Tub67c-CAAX::mCherry<sqh3'UTR(attp2)/Tm3,sb (Gift from Wieschaus Lab)
w; 48Y-GAL4; klar (BDSC (#4935), klar from Wieschaus Lab)
w[*]; P{w[+mC]=UAS-mCherry.CAAX.S}2 (BDSC (#59021))
w[*]; UASp-CIBN::pmGFP; UASp-mCherry::CRY2-OCRL (Gift from Stephano de Renzis)
w[*]; UASp-CIBN::pmGFP; UASp-RhoGEF2-CRY2::mCherry (Gift from Stephano de Renzis)
y,P{w[+mC]=GAL4-Antp.P1.A}1,y[1]w[*];wg[Sp-1]/CyO;;klar (BDSC (#26817), klar from Wieschaus Lab)
w;; Mef2-GAL4 klar (Gift from Lucy O'Brian, klar from Wieschaus Lab)
w; UAS-LifeAct::Ruby (BDSC (#35545))
Hand-GFP; 4x HandGAL4; klar (Gift from Zhe Han, klar from Wieschaus Lab)
Bicoid[E1], Nanos[BN] / TM3, sb (Gift from Wieschaus Lab)
Bicoid[E1], Nanos[BN], Tsl[4] / TM3, sb (Gift from Wieschaus Lab)
Concertina[RC10] cn bw / CyO; T48 p[w+ sqhGFP] (Gift from Wieschaus Lab)
w; ΔJ29, Even-Skipped[r13] / CyO (Gift from Wieschaus Lab)
UAS–Fat2-RNAi (Gift from Sally Horne-Badovinac; Chanet et al., Nature communications 2017)
w; Traffic Jam-GAL4; Gap43::mCherry, sqh::GFP (Gift from Adam Martin; Chanet et al., Nature communications 2017)
UASp–Toll-2–HA (Gift from Jennifer Zallen; Paré et al, Nature 2014)
UASp–Toll8–HA (Gift from Jennifer Zallen; Paré et al, Nature 2014)
y w; P{UAS-runt.T}15 (Gift from Peter Gergen; Tracey et al., Development 1998)
UAS-Even-skipped / TM6 P[rosy+{l(3)}] (Gift from Andrea Brand; Brand et al., Development 1993)
Dpp[4] Snail[IIG05] / CyO (Gift from Wieschaus Lab)
Dpp[H46] wg[Sp-1] cn[1] bw[1]/CyO, P{dpp-P23}RP1 (BDSC (#2061))
y[1] w[*]; Pmatalpha4-GAL-VP1667; Pmatalpha4-GAL-VP1615 (BDSC (#80361))
w[*]; P{w[+mC]=His2Av-mRFP1}II.2 (BDSC (#23651))

Wild animals	No wild animals were used in the study.
Reporting on sex	N/A
Field-collected samples	No field-collected samples were used in the study.
Ethics oversight	Ethics oversight was not required for non-vertebrate studies. All experiments were conducted in the United States, in accordance with the Animal Welfare Act, Health Research Extension Act, and National Institutes of Health regulations.

Note that full information on the approval of the study protocol must also be provided in the manuscript.
